# BioModME for building and simulating dynamic computational models of complex biological systems

**DOI:** 10.1093/bioadv/vbae023

**Published:** 2024-02-20

**Authors:** Justin A Womack, Viren Shah, Said H Audi, Scott S Terhune, Ranjan K Dash

**Affiliations:** Department of Biomedical Engineering, Medical College of Wisconsin, Milwaukee, WI 53226, United States; Department of Biomedical Engineering, Marquette University, Milwaukee, WI 53223, United States; Department of Biomedical Engineering, Medical College of Wisconsin, Milwaukee, WI 53226, United States; Department of Biomedical Engineering, Marquette University, Milwaukee, WI 53223, United States; Department of Biomedical Engineering, Medical College of Wisconsin, Milwaukee, WI 53226, United States; Department of Biomedical Engineering, Marquette University, Milwaukee, WI 53223, United States; Department of Biomedical Engineering, Medical College of Wisconsin, Milwaukee, WI 53226, United States; Department of Microbiology and Immunology, Medical College of Wisconsin, Milwaukee, WI 53226, United States; Department of Biomedical Engineering, Medical College of Wisconsin, Milwaukee, WI 53226, United States; Department of Biomedical Engineering, Marquette University, Milwaukee, WI 53223, United States; Department of Physiology, Medical College of Wisconsin, Milwaukee, WI 53226, United States

## Abstract

**Summary:**

Molecular mechanisms of biological functions and disease processes are exceptionally complex, and our ability to interrogate and understand relationships is becoming increasingly dependent on the use of computational modeling. We have developed “BioModME,” a standalone R-based web application package, providing an intuitive and comprehensive graphical user interface to help investigators build, solve, visualize, and analyze computational models of complex biological systems. Some important features of the application package include multi-region system modeling, custom reaction rate laws and equations, unit conversion, model parameter estimation utilizing experimental data, and import and export of model information in the Systems Biology Matkup Language format. The users can also export models to MATLAB, R, and Python languages and the equations to LaTeX and Mathematical Markup Language formats. Other important features include an online model development platform, multi-modality visualization tool, and efficient numerical solvers for differential-algebraic equations and optimization.

**Availability and implementation:**

All relevant software information including documentation and tutorials can be found at https://mcw.marquette.edu/biomedical-engineering/computational-systems-biology-lab/biomodme.php. Deployed software can be accessed at https://biomodme.ctsi.mcw.edu/. Source code is freely available for download at https://github.com/MCWComputationalBiologyLab/BioModME.

## 1 Introduction

The progress of biological research has mainly relied on experimental investigations but is becoming increasingly coupled to computational modeling. Biological systems and their associated regulatory mechanisms and disease processes are intrinsically complex, often encompassing many interacting and redundant components that have become too exhaustive to assess experimentally. In addition, many of these network components are unintuitive in nature, requiring alternative approaches to determine the underlying functions of the network. Computational modeling, the process of simulating complex systems *in silico*, has emerged as a powerful tool, generating testable hypotheses of the system by predicting different possible outcomes of the system in response to perturbations. However, the technical knowledge required to develop, program, and solve computational models is a barrier for most experimental and clinical investigators. As such, there is an emergent need for software tools which can package computational modeling in a language that the broader research community can understand and use to answer complex questions. To meet this need, we have developed “BioModME,” a standalone R-based web application using the Shiny library, providing an intuitive and comprehensive graphical user interface to help investigators build, solve, visualize, and analyze computational models of complex biological systems.

## 2 Method

BioModME is developed using the open-source R programming language, chosen for its expansive libraries for creating application programming interfaces, ability to handle large datasets, and perform complex analyses. The Shiny library allows R users to create interactive web applications directly from R code converting it to HTML, CSS, and JavaScript, the standard languages for web development. Additionally, R provides a robust framework that can seamlessly incorporate C, C++, and Fortran codes. This flexibility allows developers to utilize the power of these languages within an R environment, providing the statistical power of R and the raw performance of lower-level languages for computationally intensive tasks. Our software incorporates the “Shiny” (Chang *et al.* 2021) and “bs4dash” (Granjon 2019) packages to build its web framework using AdminLTE3, a fully responsive administration template based on the Bootstrap 4.6 framework. Graphical visualizations are generated using R libraries (“ggplot2” (Wickham 2011), “Plotly” (Sievert 2020)) that incorporate JavaScript plugins to create interactive and customizable plots. Interactive, editable data tables were created using well-known JavaScript libraries supplied from the “DT” or “rhandsontable” libraries, depending on the functionality of the displayed dataset. Mathematical and reaction equations are displayed using MathJax, a JavaScript display engine for mathematics in web browsers. Open-source Fortran code is utilized for the ordinary differential equations (ODEs) or differential algebraic equations (DAEs) solvers, through the “deSolve” (Soetaert *et al.* 2010) library, allowing for constructed biological models to be solved in a precise and timely manner. This library includes multiple functions such as lsoda, lsode, lsodes, daspk, euler, rk4, ode23, and ode45 that implement different algorithms for solving a system of ODEs. For the computationally intensive process of parameter estimation, we use a modified Levenberg–Marquardt algorithm utilizing the “minpack.lm” (Elzhov *et al.* 2016) library and sum of squared objective function. The application allows users to define units for variables in the model for the following: count, duration, energy, flow, length, mass, power, pressure, speed, and temperature, with these units being defined using the “measurements” library. This library also provides basic unit-to-unit conversion, while custom code was injected to handle more complex unit conversions.

## 3 Results and discussion

BioModME is a standalone R-based web application package for building and simulating ODE/DAE-based models ranging from basic biological networks to more complex multi-region biological systems. It provides the user with an all-encompassing and code-free computational model building and simulation tool to define reaction networks, develop and simulate computational models, and analyze the behavior of biological systems. This software was developed with modules that allow the user to systematically build computational models using a graphical user interface (GUI), without requiring the knowledge of a computer programming language. The software converts the reaction network to its appropriate mathematical equations (ODEs and/or DAEs), allowing non-expert users, including bench biologists, clinicians, and students, to avoid tedious mathematical derivations needed to describe their systems. R visualization packages are used to present the simulation results in meaningful ways in the GUI with options to export the model information, plots, results, and underlying codes to other platforms. The application includes an embedded documentation section, allowing users to view and download documentation and tutorials, as needed.

**Figure 1.**
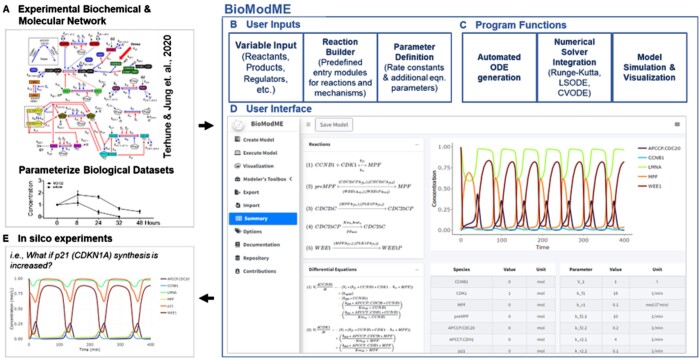
Code-free modeling and simulation with BioModME. (A) BioModME allows users with basic knowledge of biological system functions and supporting datasets to utilize this information to define model parameters and governing equations using a simple user interface. Displayed is a complex biological network of the mitotic phase of the human cell cycle (Terhune *et al.* 2020). Models can be parameterized by uploading experimental data sets. (B) The application consists of features that help guide the users through the model building process of creating variables, defining the reaction network, and inputting model parameters. (C) The application’s backend automatically formulates the governing ordinary differential and/or algebra-ic equations (ODEs and/or DAEs), solves them using appropriate ODE or DAE solvers, and provides a visualization suite for plotting the model solutions. (D) The summary page of the application displays the model’s main features for quick viewing including showing text versions of the reaction network and mathematical equations, variable and parameter tables, and a graphical plot of the model simulation results. (E) In *silico* testing an unlimited number of variables can review critical model nodes that result in the desired outcome to aid in in vitro experimental design. An example of increasing the rate of p21 (CDKN1A) synthesis and its impact on progression through mitosis is shown.

BioModME begins with a model building suite that allows the user to create reaction networks by defining key model features such as compartments, species, reactions, and flows, such as the eukaryotic mitotic cell cycle (Fig. 1A and B). This suite displays these features in interactive data tables allowing users to organize, edit, provide notes, and filter their model information. All variables are pre-generated with units and relative descriptions, such as reaction information. The program supports building ODE/DAE-based models of complex biological systems involving multiple regions using various chemical and physical laws, including the law of mass action and Michaelis–Menten kinetics for enzyme-catalyzed reactions. Users may build custom reaction functions, incorporating appropriate regulation mechanisms, to satisfy their model requirements. Custom rules can also be incorporated allowing for custom equations or species/time-dependent parameter values to be defined. The ODEs/DAEs are autogenerated using their associated laws/rules and solved using appropriate algorithms that can be selected by the user (Fig. 1C). Model information can be saved as a R Data Serialization (RDS) file or in Systems Biology Markup Language (SBML) (Hucka *et al.* 2018). An RDS file allows us to directly save application-specific features making it the preferred method for interacting with BioModME, while SBML is a common markup language that stores all model features and can be reloaded, but application specific features may be lost.

The visualization suite provides the users with multiple options to display the resulting simulations, including pre-built color palettes, line/axis options, and plotting themes (Fig. 1D). Model parameters can be changed in the suite allowing the users to generate different conditions and perturbations in a simple way (Fig. 1E). This suite includes the ability of users to generate and compare multiple iterations of a model under different conditions with a set of subplots and calculated solutions. Investigators can upload experimental data and overlay it on model simulations, allowing for comparison and validation of model simulations. Users can also use imported datasets to perform parameter estimation for their models.

A major goal for developing BioModME is to provide a hub for data exchange in the computational modeling field. As such, in addition to building its own models, the application can load in SBML files with a few caveats (see Supplementary material). With these models, the application can process and package the information into a variety of methods. For sharing and export, the application can export all model information tables (compartments, species, parameters, reactions, equations) in csv, xlsx, pdf, txt, HTML, or in a customizable LaTeX document. This allows for model information to be easily shared between researchers in a presentable way, allows a straightforward way to create publication-quality model tables. All reactions and equations (ODEs/DAEs) can be downloaded in the above formats as well as both presentation and content MathML. The ODE exporter provides the user with options to download single, selected equations or all equations into output files. Users may also download the underlying code for their model in a “ready to run” script in MATLAB or R programming languages. These robust export features are an advancement of BioModME compared to current applications such as CellDesigner (Funahashi *et al.* 2003) and COPASI (Hoops *et al.* 2006) (Table 1). In comparison to other similar software, such as MATLAB Symbiology and Berkeley Madonna, our application is free to use and requires no programming knowledge to use its features. Additionally, to our knowledge, our software is the only computational model building software that is available to use on an online platform. We also provide a plethora of styling options, allowing users to change the applications plotting and table styles. Functionally, we provide built in unit converters, allowing the user to use a range of unit definitions, not forcing them to convert all parameters to a base unit themselves. Using the Shiny framework, our package is available in numerous formats, allowing the user to download it to use on their local system or access it through an online server.

**Table 1.** Comparison of BioModME to existing molecular modeling tools.

Features	BioModME	COPASI	MATLAB: Simbiology	Celldesigner	Berkley Madonna
**1. Cost**	Free	Free	MATLAB License Simbiology License	Free	Price tier (Education, Academic, Professional)
**2. License**	Open Source	Open Source	Commercial	Open Source	Commercial
**3. Supported OS**	Windows, Mac, Linux	Windows, Mac, Linux	Windows, Mac, Linux	Windows, Mac, Linux	Windows, Mac
**4. Available online (No download required)**	Yes	No	No	No	No
**5. Requires programming**	No	No	Some: creating custom rules/laws	No	Some: Setting parameter/run files, building equations
**6. Import file type**	rds, SBML	SBML, COPASI	MAT files, SBML	SBML, CellDesigner XML	Madonna files
**7. Export file types**	rds, SBML, Code: R, Matlab, Python	SBML, COPASI, Madonna file, Code: C	SBML, Matlab	SBML	Self
**8. Contain built in chemical laws (#)**	Yes (9)	Yes (40)	Yes (10)	Yes (2)	No
**9. Parameter estimation**	Yes	Yes	Yes	No (uses other tools)	Yes
**10. Unit conversion**	Yes	No, has options to set different default units	Yes	No, can define unit types in application	No, application is unitless
**11. Flow diagrams**	No	No	Yes	Yes	Yes
**12. Model summary options**	HTML Document, PDF, Customizable Latex Options. Variable Tables available in txt, csv, HTML, latex, pdf	Reports in CSV and HTML	HTML Document, Model components to excel	CSV Tables	None
**13. ODE exports**	Plaintext, mathML, latex	Plaintext, mathML	Plaintext	Plaintext	Copy and paste text

In adherence to stringent user privacy standards, BioModME refrains from retaining any user model or session data on our web servers. We have implemented a convenient “Save” button at the application's interface, enabling users to securely store their models and session data locally as RDS files. These files can subsequently be imported through the designated ‘Import’ tab, facilitating effortless sharing with collaborators. The server-based architecture of BioModME not only facilitates increased ease of accessibility, but also paves the way for future advancements, including the prospect of cloud-based simulations and storage solutions. With its server-based approach and a comprehensive set of model building and exporting features, BioModME stands as an integrated application, offering numerous advantages over existing modeling platforms (Table 1).

## 4 Conclusions

BioModME provides a robust web application resource to help researchers develop mechanistic computational models of complex biological systems encompassing multiple regions or compartments without the need for complex mathematical derivations or the knowledge of coding and solving mathematical equations using a programming language. The Shiny interface allows for a guided model building process to create robust model simulations and high-quality graphics. The application provides tools for the user to develop their own reactions and rules, perform parameter estimation, and provides a built-in unit converter. There is a model comparison suite, allowing users to run simulations of the same model with different parameters simultaneously. BioModME can serve as a model data exchange hub, containing export abilities to send model information to a variety of table formats, storage languages, and prepared coding scripts. We plan to enhance the applications capabilities over time with additional tools for sensitivity analysis and provide model building GUIs where users can build pictorial pathways of their model using a JavaScript Canvas.

## Supplementary Material

vbae023_Supplementary_Data
