# Supplementary material for: BioModME for building and simulating dynamic computational models of complex biological systems
Source: Bioinform Adv. 2024 Feb 20;4(1):vbae023. doi: 10.1093/bioadv/vbae023 (PMC10918630; doi:10.1093/bioadv/vbae023)
Supplement: vbae023_Supplementary_Data [file vbae023_supplementary_data.docx]

Contents

[Introduction 3](#_Toc140560905)

[Online Availability 4](#_Toc140560906)

[Download Locally 5](#_Toc140560907)

[Install R and RStudio 5](#_Toc140560908)

[Download BioModME from GitHub 5](#_Toc140560909)

[Install R Repositories 6](#_Toc140560910)

[Start Application 7](#_Toc140560911)

[Pull Docker Image 8](#_Toc140560912)

[Download Docker 8](#_Toc140560913)

[Pull/Run From Docker 10](#_Toc140560914)

[Mathematical Derivations 14](#_Toc140560915)

[Equations 14](#_Toc140560916)

[Chemical Based Equations 14](#_Toc140560917)

[Input/Output 19](#_Toc140560918)

[Units 25](#_Toc140560919)

[Unit Conversion 25](#_Toc140560920)

[Converting Compound Units 26](#_Toc140560921)

[Unit Definitions 26](#_Toc140560922)

[Creating A Custom Law 31](#_Toc140560923)

[Creating a Custom Equation 35](#_Toc140560924)

[SBML Import 36](#_Toc140560925)

[Export Model 37](#_Toc140560926)

[Coding Languages 37](#_Toc140560927)

[MATLAB 37](#_Toc140560928)

[R 38](#_Toc140560929)

[Storage Languages 38](#_Toc140560930)

[rds 39](#_Toc140560931)

[SBML 39](#_Toc140560932)

[Model Information Tables 40](#_Toc140560933)

[LaTeX Document 40](#_Toc140560934)

[Differential Equations 41](#_Toc140560935)

[Basic Tutorial - Introduction 43](#_Toc140560936)

[Constructing Model 44](#_Toc140560937)

[Define Variables 44](#_Toc140560938)

[Create Reactions 47](#_Toc140560939)

[Equation 1 - Law of Mass Action, Reversible 47](#_Toc140560940)

[Equation 2 - Michaelis-Menten Kinetics 48](#_Toc140560941)

[Equation 3 - Law of Mass Action, Unidirectional 49](#_Toc140560942)

[Equation 4 - Law of Mass Action, Unidirectional 50](#_Toc140560943)

[Equation 5 - Synthesis by Factor 50](#_Toc140560944)

[Equation 6 - Degradation by Rate 52](#_Toc140560945)

[Compartment Input/Output 53](#_Toc140560946)

[Parameters 54](#_Toc140560947)

[Differential Equations 56](#_Toc140560948)

[Solve Model 56](#_Toc140560949)

[Visualize Model 58](#_Toc140560950)

[Export 61](#_Toc140560951)

[Latex 61](#_Toc140560952)

[Multicompartment Tutorial 63](#_Toc140560953)

[Constructing Model 65](#_Toc140560954)

[Create Compartments 65](#_Toc140560955)

[Define Variables 66](#_Toc140560956)

[Add Equations 68](#_Toc140560957)

[Add Compartment Inputs/Outputs 69](#_Toc140560958)

[Parameters 81](#_Toc140560959)

[Differential Equations 83](#_Toc140560960)

[Results 84](#_Toc140560961)

[Solve Model 84](#_Toc140560962)

[Visualization 86](#_Toc140560963)

[Parameter Estimation Tutorial 88](#_Toc140560964)

[Build Model 89](#_Toc140560965)

[Visualize Model 92](#_Toc140560966)

[Perform Parameter Estimation 94](#_Toc140560967)

# Introduction

BioModME is an R based web application built using the Shiny framework for expedient biological system modeling and simulations. It provides the users with an all-encompassing and code-free computational model building tool to define reaction networks, develop and simulate computational models, and analyze the behavior of complex biological systems. This web application was developed with modules that allow the users to systematically build their own models for a biological system using the graphical user interface (GUI), without requiring the knowledge of a computer programming language. The program converts the reaction network to its appropriate mathematical equations (differential and algebraic equations; DAEs), allowing the non-expert users, including bench biologists and clinicians, to avoid all tedious mathematical derivations of the equations needed to describe the biological system. R visualization packages are used to present the simulation results in meaningful ways in the GUI with options to export the model information and results for other applications. The model can also be exported to other programing languages, such as MATLAB, R and Python.

BioModME was built using the R programming language and Shiny framework. Specific elements of the application have been developed with the use of customized HTML, CSS, JavaScript, and Python. As such, the application is provided through a variety of methods for the user’s convenience including online deployed versions, source code to be locally used, and as a docker image for local use avoiding dependencies.

Note

Using the online server is a good option that requires no download of application or knowledge of running R. The server, currently, does not offer the ability to save models/load models from a user’s repository in the cloud. It does offer the ability to download model save files locally and upload local files.

# Online Availability

BioModME (stable version) is hosted on webservers at the Medical College of Wisconsin (MCW) by the Clinical & Translational Science Institute (CTSI) of Southeast Wisconsin. It can be accessed at:

<https://biomodme.ctsi.mcw.edu/>

Additionally, a backup version (developer version) of the application can be accessed through the following:

<https://jwomack7512.shinyapps.io/Model_Builder/>

The developer version is the first author’s personal shinyapps account and will be active but has a 15-minute timeout period that users should be aware of.

# Download Locally

BioModME can be run on your local system using R.

## Install R and RStudio

R and RStudio will need to be installed (tested locally with R 4.1.1 and RStudio 1.4.1717). Please check CRAN for the installation of R (<https://cran.r-project.org>).

For the installation of RStudio, go to and follow instructions from <https://www.rstudio.com>.

## Download BioModME from GitHub

**Option 1** - Using Git Bash:

1. Open Git Bash
2. Change the current working directory to the location where you want the cloned directory.
3. Enter the following command:

- git clone https://github.com/MCWComputationalBiologyLab/BioModME.git

1. Press **Enter** to create your local clone of the application.

**Option 2** - Direct Download:

1. Navigate to <https://github.com/MCWComputationalBiologyLab/BioModME>.
2. Click the green button “<> Code”.

-
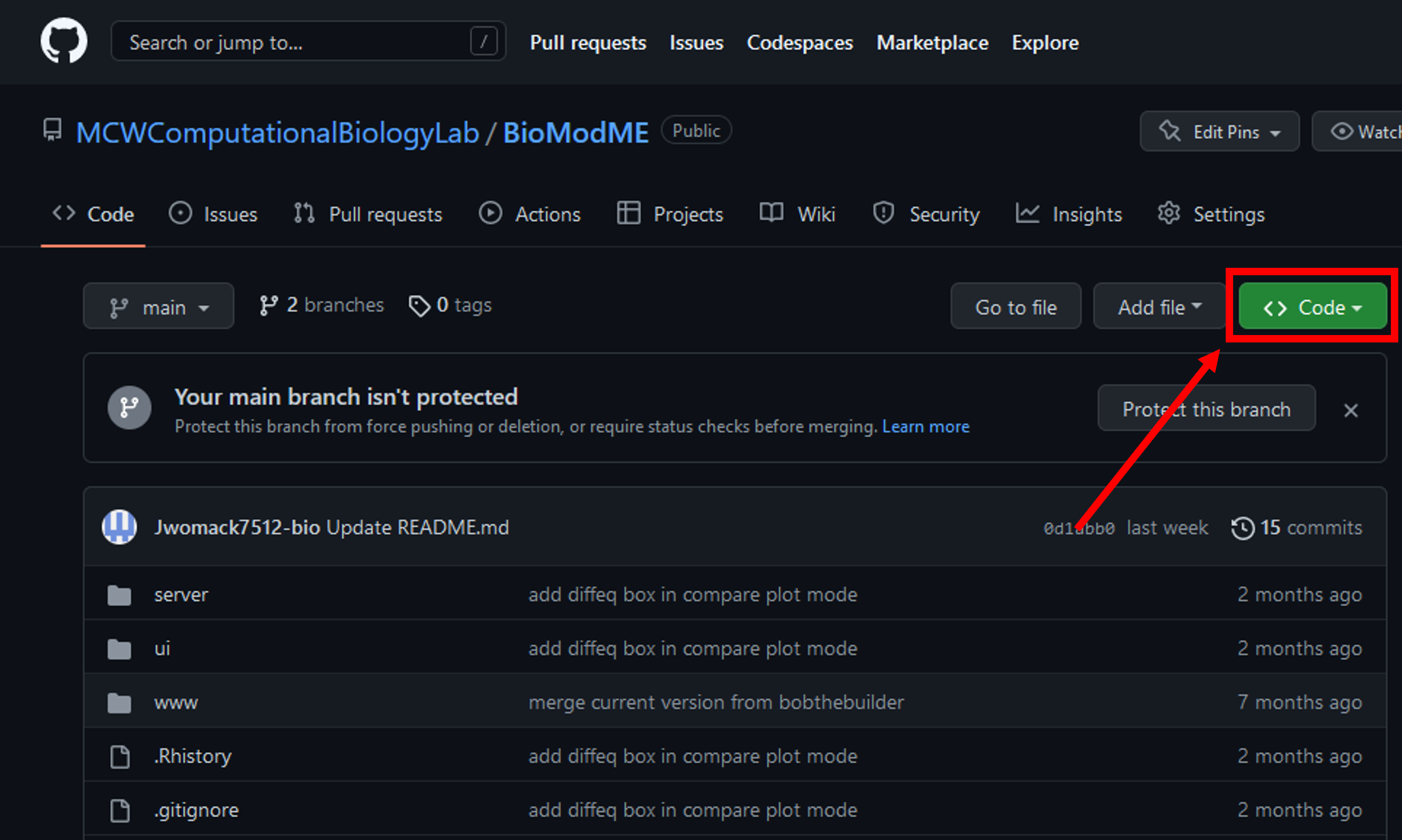


1. Press download ZIP

- [
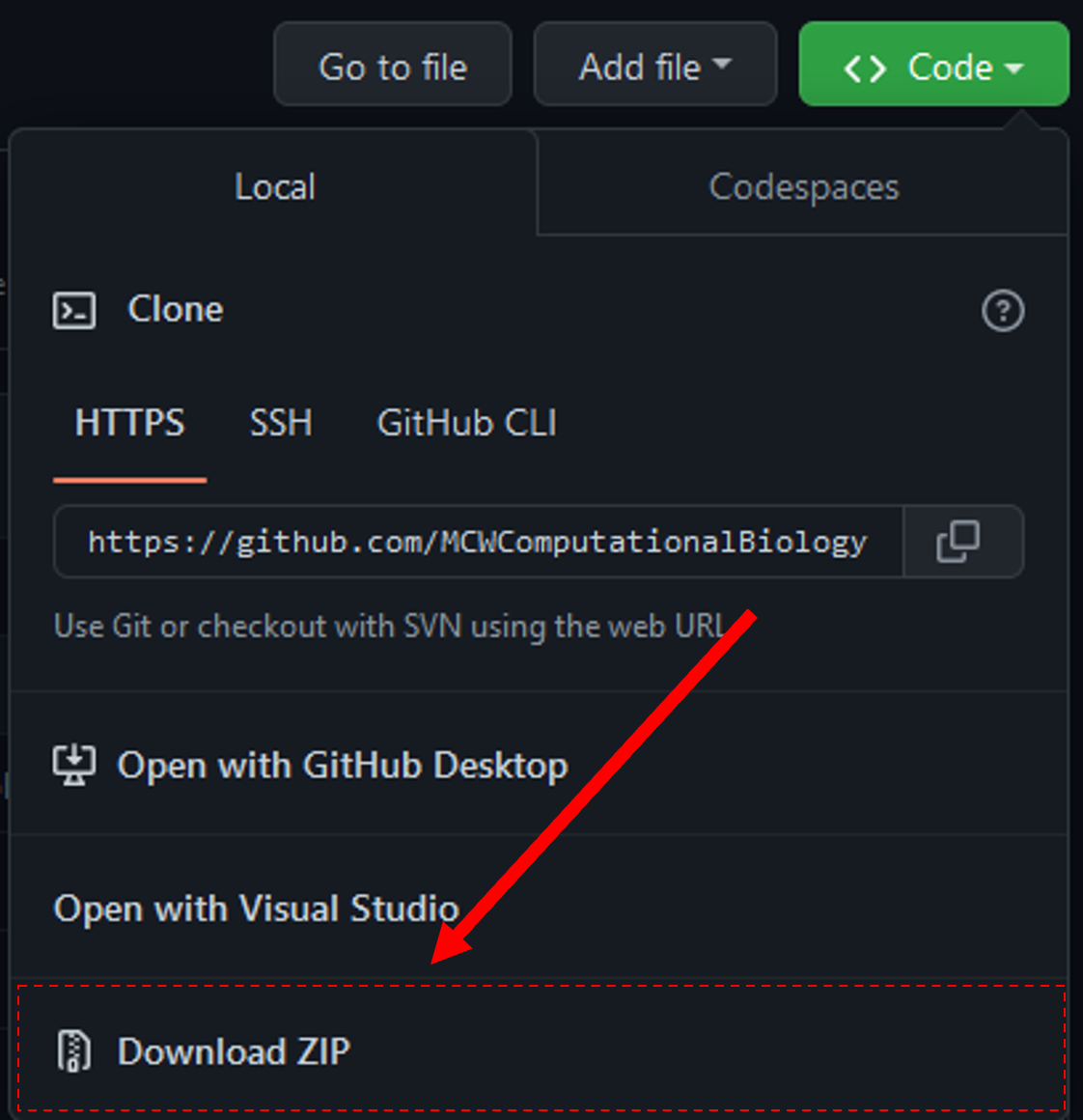
](https://mcw0-my.sharepoint.com/personal/jwomack_mcw_edu/Documents/_images/zip_download.png)

1. Unzip folder in desired location.

## Install R Repositories

BioModME uses a vast number of standard R repositories that will need to be downloaded. Open up the UI.R file from the downloaded file. This script loads all the needed libraries for the application to properly run. To install these repositories, you can run the following:

# Vector of package names to install

load.lib <- c("shinydashboard", "bs4Dash", "shiny", "ggplot2", "gridExtra", "shinythemes", "shinyWidgets", "shinyjs", "DT", "tidyverse", "dplyr", "rhandsontable", "data.table", "ggpmisc", "colourpicker", "shinyBS", "shinyjqui", "bsplus", "deSolve", "plotly", "Deriv", "viridis", "ggpubr", "shinycssloaders", "waiter", "fresh", "readxl", "minpack.lm", "measurements", "qdapRegex", "XML", "xml2", "katex", "reshape2", "clipr", "jsonlite")

# Remove any packages from list that are already installed

install.lib <- load.lib[!load.lib %in% installed.packages()]

# Install all packages in install list

for (lib in install.lib) install.packages(lib, dependencies = TRUE)

sapply(load.lib, require, character = TRUE)

All the required packages should now be installed.

Note

Current versions of RStudio will have a dropdown header telling you that you do not have all the packages installed and will provide you a button to click to install them all.

## Start Application

The application can be started from base R by navigating to the application home folder and running the following:

shiny::runApp()

A more common way to run the app is to run it directly from RStudio. This is done by:

1. Open either “ui.R” or “server.R” in RStudio
2. Press bottom in top right of script that says “Run App”.


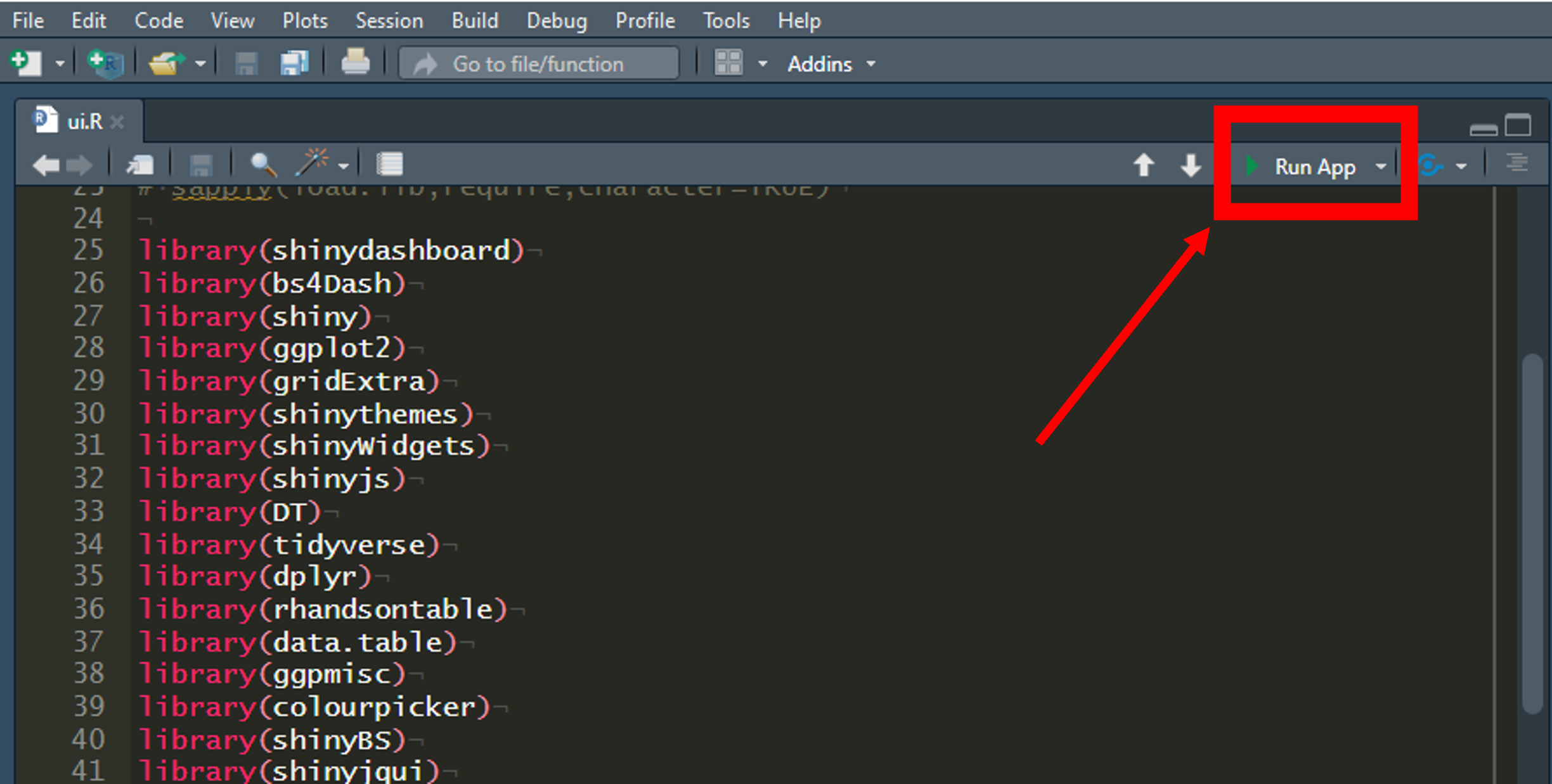


# Pull Docker Image

The application has been created as a docker image for user convenience if they wish to run the app locally without installing R or worrying about other possible dependencies.

## Download Docker

In this section, we will download docker to our computer. This will install the desktop app but what we really need is the docker terminal commands to be added. If you have a working version of Docker on your system, skip to the next section.

1. Go to <http://www.docker.com/products/docker-desktop>
2. Download docker for your respective system. This tutorial follows the windows download.

-
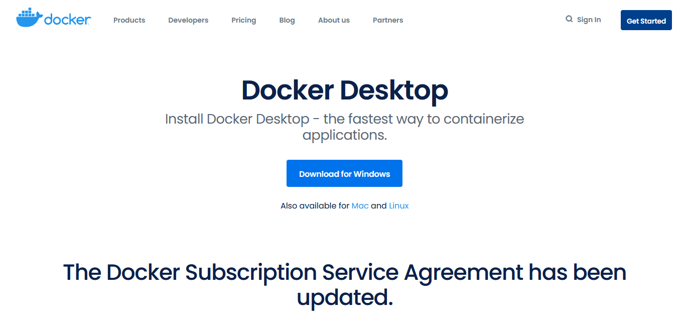


1. Go to your downloads folder and begin the system installation of docker. This download will take a few minutes.
2. A configuration popup will appear. Keep “Install required Windows components for WSL 2” checked. You can uncheck the desktop shortcut option if you want.

-
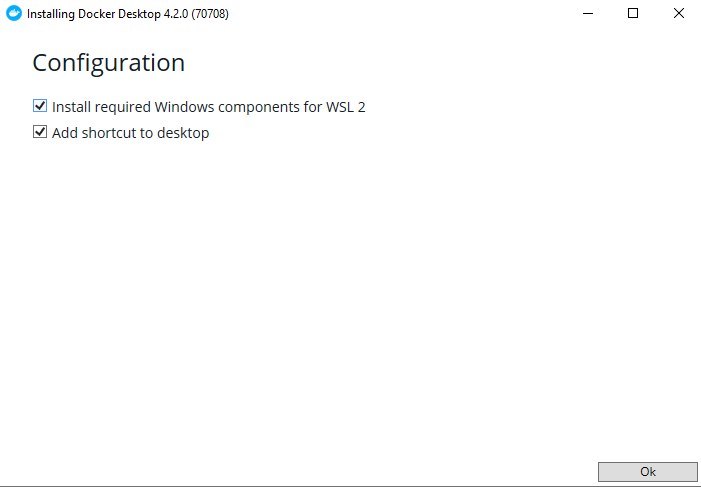


1. Once download is complete a message will appear to restart your system. Perform computer restart.
2. On restart you may get a notification that “WSL 2 installation is incomplete”. If this is the case, you will need to install this update or the docker image will not run. Follow the link on the popup, it takes you to the following link: <https://docs.microsoft.com/en-us/windows/wsl/install-manual#step-4>– -download-the-linux-kernel-update-package}{<https://docs.microsoft.com/en-us> /windows/wsl/install-manual#step-4—download-the-linux-kernel-update- package. Download and install the kernel from this link.

-
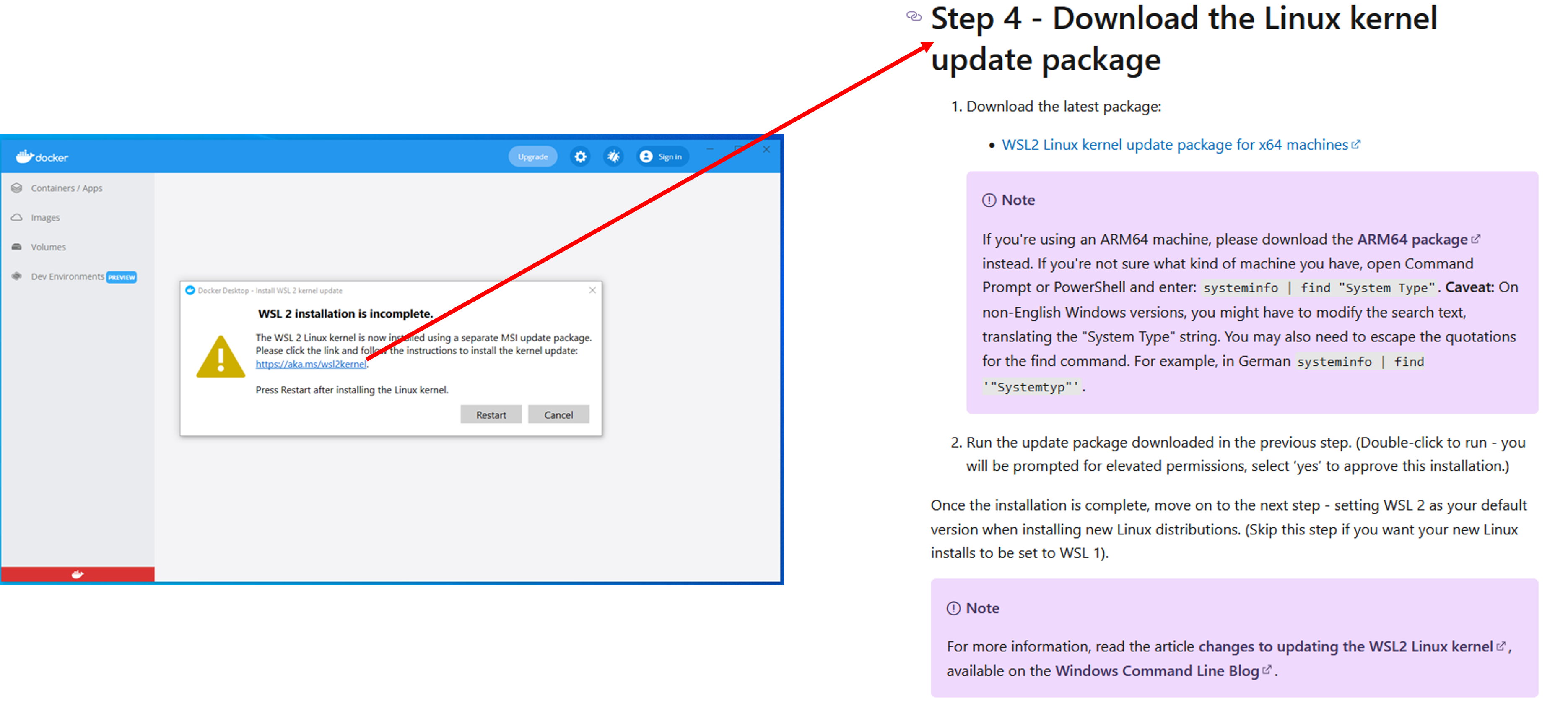


1. Restart computer again and open docker program. It should be fully installed and working now. A working program has a green color in the bottom left corner and lacks a huge error message in the center of the application.

-
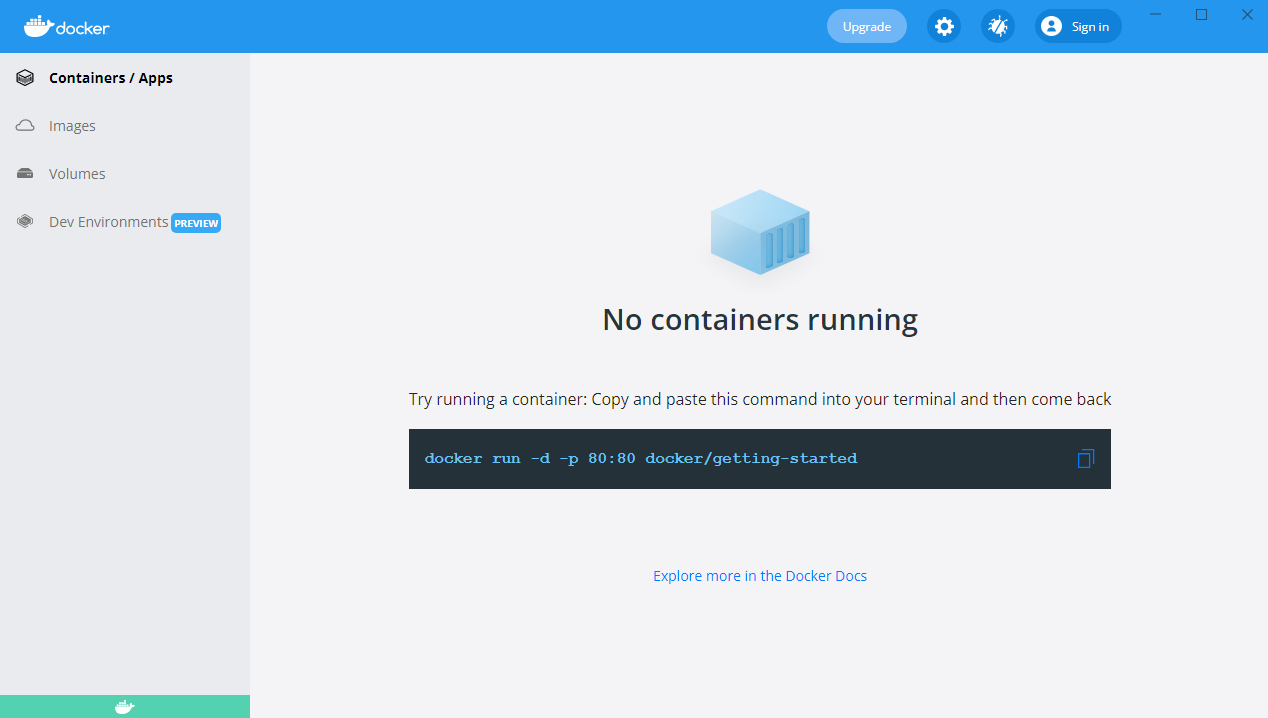


Note: A Docker account does not need to be made in order to pull and run this program.

## Pull/Run From Docker

With docker installed, we can now use the command line to pull the program we desire, meaning we can download it to our computer from the docker cloud.

1. Open the computer’s terminal (command prompt).
2. Type the below command to pull the application from the docker cloud (this takes approximately five minutes):

docker pull jwomack7512/biomodme

1. Type the following command to run the application:

docker run -dp 3838:3838 jwomack7512/biomodme

- This step might need approval from windows defender or other antivirus program.
-
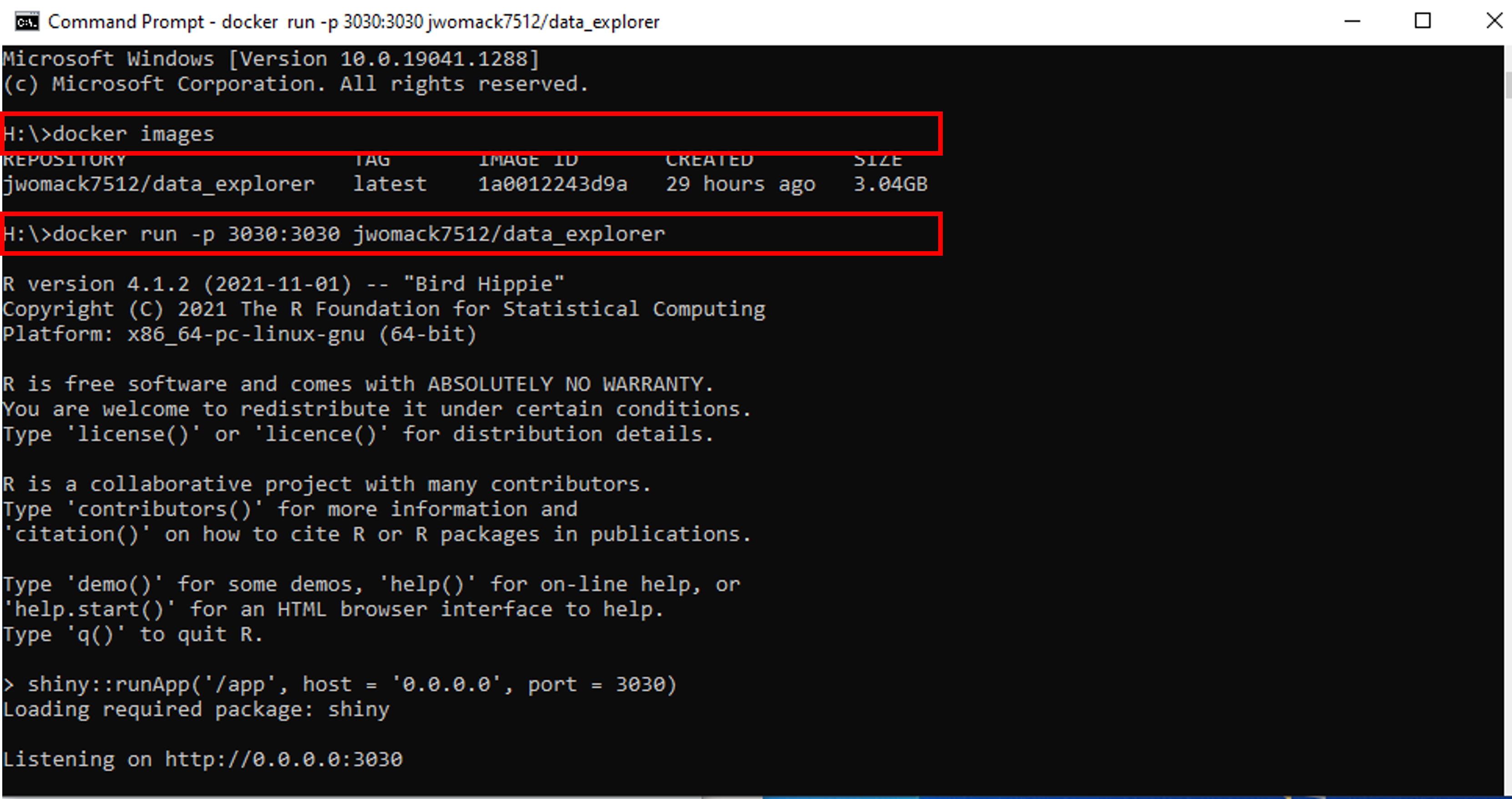


1. The above command runs the program at a local host port. To access the app, open your web browser and type the following:

- localhost:3838

1. The application should now load on the opened webpage.

#### Loading Application After Pull[](#loading-application-after-pull)

After the image has been pulled once, there is not a need to repull it. The image should be saved to your local docker program and can be loaded easily using the Docker Hub GUI.

1. Open docker and navigate to the “images” tab. Here you should see a copy of the image you pulled in the previous step. This means its available without having to repull it.
2. Scroll over the image **jwomack7512/biomodme**. A “run” button will appear. Press that button.

-
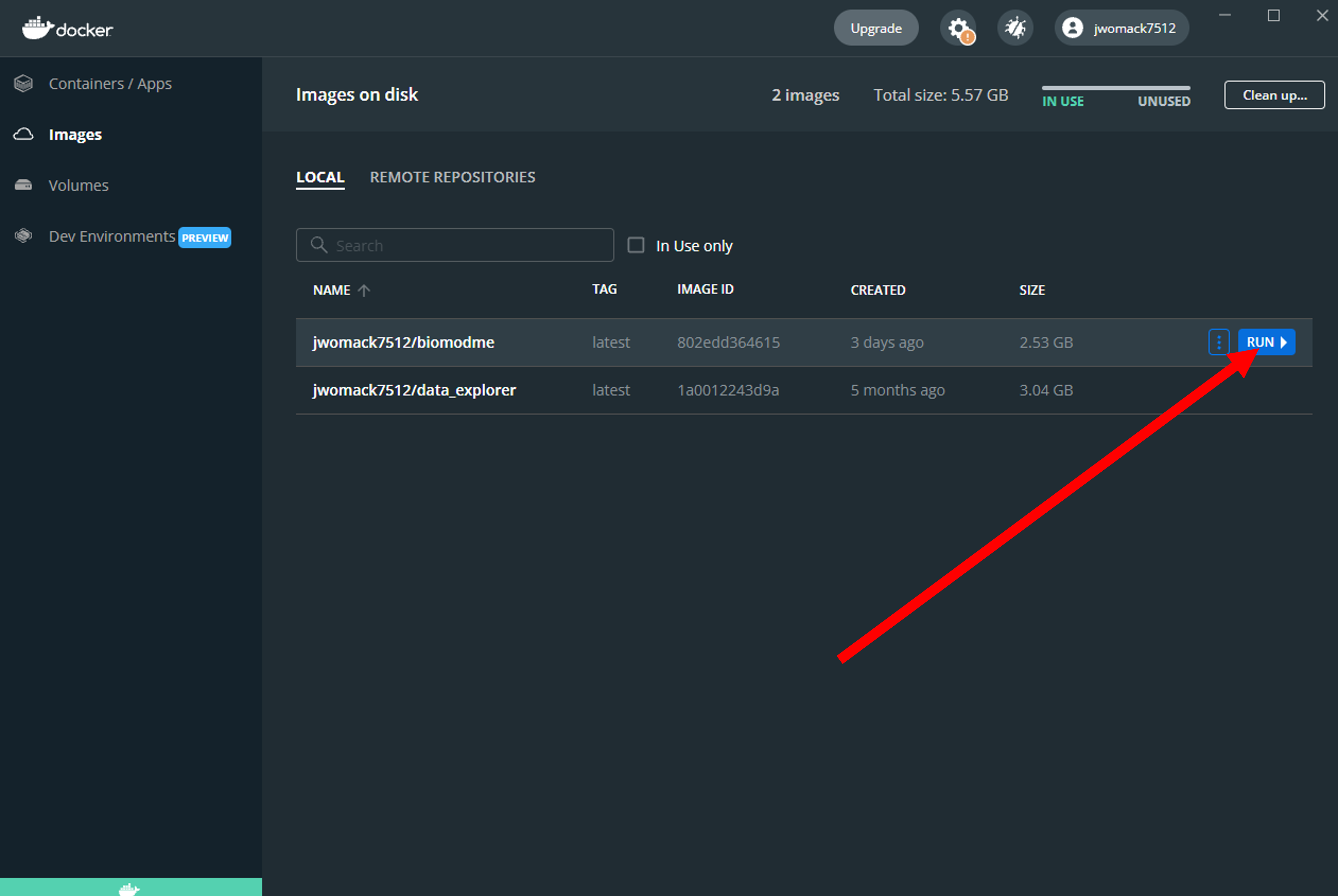


1. A popup will appear. Click the dropdown arrow to show optional settings. Here we give the container the name “bioModMe”. If you do not give it a name, docker will randomly generate one. Type in “3838” in Local Host. This is necessary or the program will not load to the proper port the app specifies. Press the “Run” button.

- [
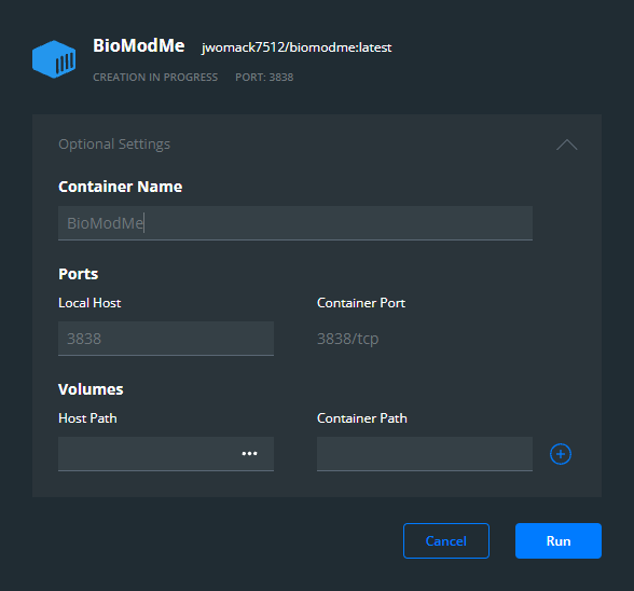
](https://mcw0-my.sharepoint.com/personal/jwomack_mcw_edu/Documents/_images/load_container_options.png)

1. Go to the “Containers/Apps” tab in Docker Hub. You should see the container you created with a note saying it is running at port 3838. You can access the app by simply clicking the “Open in Browser” button. Note: you may already have a container built from using the command line, meaning you could just run that one instead of rebuilding it in the previous steps.

-
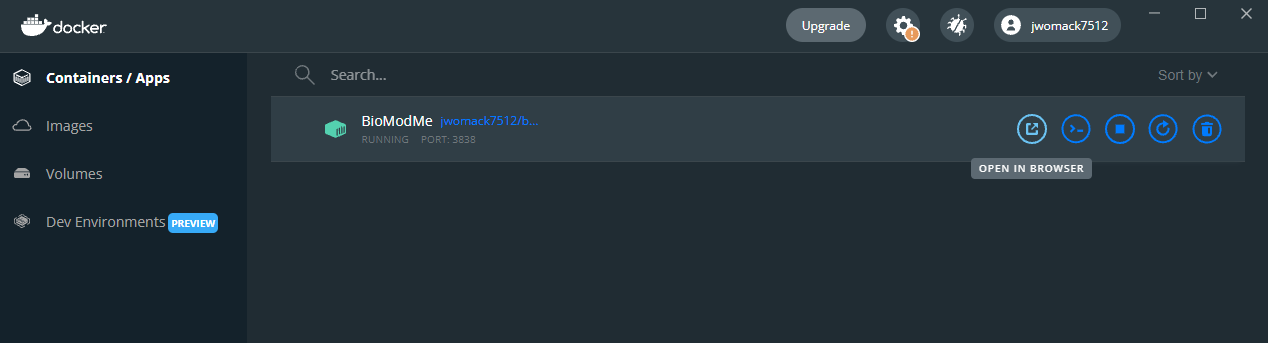


In the above figure, if you double click the container the logs will open for the application. These can be useful for debugging and submitting errors.

# Model Execution

This section will cover the **Execute Model** tab and other methods of model execution. The starting page layout is as follows (after loading the basic tutorial in):


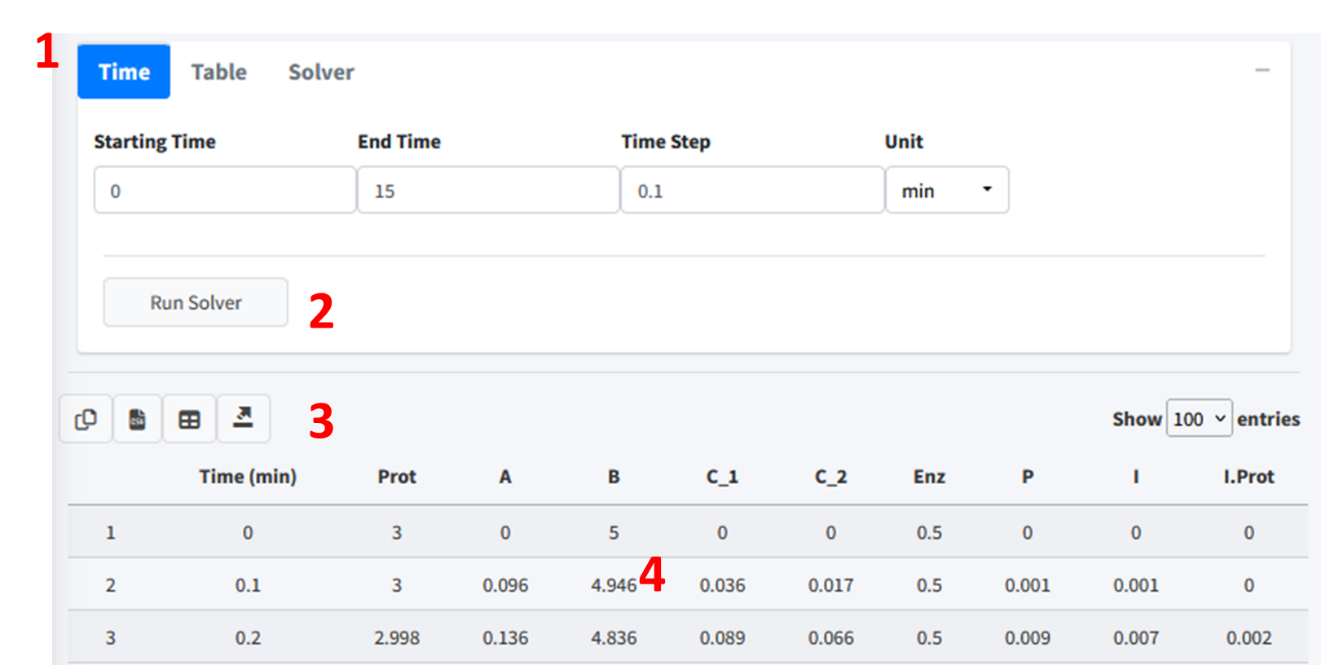


(1) Tab box containing different options for model solving.

(i) Time – Set the time for the model solver.

(ii) Table – Contains viewing options on how to show the bottom output table.

(iii) Solver – Set different solver options for the model execution.

(2) Button that will execute the model for solving.

(3) Export buttons for the result table (copy, csv, excel, popout window)

(4) Result table showing the time step solution of the ODE-based model.

## Execution in Visualization Tab:

For simplicity, we have added the ability to directly change the model in the **Visualization** tab. At the top right corner of the plot, there is a button labeled “Resolve Model”.


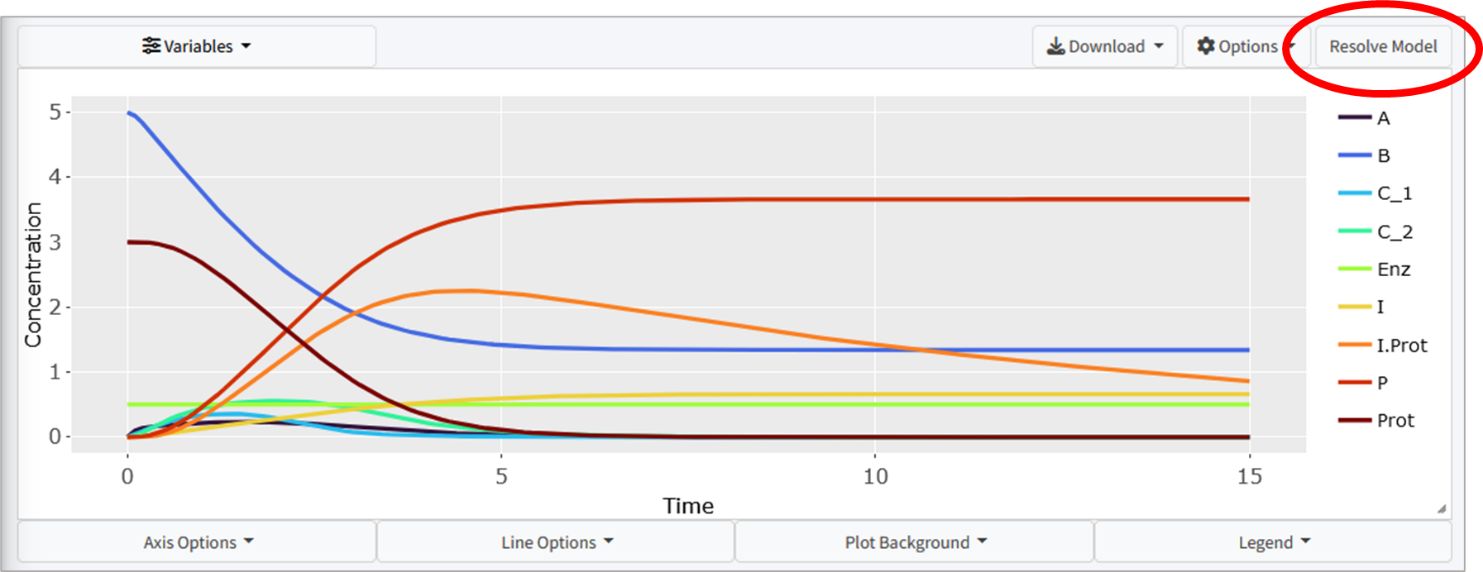


This will resolve the model, according to the specifications in the **Execute Model** tab without the need to switch tabs. To accompany this, below the plot a box has been added that allows the user to directly change model variable values:


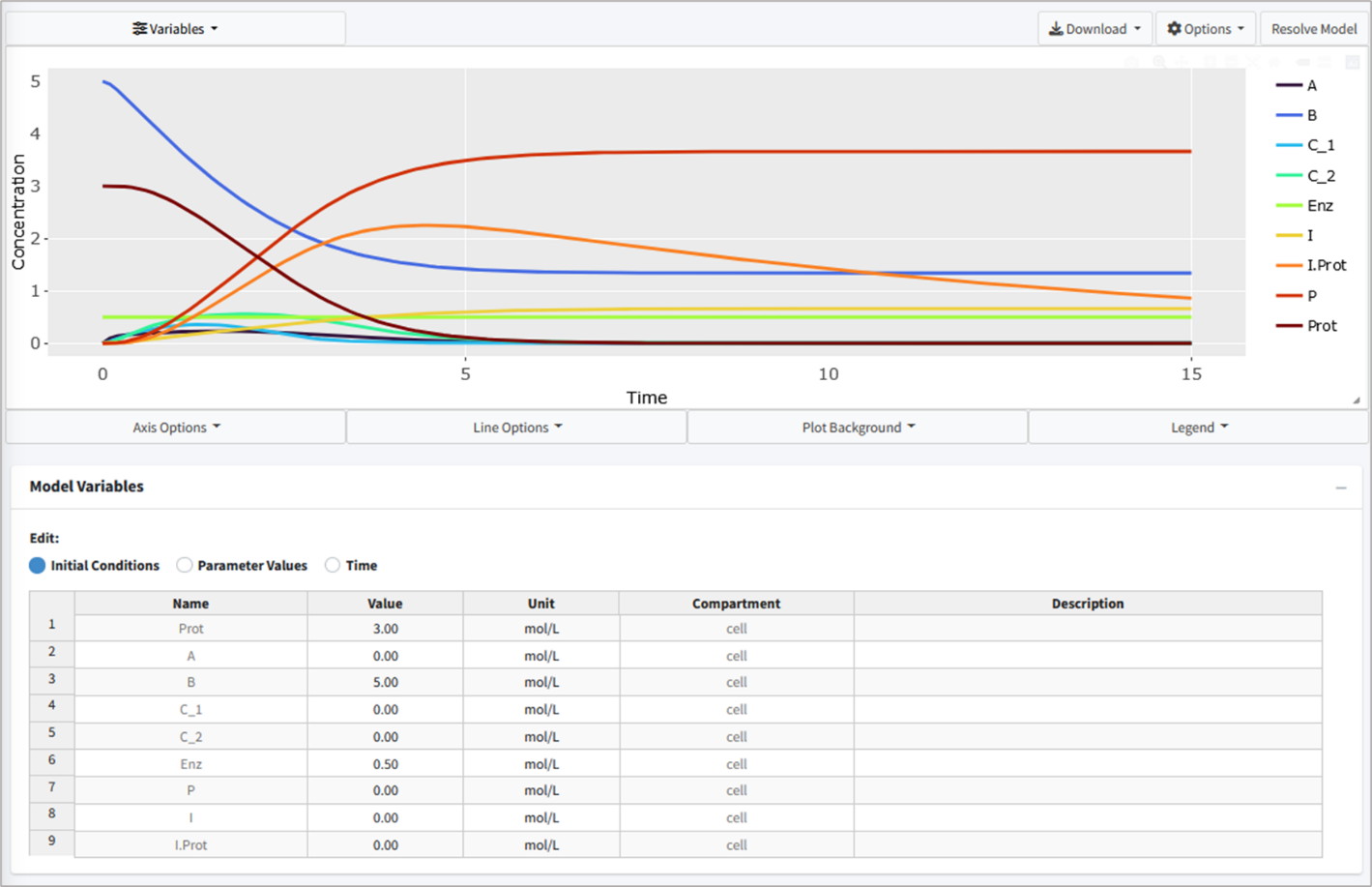


In this way, the user can test different model parameters and solve the model all without needing to switch between multiple tabs. Model changes on this section will change the values everywhere.

# Mathematical Derivations

This section covers all the mathematical equations and derivations that run in the background of BioModME.

## Equations

This section contains the mathematical derivations used for the equation builders found in this program.

### Chemical Based Equations

-Law of Mass Action

Chemical reactions are derived using the law of mass action. Given the following chemical reaction scheme:


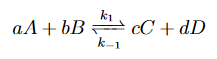


where a, b, c, d are stoichiometric coefficients and A, B, C, D are the chemical species. The law is as follows:

$$-\frac{1}{a}\frac{d\left[ A \right]}{\mathrm{dt}}=-\frac{1}{b}\frac{d\left[ B \right]}{\mathrm{dt}}=\frac{1}{c}\frac{d\left[ C \right]}{\mathrm{dt}}=\frac{1}{d}\frac{d\left[ D \right]}{\mathrm{dt}}=k_{1}\left[ A \right]^{a}\left[ B \right]^{b}-k_{-1}\left[ C \right]^{c}\left[ D \right]^{d}$$

Where [X] denotes concentration of X. From the above law, each individual term can is derived below:

$$\frac{d\left[ A \right]}{dt}=-a*k_{1}\left[ A \right]^{a}\left[ B \right]^{b}+a*k_{-1}\left[ C \right]^{c}\left[ D \right]^{d}$$

$$\frac{d\left[ B \right]}{\mathrm{dt}}=-b*k_{1}\left[ A \right]^{a}\left[ B \right]^{b}+b*k_{-1}\left[ C \right]^{c}\left[ D \right]^{d}$$

$$\frac{d\left[ C \right]}{\mathrm{dt}}=c*k_{1}\left[ A \right]^{a}\left[ B \right]^{b}-c*k_{-1}\left[ C \right]^{c}\left[ D \right]^{d}$$

$$\frac{d\left[ D \right]}{\mathrm{dt}}=d*k_{1}\left[ A \right]^{a}\left[ B \right]^{b}-d*k_{-1}\left[ C \right]^{c}\left[ D \right]^{d}$$

Each individual equation will be added to their overall flux differential equation in the differential equation solver section.

-Law of Mass Action with Regulation

Mass action with regulation is for chemical reactions who have rate constants that are dependent on other species. An example is the regulation of inactive MPF to active MPF in the cell cycle by WEE1 and CDC25Cp. In this example, inactive MPF is phosphorylated by CDC25Cp and dephosphorylated by WEE1. These regulators are not directly affected in the differential equations in this situation while inactive and active MPF are. We have the same derivation as mass action except the rate constants are changed to reflect the regulation.


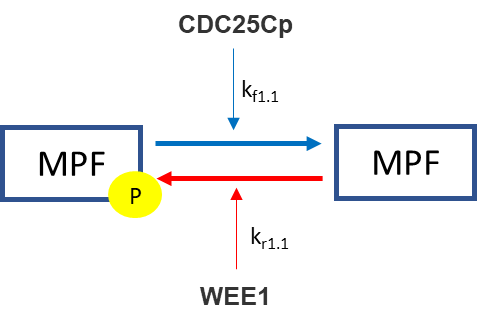


The regulators will take the respective place of the rate constants in the mass action derivation. In the mass action derivation above for [A] and [B] we have:

$$\frac{d\left[ A \right]}{dt}=-a*k_{1}\left[ A \right]^{a}\left[ B \right]^{b}+a*k_{-1}\left[ C \right]^{c}\left[ D \right]^{d}$$

$$\frac{d\left[ B \right]}{dt}=-b*k_{1}\left[ A \right]^{a}\left[ B \right]^{b}+b*k_{-1}\left[ C \right]^{c}\left[ D \right]^{d}$$

Adding regulator replaces the respective rate constant with the sum of its regulators and rate constants:

$$\frac{d\left[ A \right]}{dt}=-a*\left( \sum kf_{i}*\left[ reg_{i} \right] \right)\left[ A \right]^{a}\left[ B \right]^{b}+a*\left( \sum kr_{i}*\left[ reg_{i} \right] \right)\left[ C \right]^{c}\left[ D \right]^{d}$$

$$\frac{d\left[ B \right]}{dt}=-b*\left( \sum kf_{i}*\left[ reg_{i} \right] \right)\left[ A \right]^{a}\left[ B \right]^{b}+b*\left( \sum kr_{i}*\left[ reg_{i} \right] \right)\left[ C \right]^{c}\left[ D \right]^{d}$$

Using the above example (which is one reactant/product), we get the following result:

$$\frac{d\left[ MPFp \right]}{dt}=- \left( k_{f1.1}*[CDC25Cp] \right)*\left[ MPF \right]+\left( k_{r1.1}*\left[ WEE1 \right] \right)*[MPFp]$$

$$\frac{d\left[ MPF \right]}{dt}=\left( k_{f1.1}*\left[ CDC25Cp \right] \right)*\left[ MPF \right]-\left( k_{r1.1}*\left[ WEE1 \right] \right)*[MPFp]$$

##### -Michaelis Menten


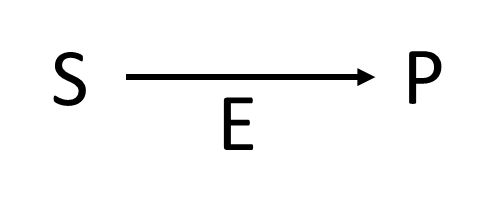
Michaelis Menten kinetics is a commonly used approximation for describing the rate at which enzymatic reactions occur. A single enzymatic reaction can be described by:

In the above, S is the substrate, E is the enzyme, and P is the product of the reactions. The model most commonly takes the following for describing the velocity of a reaction:

$$v=\frac{d\left[ P \right]}{dt}=V_{max}\frac{\left[ S \right]}{K_{M}+\left[ S \right]}=\left( k_{cat}*\left[ E \right] \right)\frac{\left[ S \right]}{K_{M}+\left[ S \right]}$$

where,

**Vmax -** Maximum Velocity

**E -** Enzyme Concentration

**S -** Substrate Concentration

**P -** Product Concentration

**K_M_ -** Michaelis Menten Constant

**k_cat_ -** Catalytic Rate of Enzyme Reaction

-Synthesis

Synthesis by rate has a species generated at a specific rate. This is useful for when the user does not particularly know what is causing the synthesis but knows how quickly it is being produced. It simply creates a constant rate of production.

$$\frac{d\left[ \mathrm{Species} \right]}{\mathrm{dt}}=k_{\mathrm{syn}}$$

where,

**k_syn_ -** synthesis rate of species

-Synthesis by Regulation

The “By Factor” law is used when a regulator directly influences the synthesis of a species but is not directly converted. An example of this is in the Cell Cycle when the molecule E2F activates transcription factors that synthesize Cyclin E and Cyclin A. If the cell contains no E2F, then there is no synthesis of Cyclin E or Cyclin A.


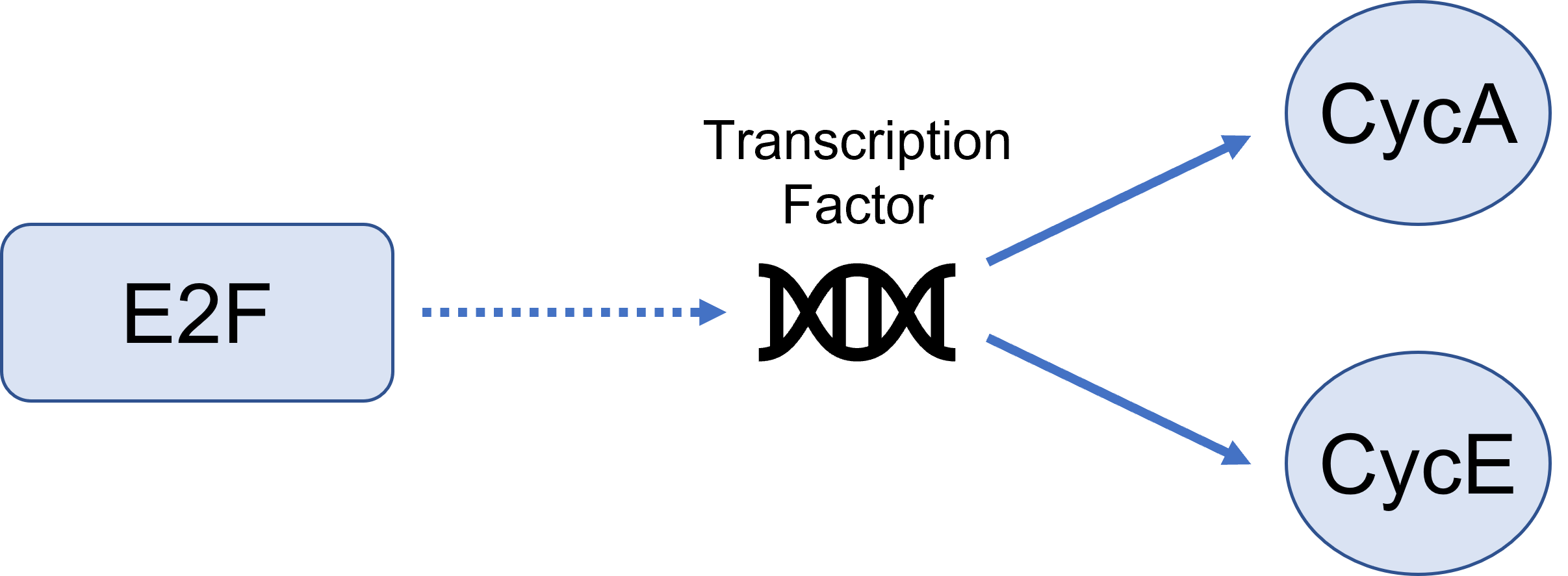


There is only one resultant mathematical flux from this type of synthesis and that is the species being synthesized. The factor causing the synthesis does not have its flux altered. The general resulting equations are:

$$\frac{d\left[ Species \right]}{dt}=k_{syn}*\left[ Factor \right]$$

$$\frac{\left[ Factor \right]}{dt}=0$$

where,

**k_syn_ -** synthesis rate of species

**Species -** species that is being synthesized

**Factor -** factor that is causing the synthesis

In relation to the above E2F equation, the differential equations are:

$$\frac{d\left[ CycA \right]}{dt}=k_{syn}*\left[ E2F \right]$$

$$\frac{d\left[ CycE \right]}{dt}=k_{syn}*\left[ E2F \right]$$

$$\frac{d\left[ E2F \right]}{dt}=0$$

-Degradation

These reactions are dependent on a rate constant. Here we have two specific options where the degradation can be concentration dependent or not. Often degradations are concentration dependent on themselves, meaning there is more degradation at higher concentrations and vice versa. The following is the derivation for a concentration dependent degradation:

$$\frac{d\left[ Species \right]}{dt}=-k_{deg}*\left[ Species \right]$$

where,

**k_deg_ -** degradation rate constant

**Species -** species being degraded

When the degradation is not concentration dependent, the resulting flux is:

$$\frac{d\left[ Species \right]}{dt}=-k_{deg}$$

When products are made, they are the opposite sign of the above reactions. For example, for concentration dependent reactions with one degradation to two products we would have the resulting differential equations:

$$\frac{d\left[ Species \right]}{dt}=-k_{deg}*\left[ Species \right]$$

$$\frac{d\left[ Product1 \right]}{dt}=+k_{deg}*\left[ Species \right]$$

$$\frac{d\left[ Product2 \right]}{dt}=+k_{deg}*\left[ Species \right]$$

-Degradation by Enzyme

This degradation option is a basic enzyme reaction using Michaelis Menten kinetics where the substrate is the species to be degraded and there often is no product. The resulting mathematical flux for the degraded substrate would be:

$$\frac{d\left[ S \right]}{dt}=-V_{max}\frac{\left[ S \right]}{K_{m}+\left[ S \right]}=-\left( k_{cat}*\left[ E \right] \right)\frac{\left[ S \right]}{K_{m}+\left[ S \right]}$$

where,

**V_max_ -** Maximum Velocity

**E -** Enzyme Concentration

**S -** Substrate Concentration

**P -** Product Concentration

**K_M_ -** Michaelis Menten Constant

**k_cat -_** Catalytic Rate of Enzyme Reaction

There is an option to add a product (P), which results in the mathematical flux equations to break down to pure Michaelis Menten kinetics for the product and substrate which would have the same equation above except with a positive sign:

$$\frac{d\left[ S \right]}{dt}=-V_{max}\frac{\left[ S \right]}{K_{m}+\left[ S \right]}=-\left( k_{cat}*\left[ E \right] \right)\frac{\left[ S \right]}{K_{m}+\left[ S \right]}$$

$\frac{d\left[ P \right]}{dt}=-V_{max}\frac{\left[ S \right]}{K_{m}+\left[ S \right]}=-\left( k_{cat}*\left[ E \right] \right)\frac{\left[ S \right]}{K_{m}+\left[ S \right]}$

### Input/Output

This section contains the mathematical derivations used for the input/output builders found in this program.

#### -Flow Between

Flow between two or more compartments follow the mechanics described below. In the below figure, we have flow between two compartments. We have species A_1_ flowing out of compartment 1 into compartment 2 as A_2_.


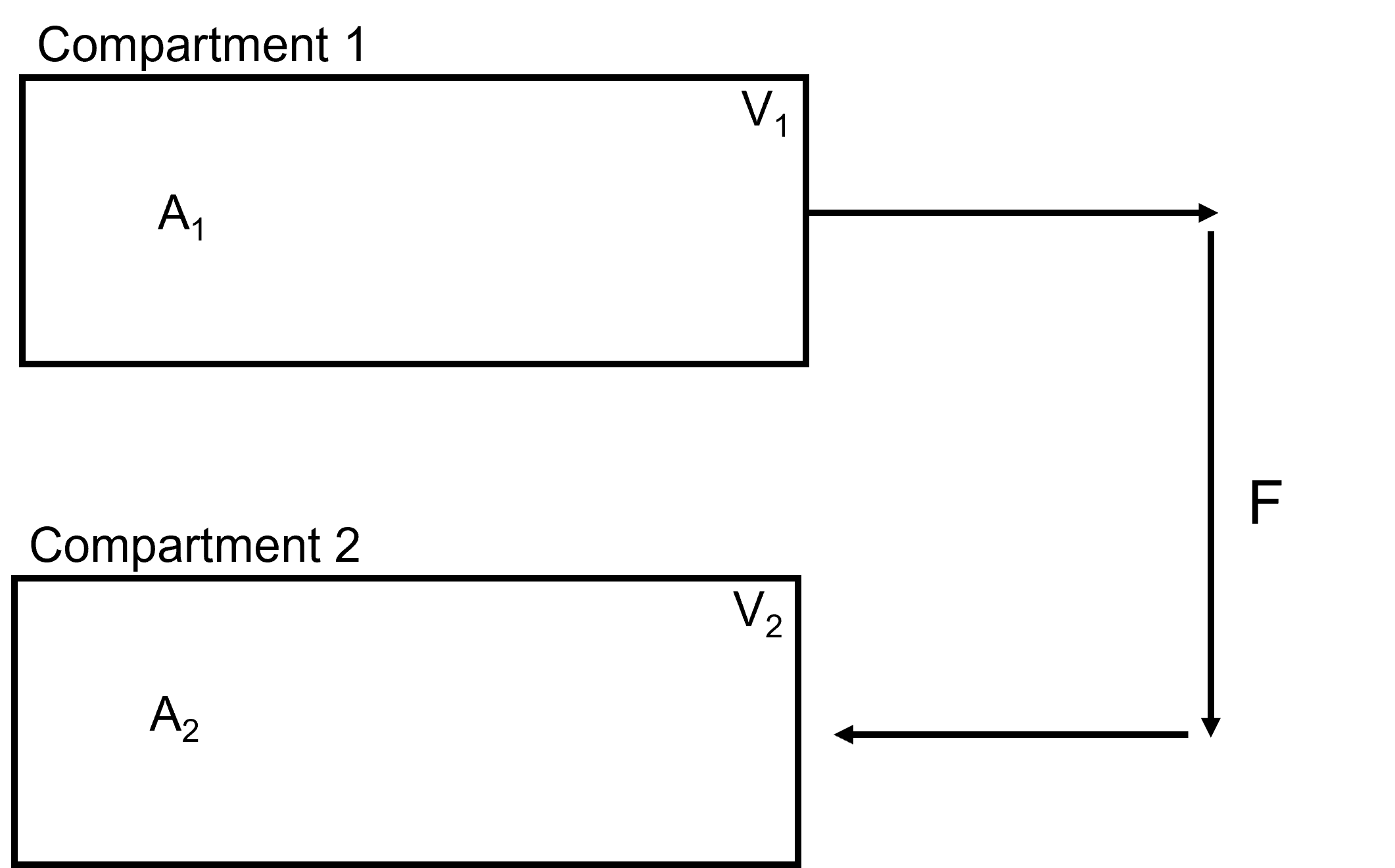


The flows from a compartment are derived as the flow rate (F) multiplied by the concentration of the species leaving the compartment as a minus term. Flow to a compartment is the same derivation without the minus term. The flows in the above diagram would be derived as:

$$V_{1}*\frac{d\left[ A_{1} \right]}{dt}=-F*{[A}_{1}]$$

$$V_{2}*\frac{d\left[ A_{2} \right]}{dt}=F*{[A}_{1}]$$

##### -Split Flow

Often, we need to split the flow from one compartment to multiple compartments. This option splits the flow from one compartment to go to multiple compartments. In the figure below, we see the flow from compartment 1 is split to go to compartment 2 and compartment 3.


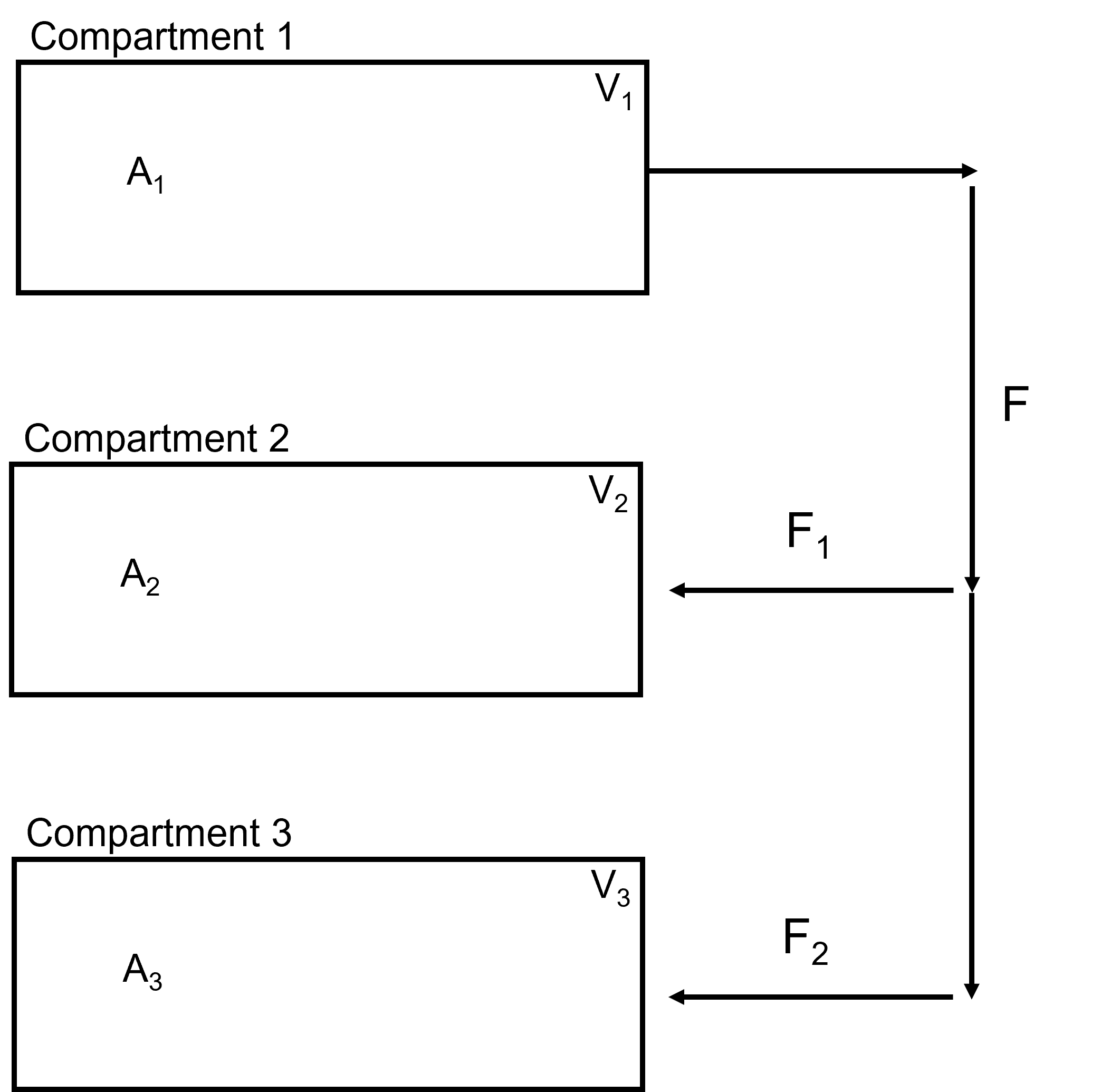


The summation of the output flows will be equal to the input flow:

$$F_{out}=\sum_{1}^{n} F_{in}$$

Given the above figure this equation derives out to:

$$F=F_{1}+F_{2}$$

The resulting flow differential equations derive as:

$$V_{1}\frac{d{[A}_{1}]}{dt}=-F*{[A}_{1}]$$

$$V_{2}\frac{d{[A}_{2}]}{dt}=-F_{1}*{[A}_{1}]$$

$$V_{3}\frac{d{[A}_{3}]}{dt}=-F_{2}*{[A}_{1}]$$

#### -Flow In

Constant flow into a compartment from an unspecified source. This could be something like an input flow into a model of species or a known constant flow from a source outside the scope of your created model. Flow In and Flow Out options can also be pieced together to create a customized Flow Between. In the Figure below, we see a constant flow, **F**, carrying species, **A1**, into Compartment 1.


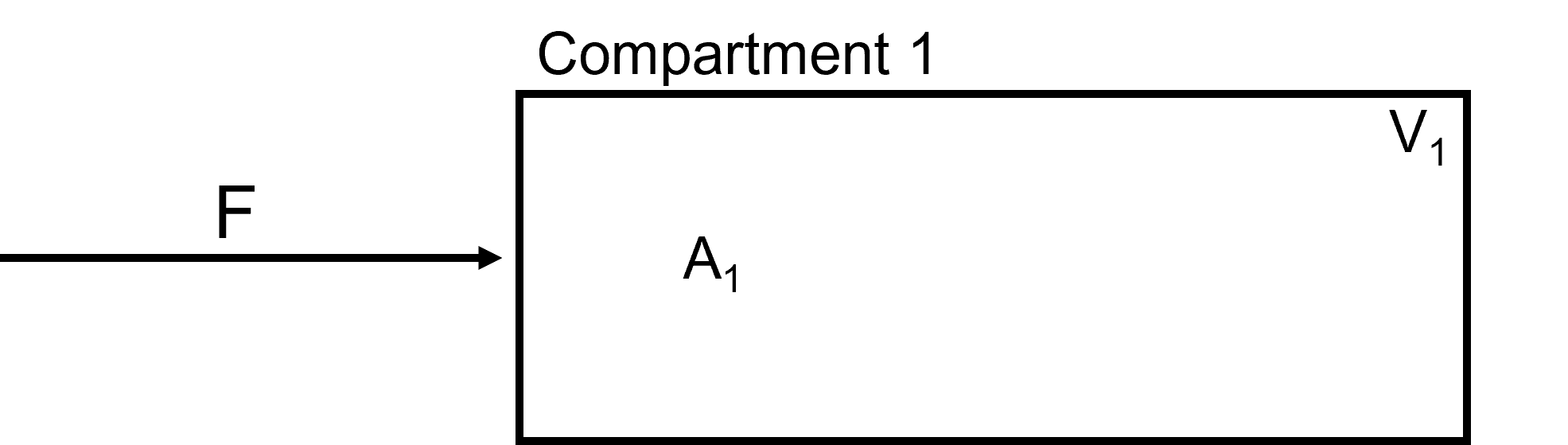


The mathematical derivation for this model input would be:

$$V_{1}\frac{d[A_{1}]}{dt}=F*[A_{1}]$$

#### -Flow Out

Constant flow out of a compartment that does not go to another compartment. Flow In and Flow Out options can be pieced together to create a customized Flow Between. In the Figure below, we see a constant flow, **F**, carrying species, **A_1**, out of Compartment 1.


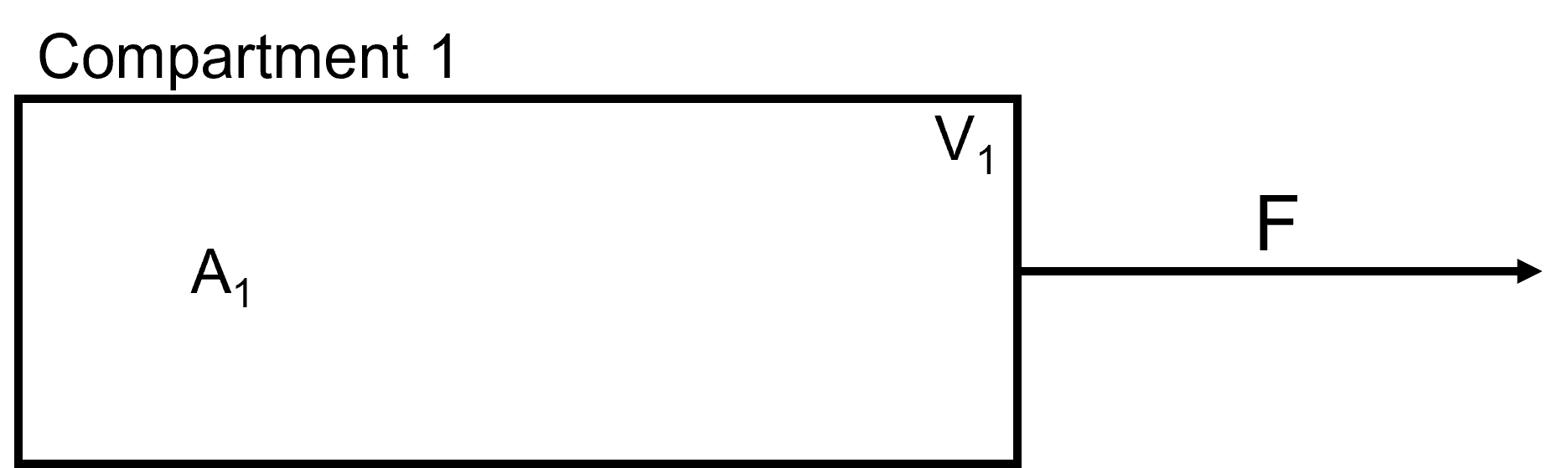


The mathematical derivation for this model output would be:

$$V_{1}\frac{d[A_{1}]}{dt}=-F*{[A}_{1}]$$

#### -Clearance

Clearance from a compartment is used when a species leaves the compartment and is removed from the model. In the example below, we have species A_3_ being cleared from compartment 3.


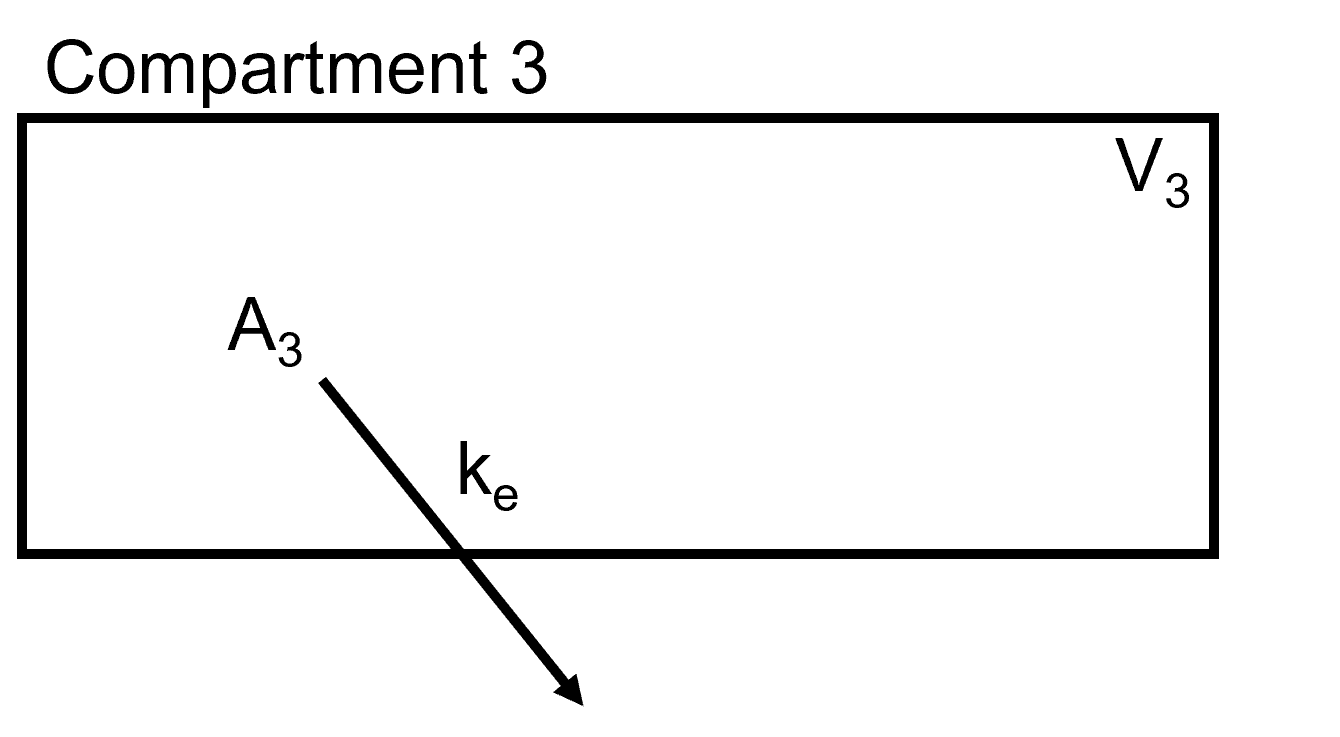


It takes the following derivation:

$$V_{3}\frac{d{[A}_{3}]}{dt}=-k_{e}*{[A}_{3}]*V_{3}$$

where,

k_e_ - Rate of clearance

A_3_ - Concentration of Species being cleared

V_3_ - Volume of compartment that species is being removed from

#### -Simple Diffusion

In simple diffusion, the substance is driven by concentration difference across a membrane. In the example below, we have a liver compartment that is separated into blood and liver compartments with a passive diffusion membrane for compound **A**.


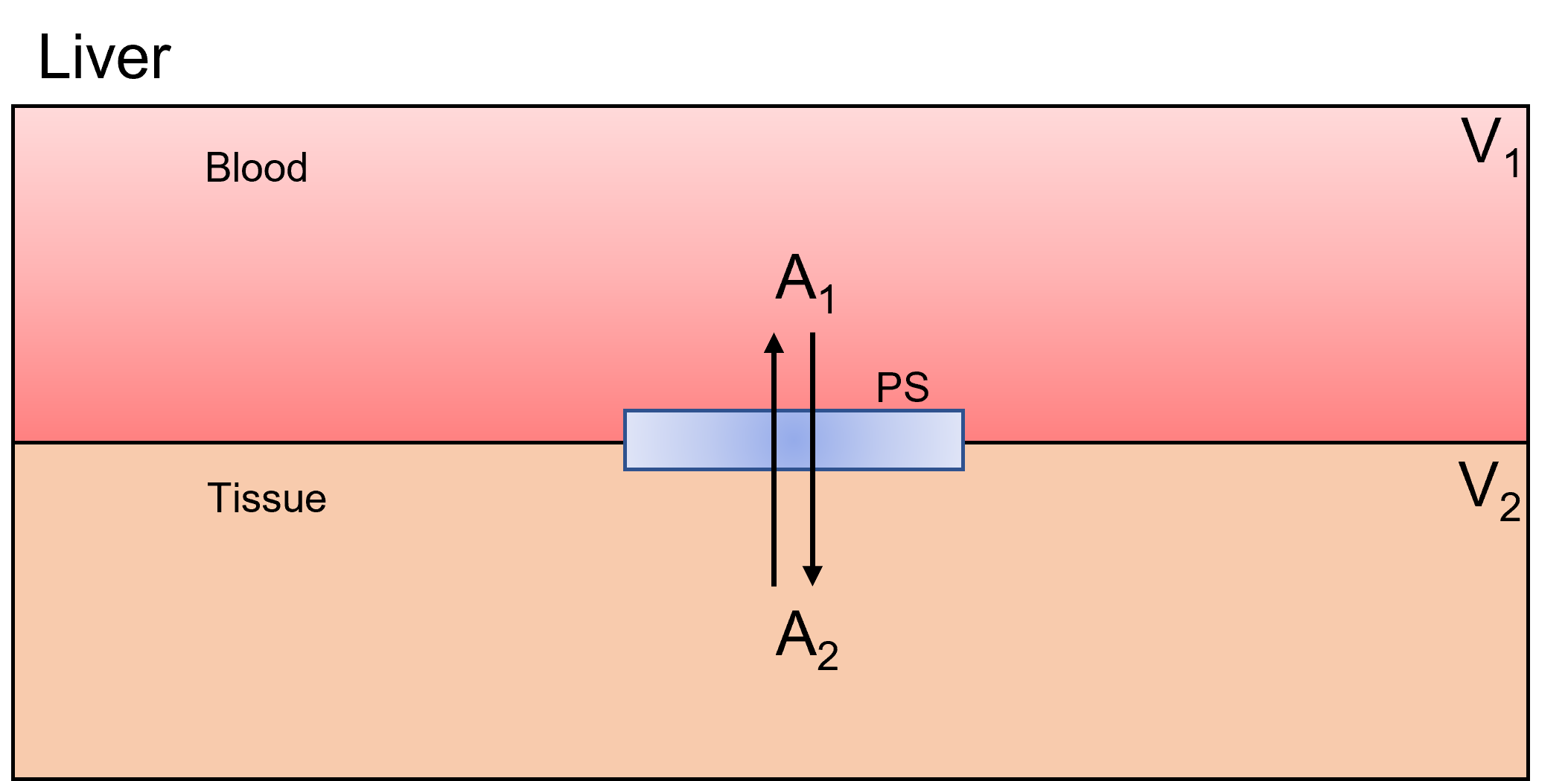


For the simple, one-dimensional case shown above, the mass flux through the membrane follows Ficks Law:

$$J=-D\frac{dC}{dx}$$

where,

C - Concentration at membrane location x

D - Diffusion coefficient of the molecule

We assume the concentration gradient across the membrane of thickness, x, falls linearly with x leading to:

$$\frac{dC}{dx}=\frac{\Delta C}{\Delta X}=\frac{C_{2}-C_{1}}{\Delta x}$$

$$J=-D\frac{\Delta C}{\Delta x}= -D\frac{C_{2}-C_{1}}{\Delta x}$$

The rate of transfer (**f**) of a neutral compound across a biological membrane of surface area (**S**) is:

$$f=JS=-SD\frac{\Delta C}{\Delta x}=-PS\left( C_{2}-C_{1} \right)$$

where,

PS – Permeability surface area product parameter

Note: The **negative sign** on the right side of the equation indicates that the net transfer due to diffusion is in a direction away from the region with higher concentration.

-Facilitated Diffusion

Facilitated diffusion is a type of diffusion in which the molecules move from the regions of higher concentration to the region of lower concentration assisted by a carrier molecule. It is a selective process, only allowing specific molecules to pass the gradient. In the below figure, we have a liver compartment that is separated into blood and liver compartments with a facilitated transporter for compound **A**. Note that this is a one-way transport.


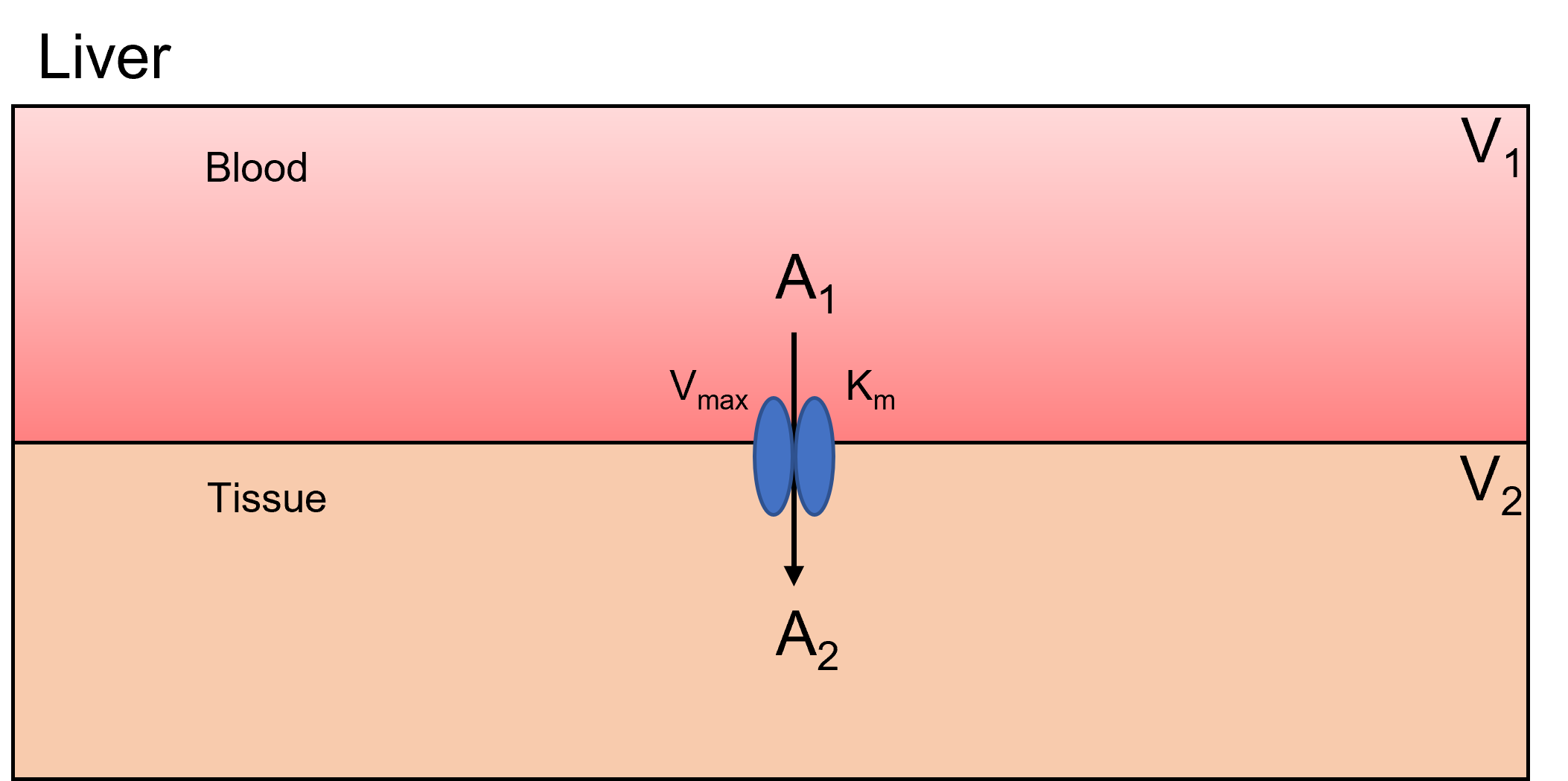


For the derivation of the flux equation for facilitated diffusion, we take the following equation:

$$F=\frac{V_{max}*\left[ A_{1} \right]}{K_{m}+[A_{1}]}$$

where,

V_max_ - Maximum Velocity

$\boldsymbol{A}_{\boldsymbol{1}}$ - Substrate Concentration

K_m_ - Affinity of how much substrate concentration needed to reach V_max_

The flux derivations for the above example would be:

$$V_{1}\frac{d{[A}_{1}]}{dt}=-\frac{V_{max}*{[A}_{1]}}{k_{m}+{[A}_{1}]}$$

$$V_{2}\frac{d{[A}_{2}]}{dt}=\frac{V_{max}*{[A}_{1}]}{k_{m}+{[A}_{1}]}$$

Units

BioModME supports basic units for species and parameters in a model. The unit definition system is created from the R package “measurements” (<https://cran.r-project.org/web/packages/measurements/measurements.pdf>). This package supports several commonly found units. The user can define the units how they want and the application will convert them to a base unit in the backend for processing.

Unit Conversion

To convert units, go to the units section of the data table for the variable. For example, we will look at a parameter table example. The third column in the table is units. By clicking the units square, we can change the unit type.


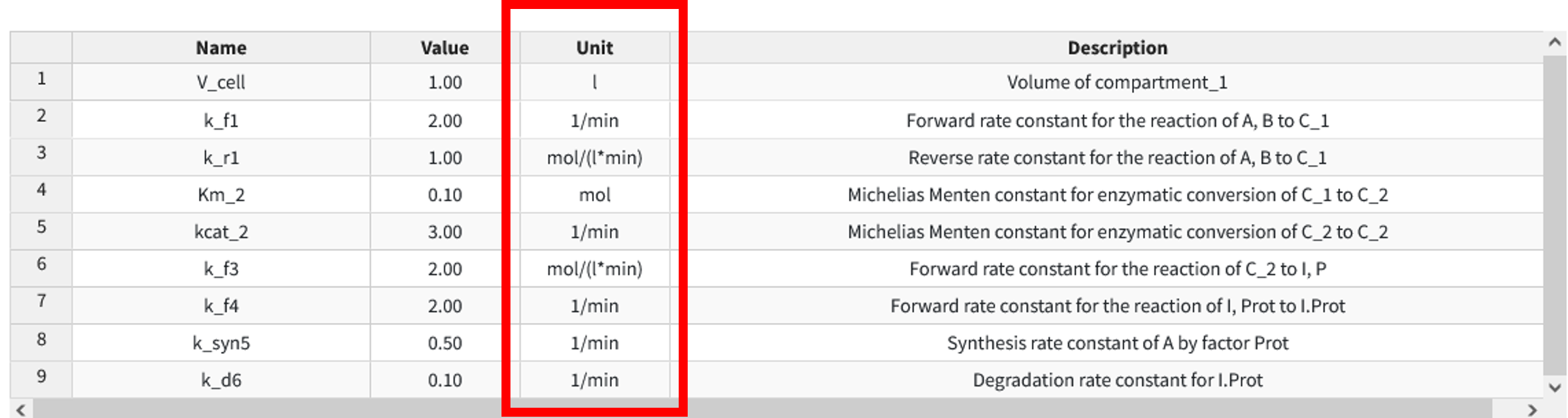


The program will only allow you to convert to accepted units for that type. Meaning, for example, V_cell has the units of liter, so it can only be changed to another unit of volume (such as mL). For a list of all units and their values, see the unit definition section. If you attempt to convert to a unit that is not allowed, a warning will appear.


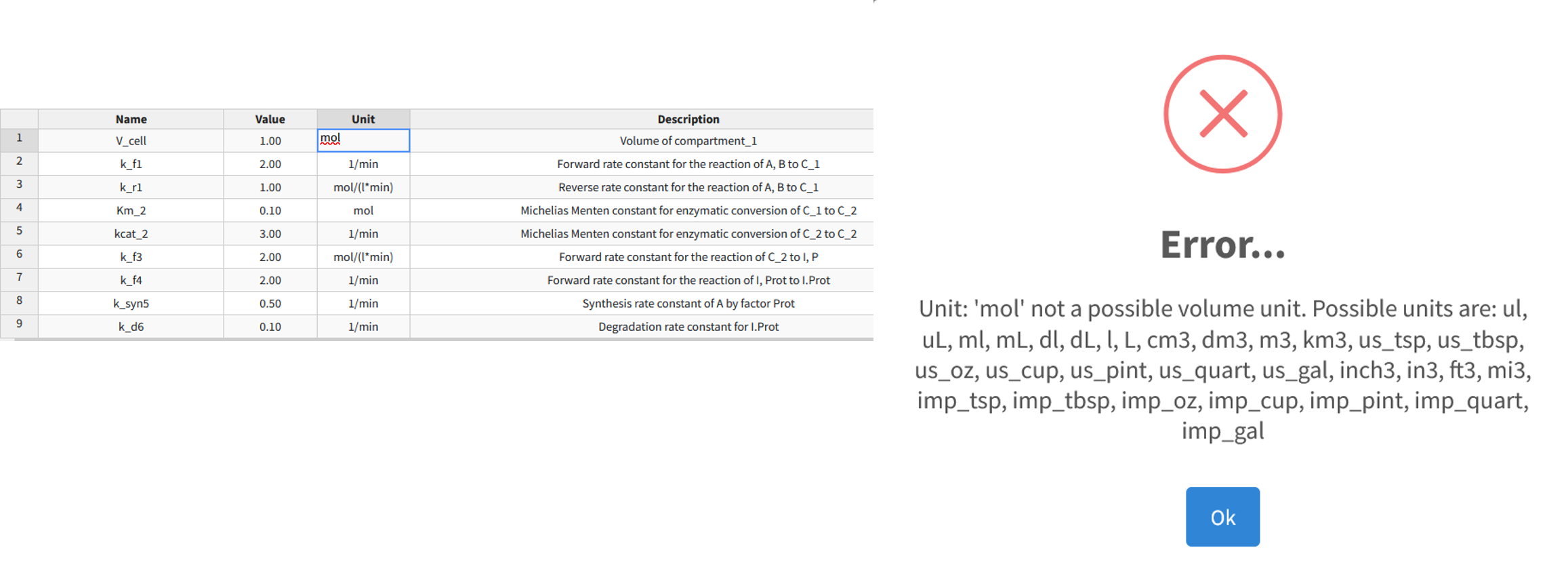


Converting Compound Units

Often parameter values will have multiple unit definitions. From the parameter table example above, the parameter “k_r1” has a unit definition of mol/(l*min). This has units of count, volume, and time. To convert this, the user needs to ensure they repeat the whole expression changing the appropriate unit in the proper place. For example,

$$mol/(l*min)$$

is an appropriate expression. However,

$$umol/(min*l)$$

will return an error (POSITION MATTERS). As well as not putting the mathematical notion back in its correct place (the division, multiplication, and parenthesis) or having any of the units not be the correct conversion type (i.e. using volume units when you should be using temp).

Unit Definitions

Below are the unit definitions used in this application. The left-hand side is the term that can be put into the application (parenthesis terms are alternat acceptable names) while the right side is the definition of the unit abbreviation.

**Count**

**fmol:**

femto-mol

**pmol:**

pico-mol

**nmol:**

nano-mol

**umol:**

micro-mol

**mmol:**

milli-mol

**mol:**

mol

**Flow**

**ml_per_sec:**

milliliter per second

**ml_per_min:**

milliliter per min

**ml_per_hr:**

milliliter per hour

**l_per_sec (LPS):**

liter per second

**l_per_min (LPM):**

liter per min

**l_per_hr (LPH):**

liter per hour

**Temperature:**

**C:**

Celsius

**F:**

Fahrenheit

**K:**

Kelvin

**R:**

Rankine

**Time:**

**ns:**

nano-second

**us:**

micro-second

**ms:**

milli-second

**s:**

second

**min:**

minute

**hr:**

hour

**day:**

day

**wk:**

week

**mon:**

month

**yr:**

year

**dec:**

decade

**cen:**

century

**Volume:**

**ul (uL):**

micro-Liter

**ml (mL):**

milli-Liter

**dl (dL):**

deci-Liter

**l (L):**

Liter

**us_tsp:**

US teaspoon

**us_tbsp:**

US tablespoon

**us_oz:**

US ounce

**us_cup:**

US cup

**us_pint:**

US pint

**us_quart:**

US quart

**us_gal:**

US gallon

**cm3:**

centimeter cubed

**dm3:**

decimeter cubed

**m3:**

meter cubed

**km3:**

kilometer cubed

**in3:**

inch cubed

**ft3:**

feet cubed

The package we use for unit definitions “measurements” has many redundant units. For example, ns and nsec both mean nano-second. In an attempt to reduce the number of units and standardize unit definitions, we have changed the following units in the application:


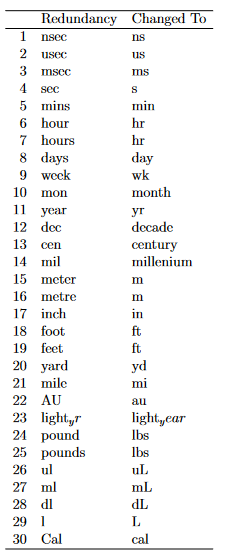


These units can still be used in the units when typing them in, but will be changed to the second column in the application.

Pre-generated units for rate constants:

| Law | Rate Constant | Unit Definition | Example |
| --- | --- | --- | --- |
| Mass Action | Forward rate constant, kf | Order Dependent; See Below | 1/min, L/(mol*min) |
| Mass Action | Reverse rate constant, kr | Order Dependent; See Below | 1/min, L/(mol*min) |
| Synthesis, Rate | Ks | Count/(Volume*Time) | mol/(L*min) |
| Synthesis, uses Modifier | Ks | 1/Time | 1/min |
| Degradation, Rate | Kd | 1/time | 1/min |
| Degradation, by enzyme | Km | count/volume | mol/L |
| Degradation, by enzyme | kcat | 1/time | 1/min |
| Degradation, by enzyme | Vmax | count/(volume*time) | mol/(L*min) |
| Michaelis-Menten | Km | count/volume | mol/L |
| Michaelis-Menten | kcat | 1/time | 1/min |
| Michaelis-Menten | Vmax | count/(volume*time) | mol/(L*min) |

For Order dependent constants (law of mass action) the rate constant has units of

$$\frac{count^{1-n}}{volume^{1-n}*time}$$

where n is the order of the reaction. This gives the following:

For order zero (n = 0),

$$\frac{count}{volume*time}\left[ = \right]\frac{mol}{L*min}$$

For order one (n = 1),

$$\frac{1}{time}\left[ = \right]\frac{1}{min}$$

For order two (n = 2),

$$\frac{volume}{count*time}\left[ = \right]\frac{L}{mol*min}$$

For order three (n = 3),

$$\frac{volume^{2}}{count^{2}*time}\left[ = \right]\frac{L^{2}}{mol^{2}*min}$$

And so on for each increasing order of n. The order of the reaction is determined by looking at the rate law and is the sum of the exponents of the species multiplied by the constant. For example, the reaction that has the forward rate constant kf:

$$A + 2B \to C$$

Has the rate of,

$$v = kf{[A]}^{1}{[B]}^{2}$$

this rate constant, kf, is thus of order 3 (1 + 2).

# Parameter Estimation

Our application uses the minpack.lm library (https://cran.r-project.org/web/packages/minpack.lm/index.html) in R to perform parameter estimation. This package provides methods for solving nonlinear least-squares problems by a modification of the Levenberg-Marquardt algorithm, with support for lower and upper parameter bounds.

### 1. Non-Linear Least Squares:

In simple terms, a least squares problem aims to minimize the sum of the squared residuals (or errors). In mathematical terms, given a function f(x,p), where x is the vector of data points and p is a vector of parameters, the objective is to find the vector p that minimizes:

$$\sum_{i} \left( y_{i}-f\left( x_{i},p \right) \right)^{2}$$

where y_i​_ are the observed values.

The term "non-linear" indicates that the relationship between the parameters p and the data x is non-linear.

### 2. Levenberg-Marquardt Algorithm:

The Levenberg-Marquardt algorithm is a popular iterative method for solving non-linear least squares problems. It is essentially a combination of the gradient descent method and the Gauss-Newton method. The algorithm updates the parameter vector pp in each iteration using the formula:

$$\boldsymbol{p}_{new}=\boldsymbol{p}_{old}+\left( J^{T}J+\lambda I \right)^{-1}J^{T}r$$

Where:

- J is the Jacobian matrix of the residuals with respect to the parameters.
- r is the vector of residuals.
- λ is a damping factor.
- I is the identity matrix.

The damping factor λ is adjusted dynamically during the iterations. If a trial step results in a reduction of the residual sum of squares, λ is decreased (making the update more like Gauss-Newton), and if the trial step increases the residual sum of squares, λ is increased (making the update more like gradient descent).

# Creating A Custom Law

This will cover all the necessary components of creating new laws. BioModME is still a relatively new program and, as such, consists of a small number of premade laws. In this section, we will go over how to create and use a custom law in BioModME. To start, navigate to “Modeler’s Toolbox > Build Custom Law”. You should end up on a page that has the below box:


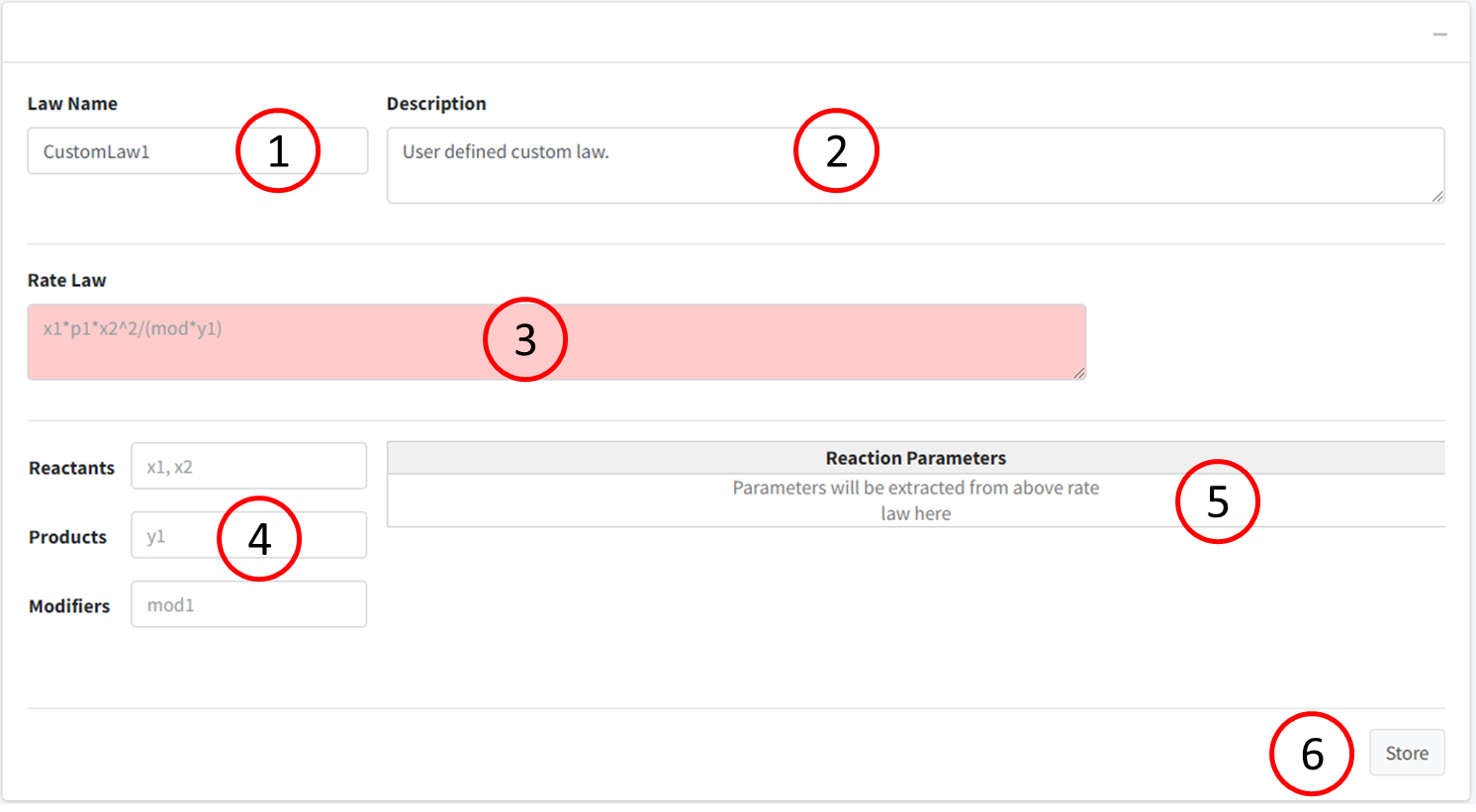


In the figure above, we have the following:

1. Law Name – Enter the name to identify law by
2. Description – Description of law
3. Rate Law – Box to enter string version of the equation (red when not valid, green when valid)
4. Boxes to tell the program which variables in the model are reactants, products, or modifiers. Any variables listed in **Rate Law** are auto assigned as a parameter. Typing that value into one of the respective three boxes will remove it from the parameter tag.
   1. Reactant – species on left hand side of equation and adopt a negative version of rate law.
   2. Products – species on the right-hand side of equation that adopt a positive version of the rate law.
   3. Modifiers – species that are involved in the reaction but do not have a rate law from the reaction.
5. Table of parameters extracted from **Rate Law**. Here there will be customization options.
6. Button to press to store equation.


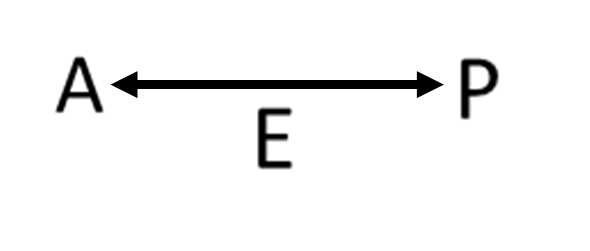
Example : Reversible Michaelis-Menten Reaction

Given a reversible enzyme reaction given by:

Where A is the reactant, P is the product, and E is the enzyme. We derive the following rate law:

$$\frac{V_{f}*\frac{\left[ A \right]}{{Km}_{A}}-V_{r}*\frac{\left[ P \right]}{{Km}_{P}}}{1+ \frac{\left[ A \right]}{{Km}_{A}}+\frac{\left[ P \right]}{{Km}_{P}}}$$

Where:

- V_f_ – forward velocity (parameter)
- V_r­_ - reverse velocity (parameter)
- Km_A_ – Michaelis-Menten Constant of A (parameter)
- Km_P_ – Michaelis-Menten Constant of P (parameter)
- A – concentration of species A (reactant)
- P – concentration of species P (product)
- E – concentration of species E (modifier)

Note that even though the enzyme is not in the rate law, it should still be included in the equation description.

Below is how the form should look for entering this law:


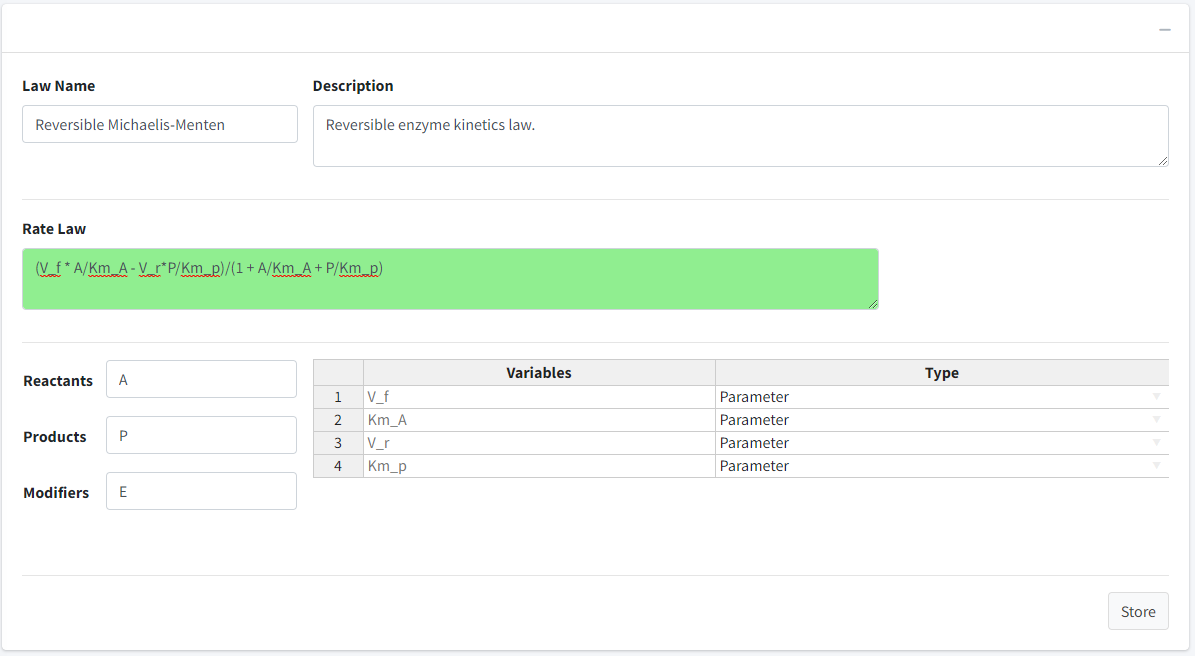


Steps:

1. Name the law: Reversible Michaelis-Menten
2. Add a description!
3. Type a string version of the rate law. We have built a parser that determines if the law is valid. The **Rate Law** box will be red if the equation is not valid and green if valid.
4. Put A in reactants, P in Products, and E in Modifiers.
5. Press the “Store” Button. A success message should appear.

Important things to note here, when creating your own law, all parameter values will be dimensionless. The parameter table provides a dropdown on “Type” if the parameters are a volume. If time, we ask you to use “t” as the variable.

To use the newly created law, travel to “Create Model >Reactions Box > +” to add a new reaction. In the **Reaction Type** input, select “Custom Reaction”. In **Reaction Law**, there should be the newly created law “Reversible Michaelis-Menten”.


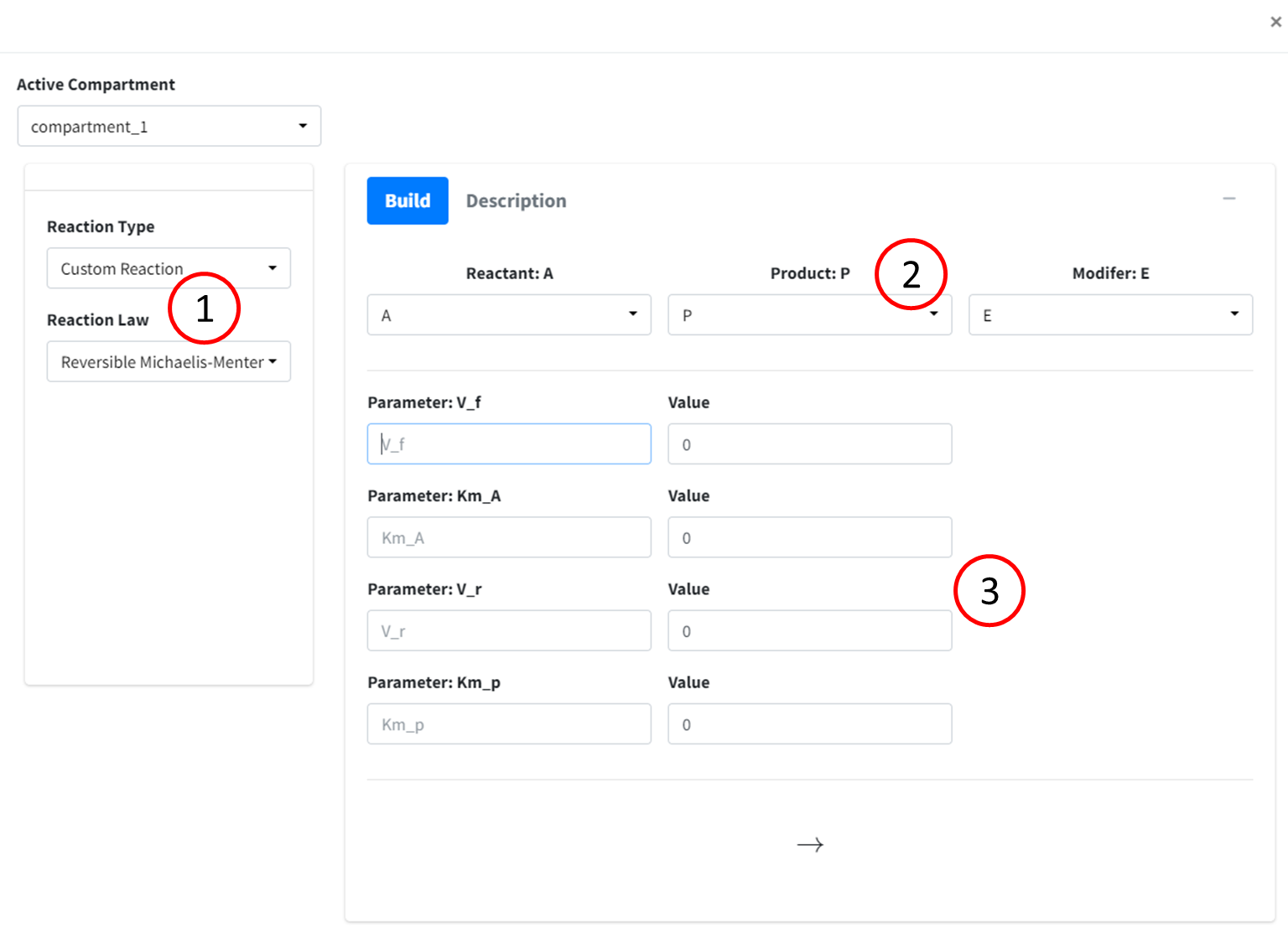


In the above:

1. Area to select custom reaction.
2. Options to add reactants, products, and modifiers based on what was entered in the law build.
3. The parameters in the equation law to be added.

Simply fill out the form as you would like any reaction, press the **Add** button and the reaction will be saved using your new law.

# Creating a Custom Equation

This option is for cases when a parameter is not a constant value. Often, parameters can be dependent on concentration levels, other parameters, or time. For these, a custom equation must be built for the interaction. This section will go over how to add these equations and how to navigate through the UI for this section of the application. To begin, navigate to “Modeler’s Toolbox -> Custom Equations”. The following box should be displayed:


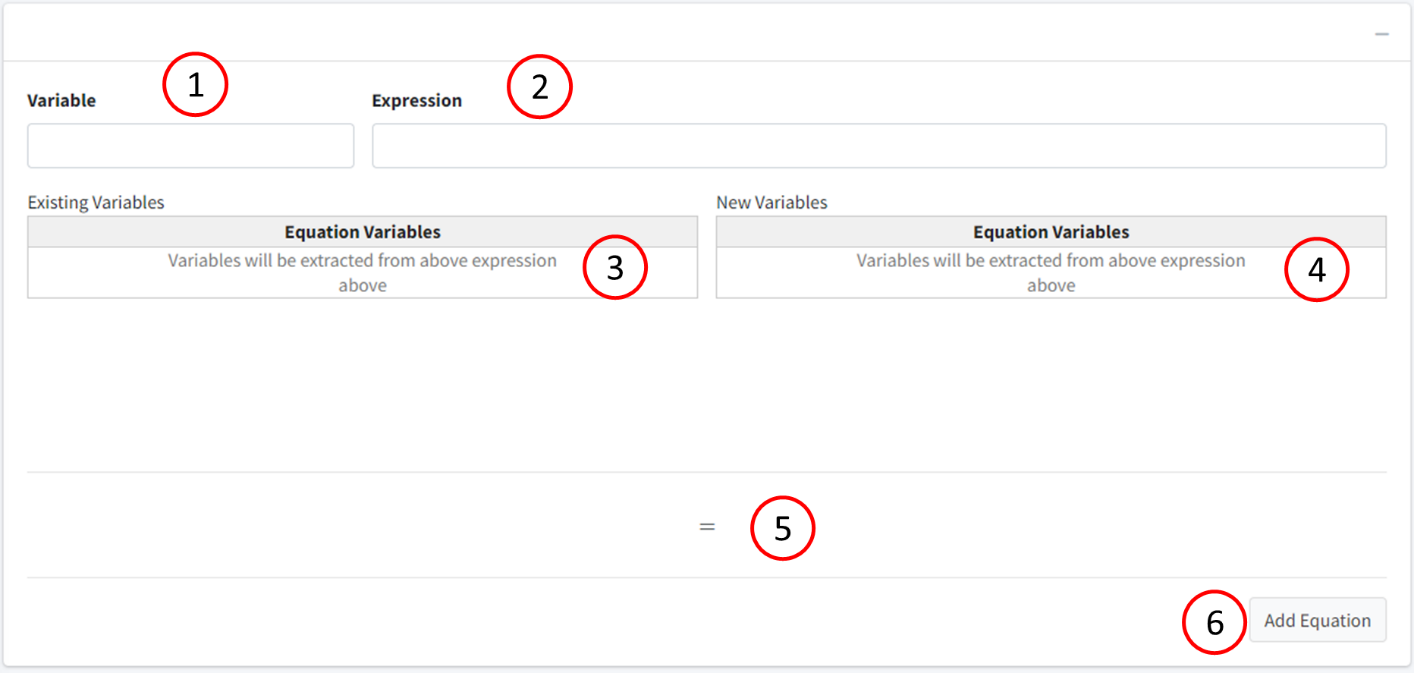


1. Box to enter variable that is dependent on the expression.
2. Mathematical expression for variable
3. Table displaying variables that are already in model.
4. Table displaying variables that are not in model.
5. Reaction display of custom equation.
6. Button to store custom reaction.

# SBML Import

Our application can import SBML files of level 2 and lower. The importer is written to look for certain tags and pull those, and not to particularly read on a level-by-level basis. For this reason, it does have the ability to read level 3 files, but some information could be lost in the process. Our import function was written completely from scratch including code to parse the xml document, extract, and process relevant features, and properly parse MathML equations. As such it is still an ongoing project as we test more and more SBML files. We encourage users to submit SBML files that present issues for them to either our GitHub issues section: <https://github.com/MCWComputationalBiologyLab/BioModME/issues> or to author [rdash@mcw.edu](mailto:rdash@mcw.edu).

Current Known Limitations:

- MathML parser cannot read expressions with root and degree (other keywords may be an issue)
- Models are rendered in as unitless. We do not currently parse UnitDefinitions in SBML markup.
- Other sections of SBML not currently parsed include “listOfInitialAssignments” and “listofEvents”.
- While we can read function definitions and use those to build equations, we have not currently incorporated a converter to add the functions to the UI. As such, all functions will load as type “CUSTOM”.

# Export Model

The model created in the application can be exported in a variety of ways including to different coding languages (R, MATLAB) and storage file types (rds, SBML). Model information can be exported as txt, csv, pdf, or LateX in a variety of tables. Lastly, the differential equations can be exported in a variety of formats including text, LateX, MathJax, and MathML.

## Coding Languages

Once the model is created, it can be exported to different coding languages for the users’ different purposes. This export converts the code into a full script that is ready to be run right off download. However, due to the complexities and syntaxes of different languages, the files might need some editing from the user (ex. User may need to use a different ODE solver or change the variable syntax of specific species).

### MATLAB

To export to MATLAB, navigate to the “Export” tab and move to the “code” box. Name the file and press “MatLab Code”.

The code will be generated in the following code blocks:

**Driver Block:**

This block will define the time, load initial conditions and parameters, run the model, and plot all species. It is set to run ode15s as the ODE solver (equivalent of lsoda).

**Model Function:**

This is a function that contains all model information. It begins by assigning names to the species and parameters in the model, then defines the differential equations used in the model. Any custom equation will be added and shown before the differential equations of the model. Lastly, it packages the results to a vector to return to the solver.

**Parameter Function:**

The function “function p = pars()” will assign parameter values to the model. Each line is commented with the parameter name. The parameter values are packaged to a vector and returned.

**Initial Conditions Function:**

The function “function y0 = Initial_Conditions()” will assign initial conditions to the model species. Species names are commented at the end of each line. Returned as vector.

**Notes:**

Variable names in BioModME can have “.” separating terms in a name. This is not a valid MATLAB syntax. By default, the export will convert all “.” to “_”. For example, “my.new.variable” will be converted to “my_new_variable”.

On export, variable numerical values will default to using “base units”. These are the standard units that BioModME uses in the backend to process the model. (mol, L, min). This can cause some values to differ from those displayed in the program if different units have been used.

### R

To export to R, navigate to the “Export” tab and move to the “code” box. Name the file and press “R Code”.

The code will be generated in the following code blocks:

**Model Function:**

This is a function that contains all model information. It takes in named vectors of initial conditions (state) and parameters (parameters) and passed them onto the functions within. The body of this function contains the differential equations of the model and returns a list of values for each species in the model.

**Driver Block:**

Below the model function, the driver of the code takes place. It begins by creating named vectors for parameters and initial conditions, generates a time vector, then runs a “lsoda” solver for the odes.

**Notes**

On export, variable numerical values will default to using “base units”. These are the standard units that BioModME uses in the backend to process the model. (mol, L, min). This can cause some values to differ from those displayed in the program if different units have been used.

## Storage Languages

BioModME can export the model information in a few different storage forms. These are not scriptable text but rather containers that store the model information to be used again by this application or others.

### rds

rds is an r-data structure that can be used to store data in R. This creates a list type structure user R notation. This is the main data storage type to use with this application as it will store all model information and relevant model features that are used in the application. This data type can also be read directly into R but, currently, R does not have a method of processing the structure outside of using BioModME.

### SBML

SBML (systems biology markup language) is a common data structure used in the modeling community that uses an xml style markup. Our application generates level 2 SBML type files that contain the following tags:

1. <listOfCompartments>
2. <listOfFunctionDefinitions>
3. <listOfSpecies>
4. <listOfParameters>
5. <listOfReactions>
   1. <listOfReactants>
   2. <listOfProducts>
   3. <listOfModifiers>
   4. <kineticLaw>
      1. <math>
      2. <listOfParameters>
6. <listOfRules>

Notes:

Reactions from the Import/Output sections of BioModME will be exported as Reactions where flows in will be considered products and flows out will be considered reactants.

As of v1.0 (11/14/23) our SBML export does not include unit definitions. This will be added in our future update.

## Model Information Tables

Navigate to the “Export” tab. In the bottom box, there are tabs corresponding to tables for species, compartments, parameters, and equations in the model. Clicking each tab of the tab box will change the table. Above each table are buttons for Copy, CSV, Excel, PDF, and Print that will export the respective table in that method.

- Copy – Copies the table to a clipboard to copy and paste.
- CSV – Downloads a csv file of the table.
- Excel – Downloads an xlsx file of the table.
- PDF – Downloads a pdf file of the table.
- Print – Sends the table to print.

We have created a new system that we are adding to our tables in the application. The following buttons have been added above tables:


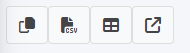


From left to right:

1. Copy – copies the entire table to computer clipboard (can be pasted to program of your choice)
2. Download csv – downloads table to comma separated value file.
3. Download xlsx – downloads table to excel file.
4. Open in New Window – Opens table to new window, separated from main app.

## LateX Document

The ability to download the model information in LaTeX format is a part of this application. This option will generate all the TEX needed to display the model in a formatted way. This option will also subset all variable names (i.e k_f1 -> k_f1_). To use this option, navigate to the “Export” Tab and select “LaTeX Download” button after providing a name. A popup will appear asking the user to select what pages they want displayed in the document. After selecting, simply click “Get Document” and the LaTeX document will download and can be opened in your preferred TEX editor.


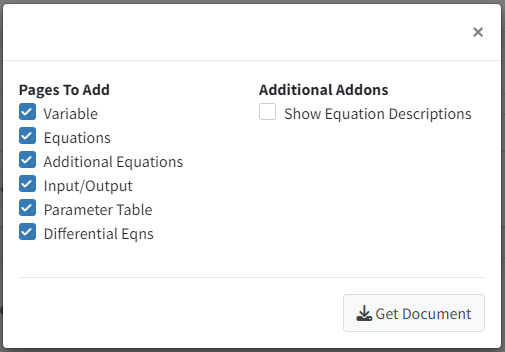


## Differential Equations

The differential equations in the model can be downloaded in a few ways. One option is to download them in the above LaTeX document. As current, long differential equations will be run off the LaTeX page though, so the user will need to add the line breaks themselves if they wish to see them in that format.

The different equations can be downloaded in their LaTeX form and MathML form by navigating to the “Create Model” tab and the scrolling down to the “Differential Equations” box. Here your different equations are displayed one-by-one. Simply right-click on the equation you wish to get the underlying code for and navigate to “Show Math As -> MathML Code” to download the MathML version or “Show Math As -> TeX Commands” to get the TeX version of the equation.


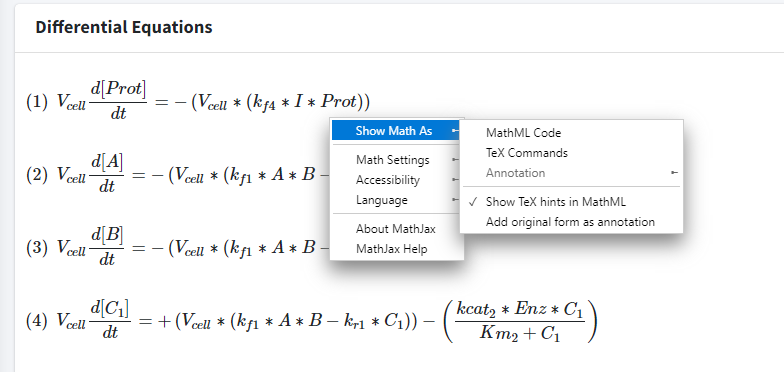


Much the same, you can display all the equations in their native plain TeX commands by right-clicking on the equation and going to “Math Settings -> Math Renderer -> Plain Source”.


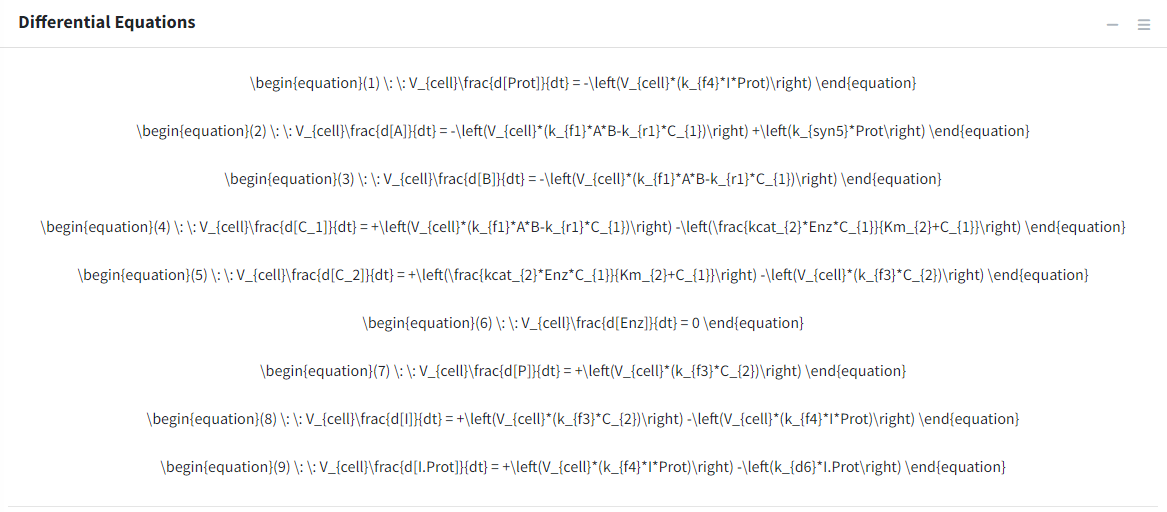


### Content and Presentation MathML

MathML comes in two forms: content and presentation. Above we present one option to generate presentation MathML, albeit it can be a bit slow if you want all our equations. As such, we have provided the functionality to quickly download multiple equations at once in a simple fashion. The figure below shows the “Differential Equations” box on the main page. At the bottom we have added a “Download Button”.


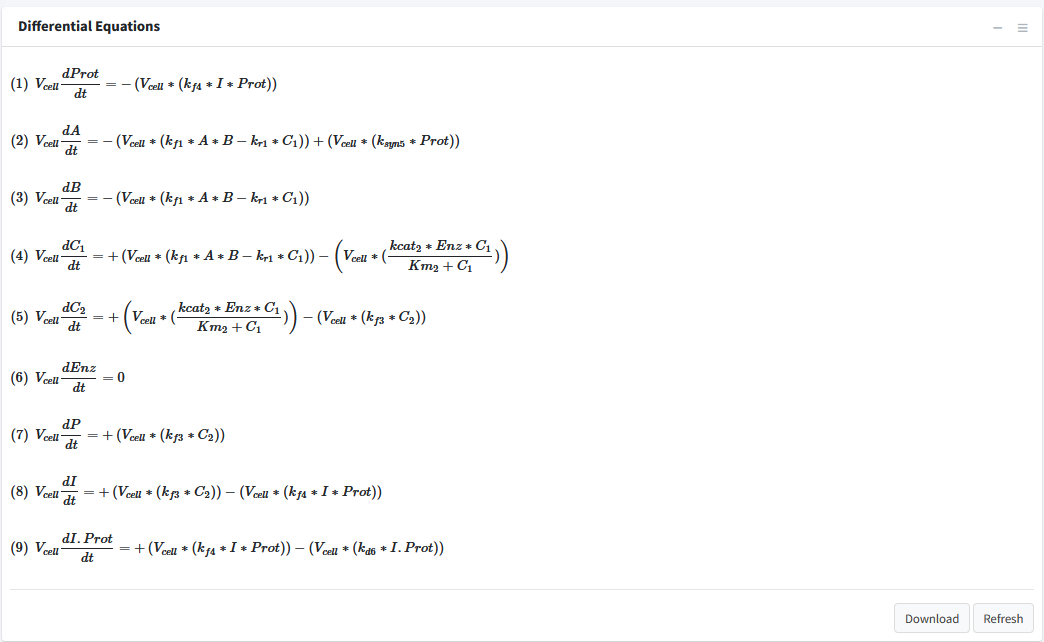


Pressing the “Download” button will create a popup for the user to download the equations in a variety of formations including both version of MathML. The popup:


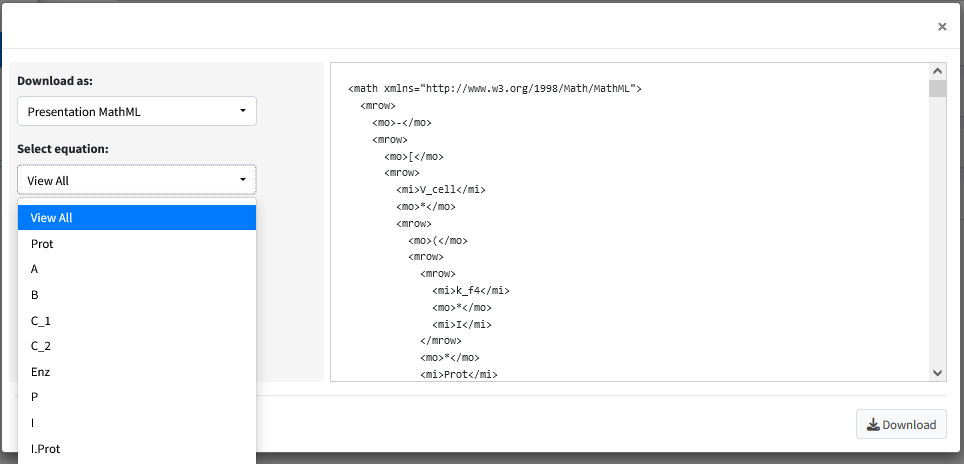


The label **Download as:** contains the following download options: Plaintext, Content MathML, Presentation MathML, and Latex. The **Select equation:**  dropdown allows the user to view all the differential equation or separate them by species. From there the MathML equation can be Copy & Pasted from the textbox to the right or downloaded to a text file using the “Download” button.

# Tutorials

In the following sections, we will go through three types of tutorials. The first is a basic tutorial that will show the user how to create a basic model in a single compartment. The second is a more complicated model focusing on the input/output between compartments, detailing how to set up those flows. The final is a basic model to perform parameter estimation. We recommend starting at the beginning tutorial if new to the application.

Notes: We have changed the UI of the addition buttons recently upon suggestions from our reviewers. There will be the following difference in button placement in the tutorials:


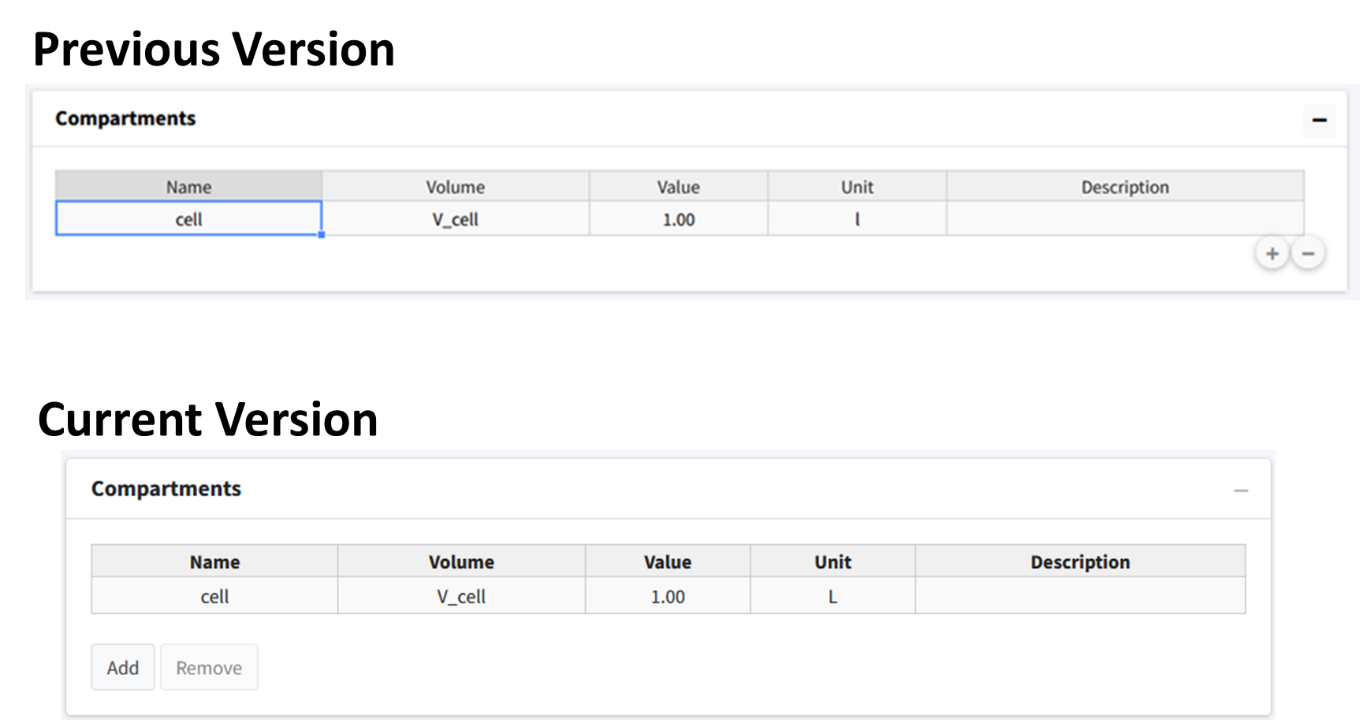


Where the “+” symbol is now “Add” and the “-“ symbol is now “Remove”.

# Basic Tutorial - Introduction

This tutorial covers basics features of BioModME by building a simplified, single region model. In this example, we focus on a set of reactions occurring in a cell. Compound “A” is synthesized by “Prot” at a constant rate and reacts with a limited supply of compound “B” found in the cell to make an intermediate “C1”. C1 undergoes an enzymatic conversion to “C2” and this new intermediate undergoes self-cleavage to the desired end product, “P”, and an inhibitor, “I”, of Prot. This inhibitor feedback binding to Prot stopping the synthesis of A, and thereby the entire reaction scheme.

In the following sections of this tutorial, we will design the above system and look at how this program can be used to visualize and modify the data. The contents include the following:

1. Creating model variables
2. Building a system of equations
3. Entering the inputs and outputs of the system
4. Inputting numerical parameter values
5. Solving and plotting the differential model
6. Exploring the different types of plotting features
7. Exporting the model and its features in meaningful ways

To use the online version of this application visit <https://biomodme.ctsi.mcw.edu/>. Your current model can be downloaded locally using the “Export” tab. The file format is an .RDS file. This file can be loaded in by opening the right sidebar of the application. A local version of the application can be directly downloaded at <https://github.com/MCWComputationalBiologyLab/BioModME> and opened using the R programming language using stand Shiny app protocol. Refer to documentation for pulling a docker version of the application.

## Constructing Model

This section will cover how to build and construct a model.

## Define Variables

The application loads on the “Create Model” tab. Here, we will build our model, setting up variables, equations, and parameters. Each of these components are their own box on the page. On load, the application will create a starting compartment named “comp_1”, which can be seen in the “Compartments” box. Click on the “Name” cell of the table to change the compartments name (cell). We will also change the volume name here (V_cell). In a single region model, the physical volume is not of much importance so we will leave that as a value of 1 to avoid any model scaling effects.


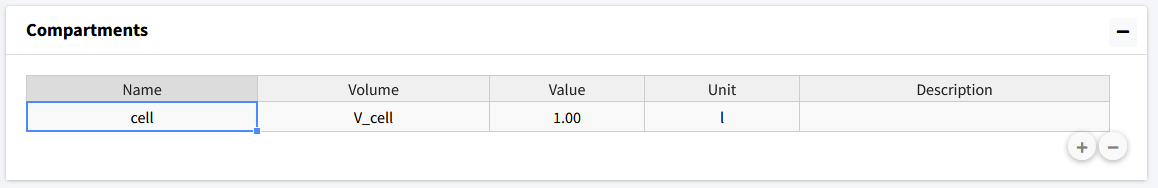


Next, the model species will be added. To add a species, move to the next box (“Species”). Here we press the button with a plus icon to add species whose information can be edited in the table.


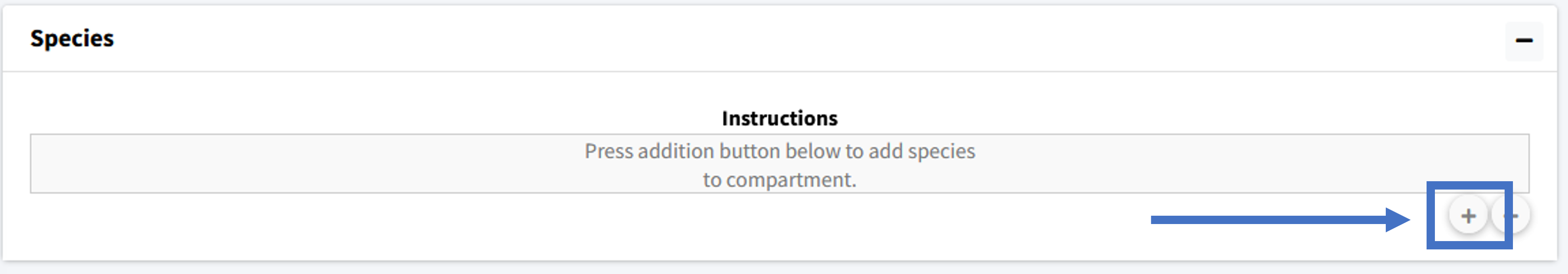


We will add nine species to the model and then rename them appropriately: Prot, A, B, C_1, C_2, Enz, P, I, I.Prot.


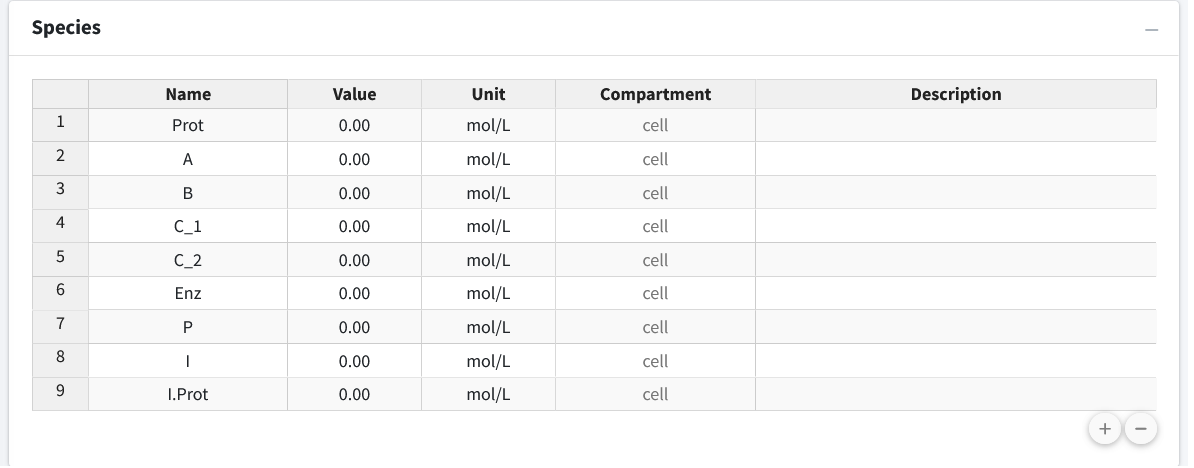


The second column of the species is “Value”, which is the initial amount of that species in the model. For this model we have the following initial concentrations:

- Prot = 3
- A = 0
- B = 5
- C_1 = 0
- C_2 = 0
- Enz = 0.5
- P = 0
- I = 0
- I.Prot = 0


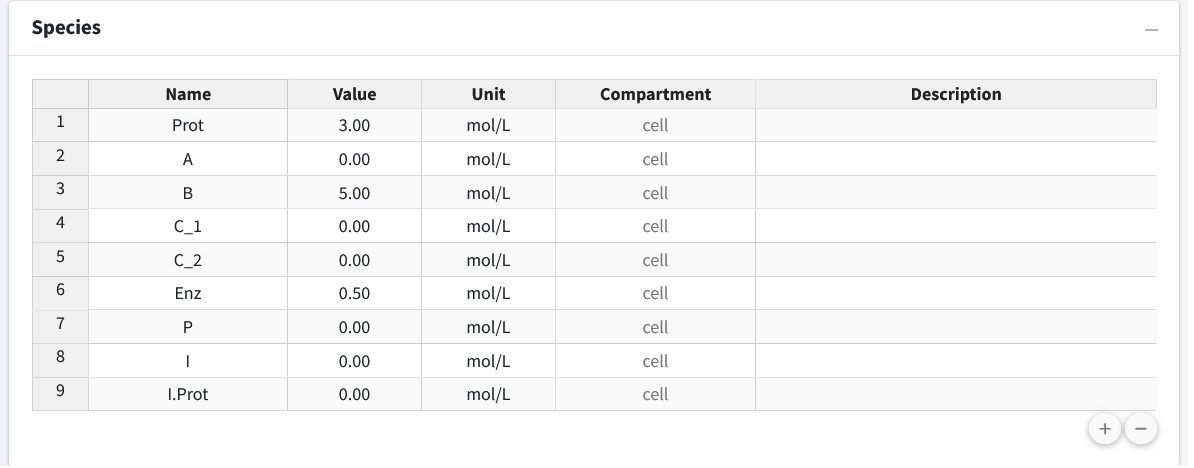


Lastly, if desired, we can add descriptions to our species to remind ourselves (or let other users) know what the species in the model are. Below, we have entered brief descriptions of the beginning species. Editing can be done by clicking the appropriate cell in the species table.


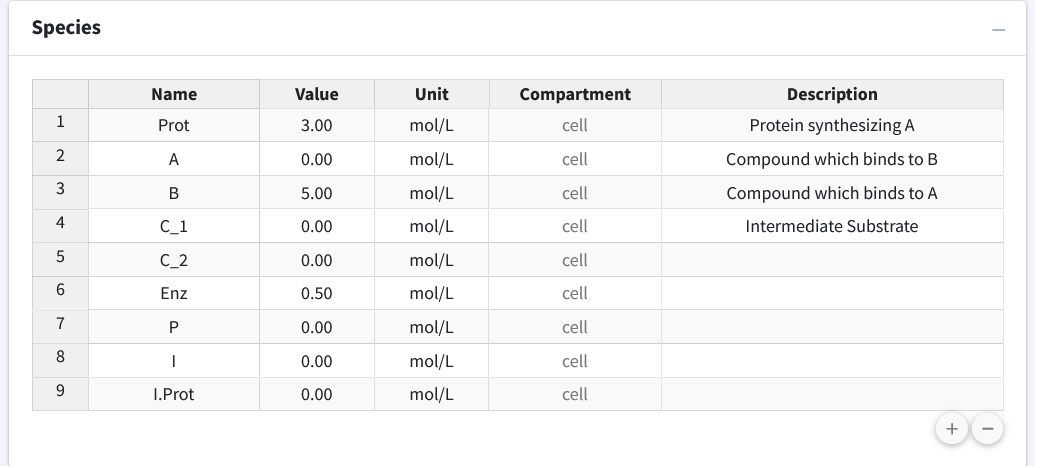


## Create Reactions

Scroll down to the “Reactions” box in the “Create Model” tab. In this section we will build up the sets of reactions that make up our reaction network. Press the “+” button to begin adding equations.

### Equation 1 - Law of Mass Action, Reversible

[
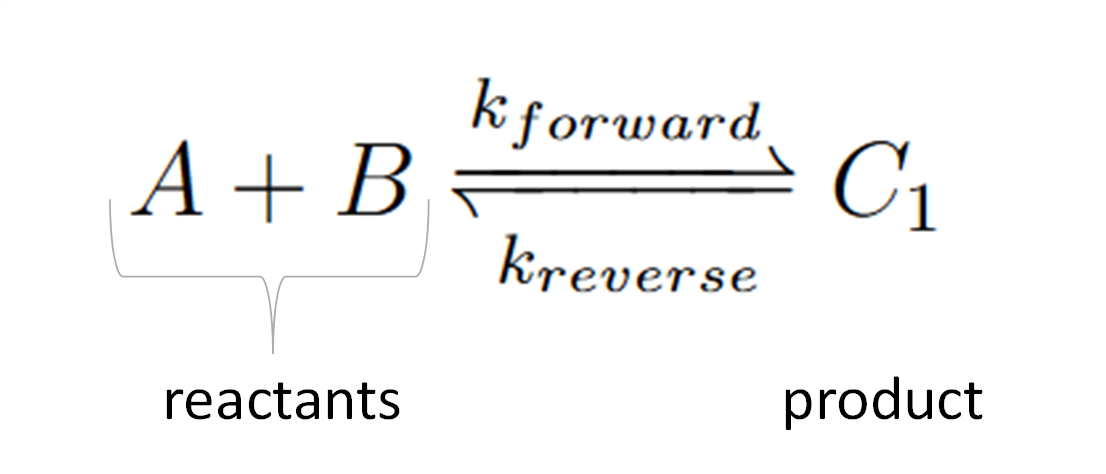
](file:///C:\Users\ju61191\AppData\Local\Microsoft\Windows\INetCache\Content.Outlook\7ZRPO71E\_images\eqn1.png)

Chemical reactions like this follow the **law of mass action**. This first equation is a reversible reaction. The equation consists of two reactants, one product, and moves in both directions where are forward rate constant is “k_f1” and our reverse is “k_r1”.

This reaction input can be seen in the below figure. The steps for the following equation are:

1. Select “Chemical Reaction” for equation type.
2. Select “Mass Action” as the law.
3. Select “Reversible” as the reaction direction.
4. Set the equation to have two reactants and one product.
5. Fill out equation information. Rate constant names are autogenerated for all equation types using the number of the current equation as their number at the end.
6. This is the equation builder that shows what your built equation looks like.
7. Press the “Add Equation” button.


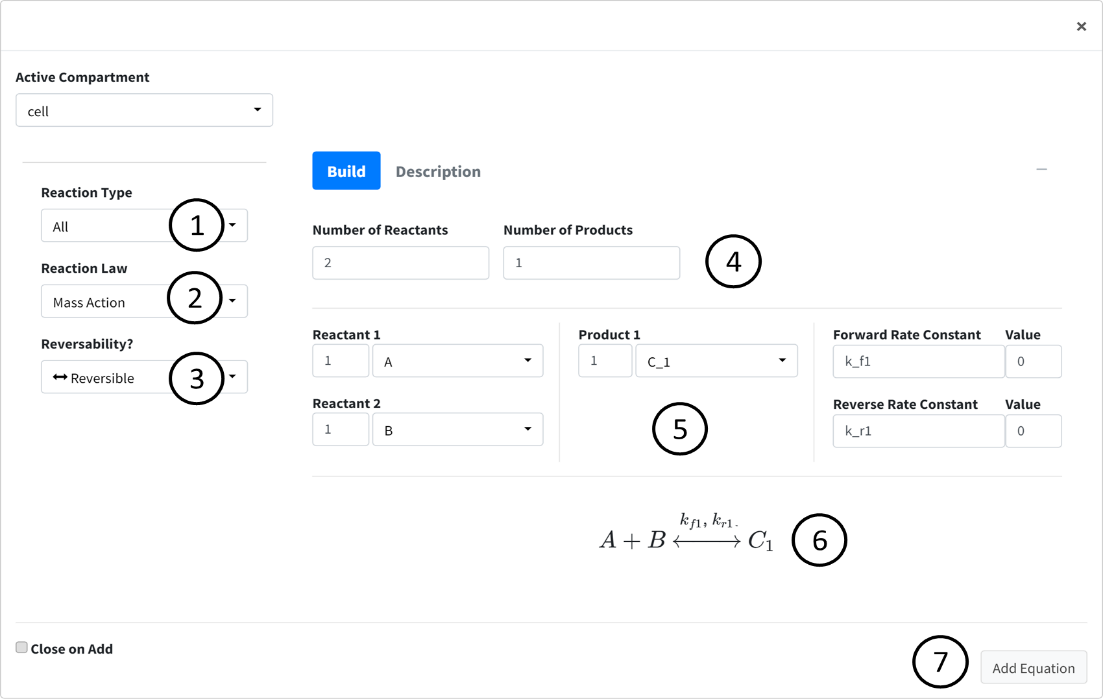


### Equation 2 - Michaelis-Menten Kinetics


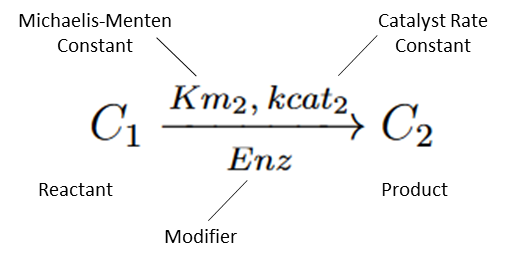


Next, we enter the second equation, conversion of C1 to C2 by the enzyme “Enz”. These reactions follow Michaelis-Menten kinetics. Figure 7 shows how this equation looks entered the program.

The steps are as follows:

1. Change the Reaction Type dropdown to “Enzyme Based Reaction”.
2. Select “Michaelis Menten” as the Law.
3. Enter the equation information into the builder.
4. Press the “Add Equation” Button.


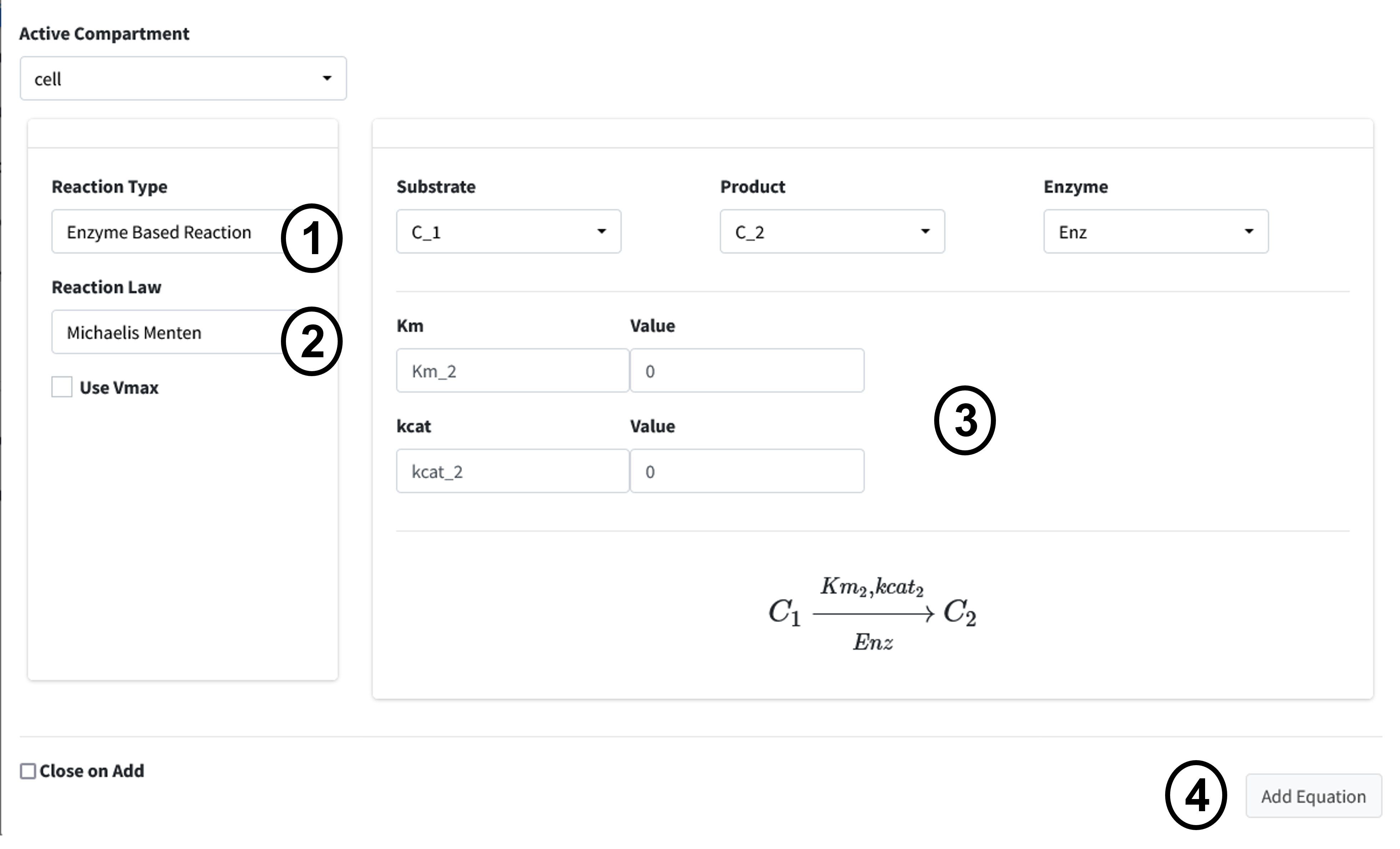


### Equation 3 - Law of Mass Action, Unidirectional

[
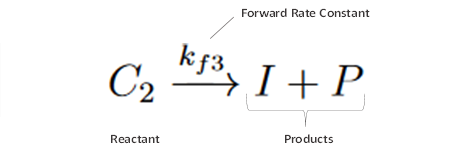
](file:///C:\Users\ju61191\AppData\Local\Microsoft\Windows\INetCache\Content.Outlook\7ZRPO71E\_images\eqn3.png)

The next reaction is a conversion of C2 to product P and inhibitor I. This is a one directional reaction with one reactant and two products following the law of mass action.

1. Change the Equation Type dropdown to “Chemical Reaction”.
2. Select “Mass Action” as the Law.
3. Change the reversibility option to “Irreversible”.
4. Set the Number of Reactants to “1” and the Number of Products to “2”.
5. Enter the equation information into the builder.
6. Press “Add Equation” button.


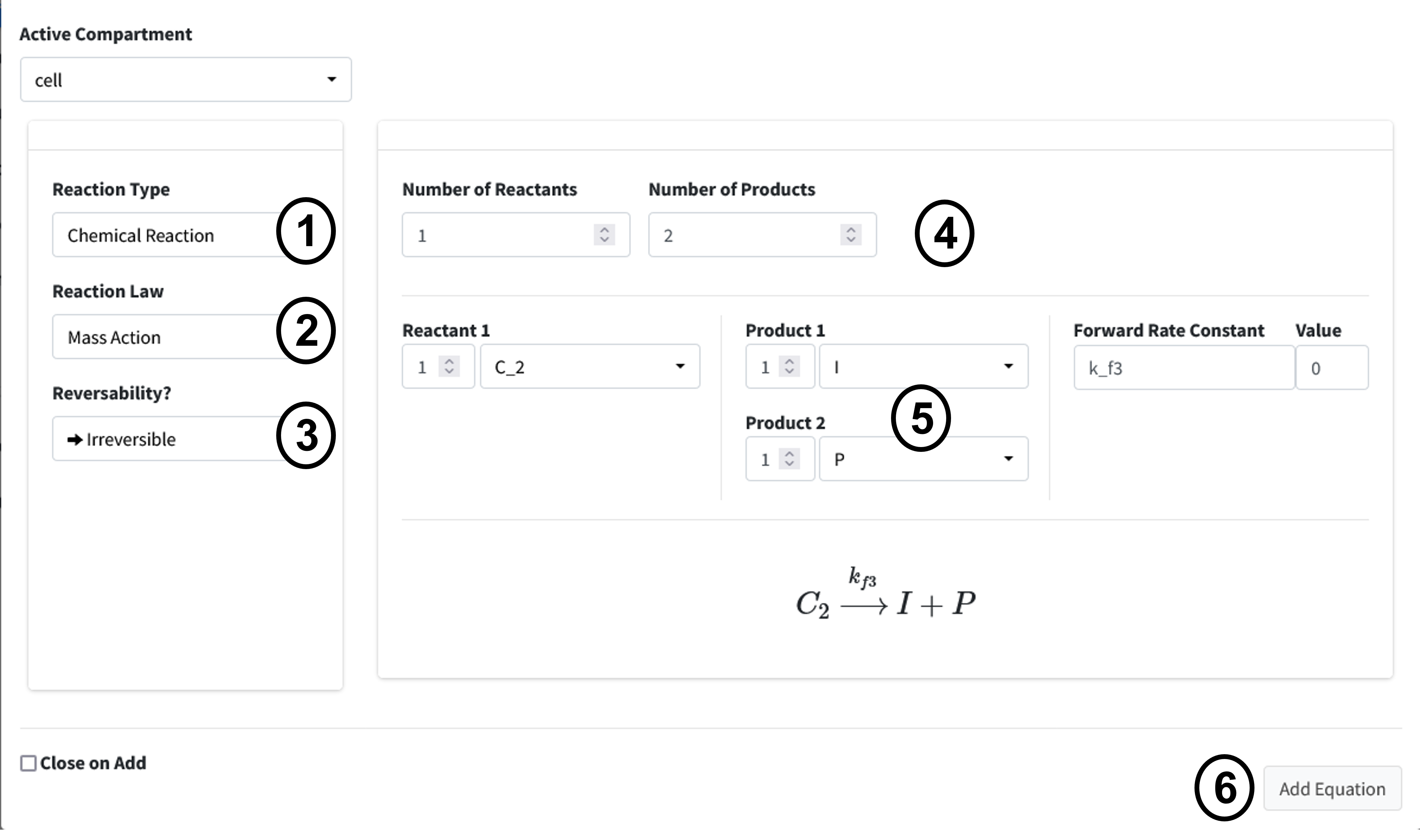


### Equation 4 - Law of Mass Action, Unidirectional

[
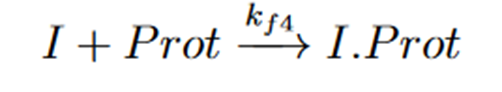
](file:///C:\Users\ju61191\AppData\Local\Microsoft\Windows\INetCache\Content.Outlook\7ZRPO71E\_images\eqn4.png)

Our next reaction is the inhibitor of Prot. This is another chemical reaction following the law of mass as in Equations 1 and 3 with two Reactants I and Prot converted to I.Prot. The user interface input is below in Figure 9. The steps should be familiar to your by now, but they are as follows:

1. Change the Reaction Type dropdown to “Chemical Reaction”.
2. Select “Mass Action” as the Law.
3. Change the reversibility option to “Irreversible”.
4. Set the Number of Reactants to “2” and the Number of Products to “1”.
5. Enter the equation information into the builder.
6. Press “Add Equation” button.


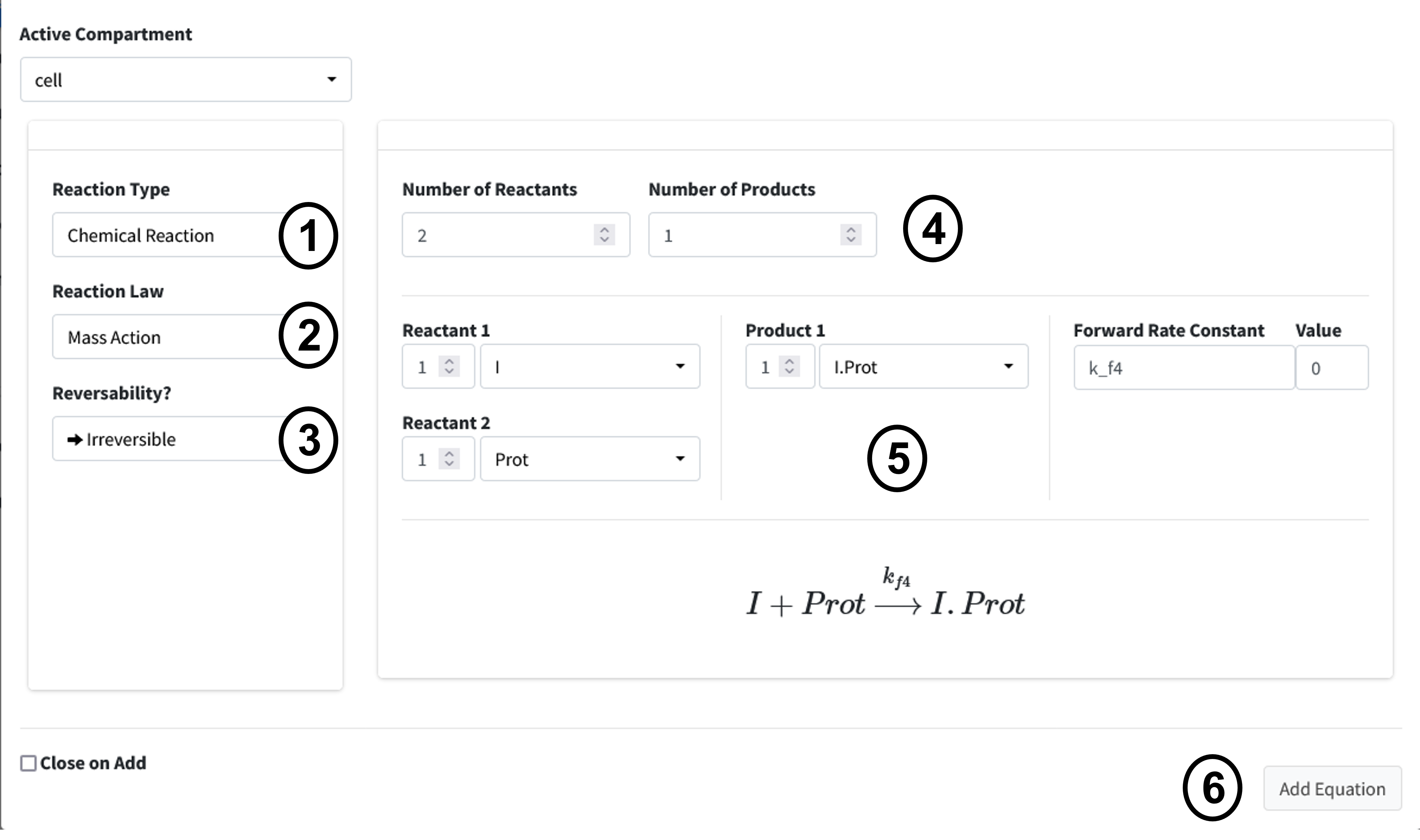


### Equation 5 - Synthesis by Factor


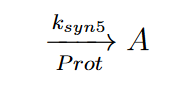


Synthesis Rate Constant

Factor Promoting Synthesis

Synthesized Product

Our next reaction is the synthesis of A by Prot, where Prot is a factor promoting synthesis (as opposed to being directly converted).

1. Set the Reaction Type to “Chemical Reaction”.
2. Change the Reaction Law dropdown to “Synthesis”.
3. Click the “Factor Driving Synthesis?” checkbox.
4. Fill out equation builder with the species to synthesize and its corresponding factor.
5.
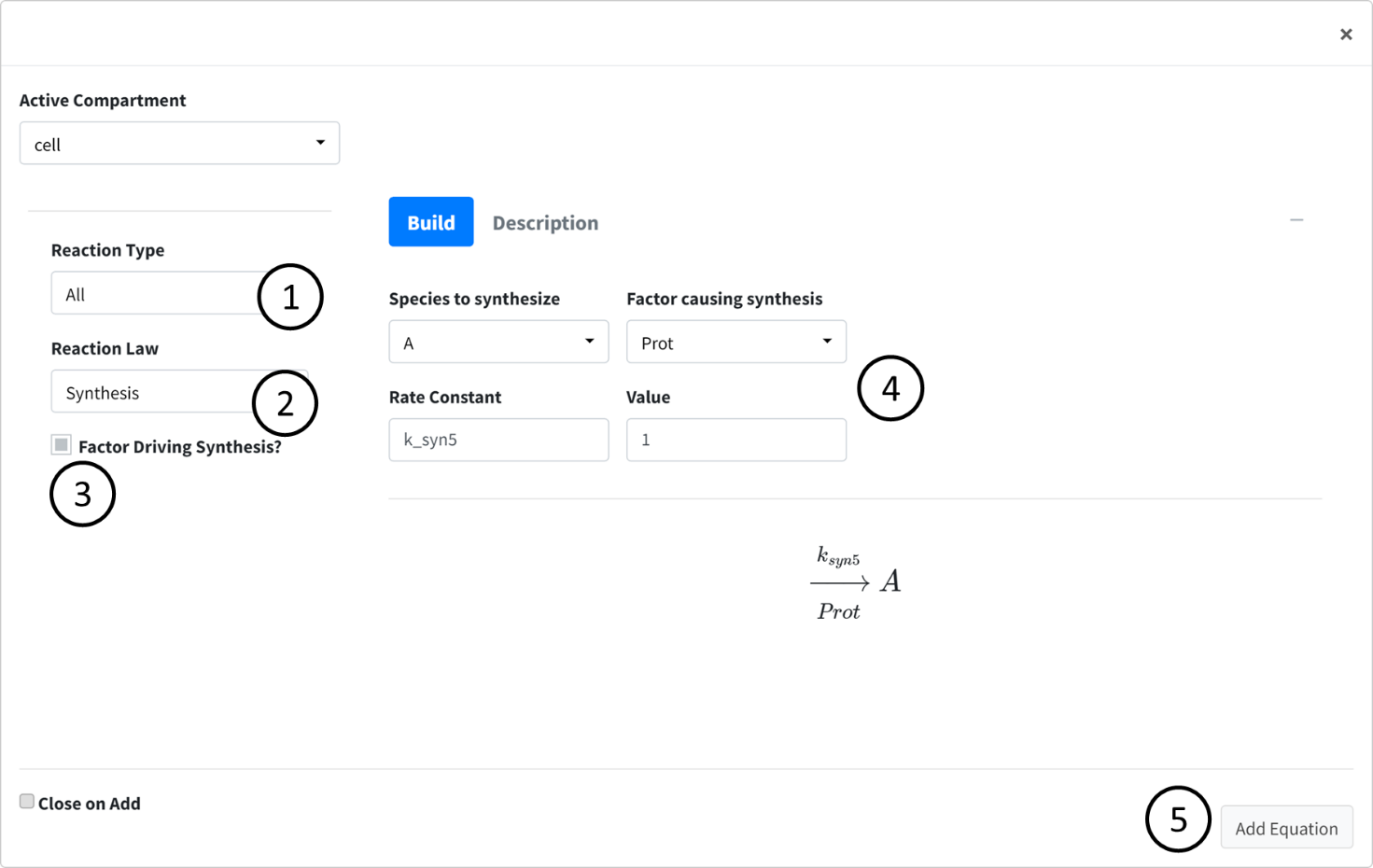
Press the “Add Equation” button.

### Equation 6 - Degradation by Rate

[
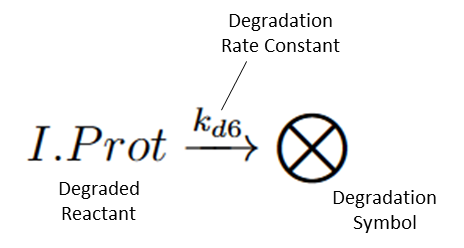
](file:///C:\Users\ju61191\AppData\Local\Microsoft\Windows\INetCache\Content.Outlook\7ZRPO71E\_images\eqn6.png)

Our final reaction is the degradation of I.Prot by a rate. This is useful for when we know the rate at which a protein is degraded in the cell but do not really know what is causing the degradation. These are often concentration dependent.

1. Set the Reaction Type to “Chemical Reaction”.
2. Change the Reaction Law dropdown to “Degradation (Rate)”.
3. Fill out equation builder with the species to degrade and its rate constant.
4. Make sure the “Concentration Dependent” box is checked.
5. Press the “Add Equation” button.


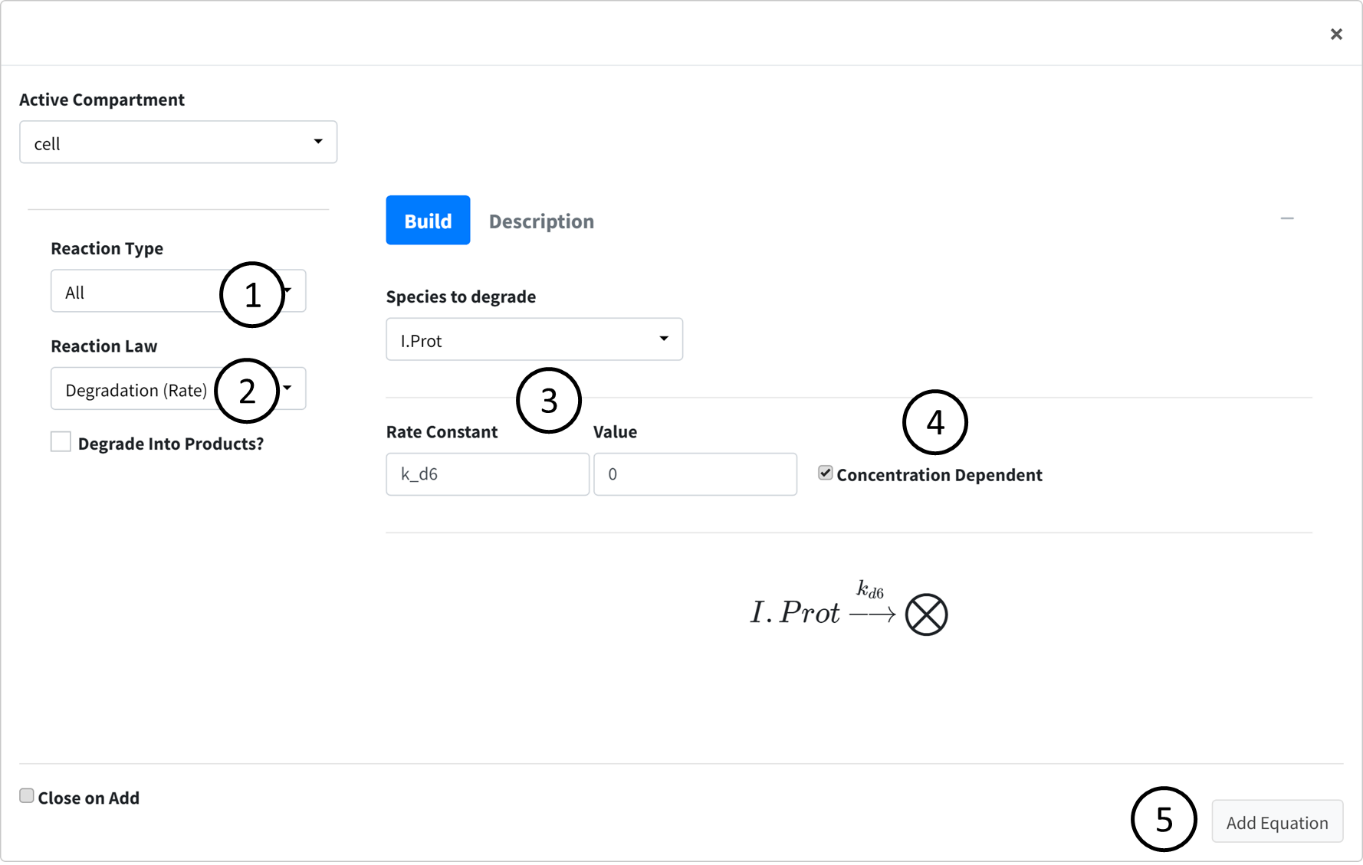


## Compartment Input/Output

The next box is the Input/Output box. This box is used to add flow/transfers between regions. Our model does not have any flows to account for so we will not use this box for anything.


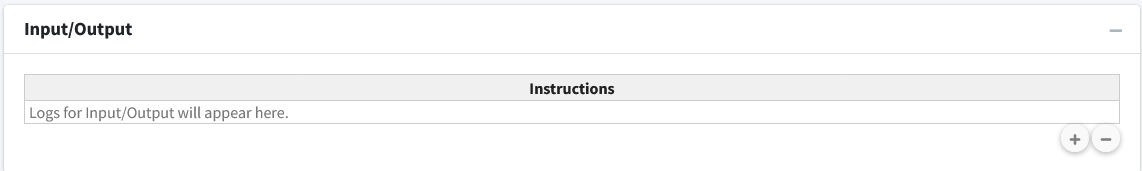


## Parameters

Navigate to the next box: **Parameters**.

Below is the generated parameter table that has been created so far by the above entered information. All descriptions are pre-generated based on where the parameter originated. Like other tables, all values are editable.


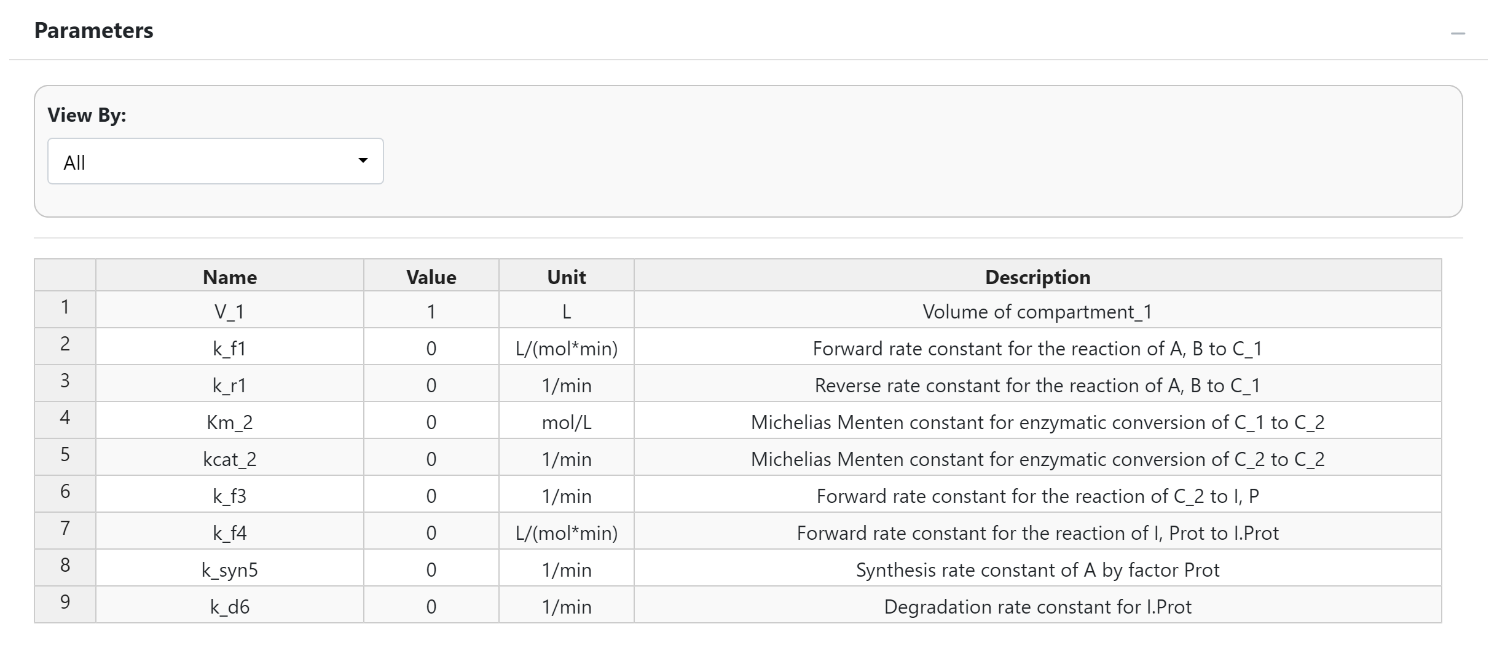


The parameter values for the model are as follows:

- k_f1 = 2
- k_r1 = 1
- Km_2 = 0.1
- kcat_2 = 3
- k_f3 = 2
- k_f4 = 2
- k_s5 = 0.5
- k_d6 = 0.1

The below figure shows the parameter table with the values entered.


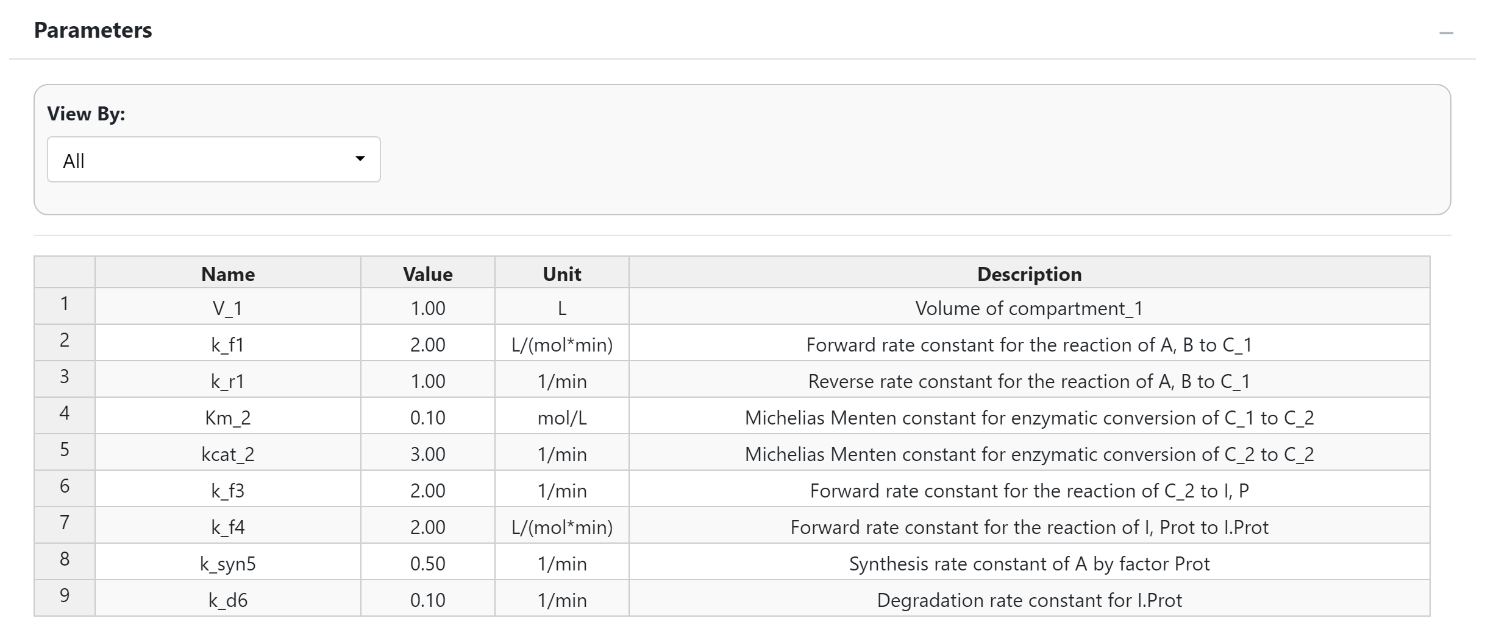


## Differential Equations

Navigate to the final box “Differential Equations”.

This tab is used to generate and see the mathematical equations that govern our model. This tab will begin with all species being equal to “NA” until the generate button is pressed or model is executed.

Differential equations are solved whenever an equation is added. The “Generate” button is used to refresh the viewing of the differential equations if necessary.


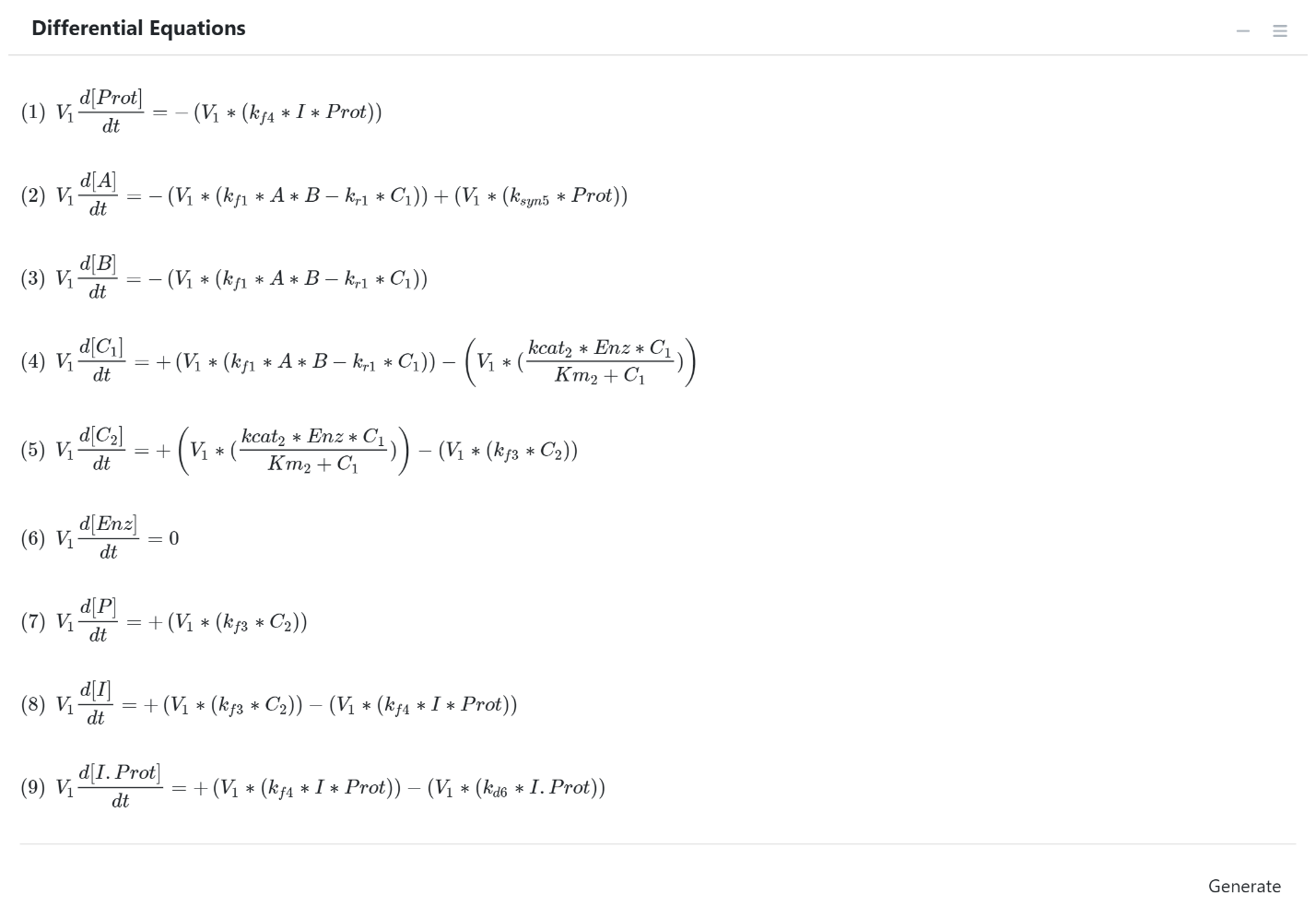


Note

The right sidebar of the “System of Differential Equations” box has an option for viewing the differential equations including turning off the math rendering and showing each derivation line as a newline for each differential equation.

## Solve Model

Navigate to the “Execute Model” tab on the lefthand tabbar. This tab solves our model and provides us with the options to customize the solver. For this model, we will just solve using the standard options:

1. Enter the following times: Starting = 0, End = 15, Step = 0.1, Unit = min. This generates the model to be solved and integrated at time points 0, 0.1, 0.2…..14.8, 14.9, 15.
2. Press the “Run Solver” button.
3. An output table should be generated with the concentration of each species along each time step of the solved model.

You can download this model using the buttons above the table. In order the buttons are: copy to clipboard, download to csv, download to xlsx, and open in new window.


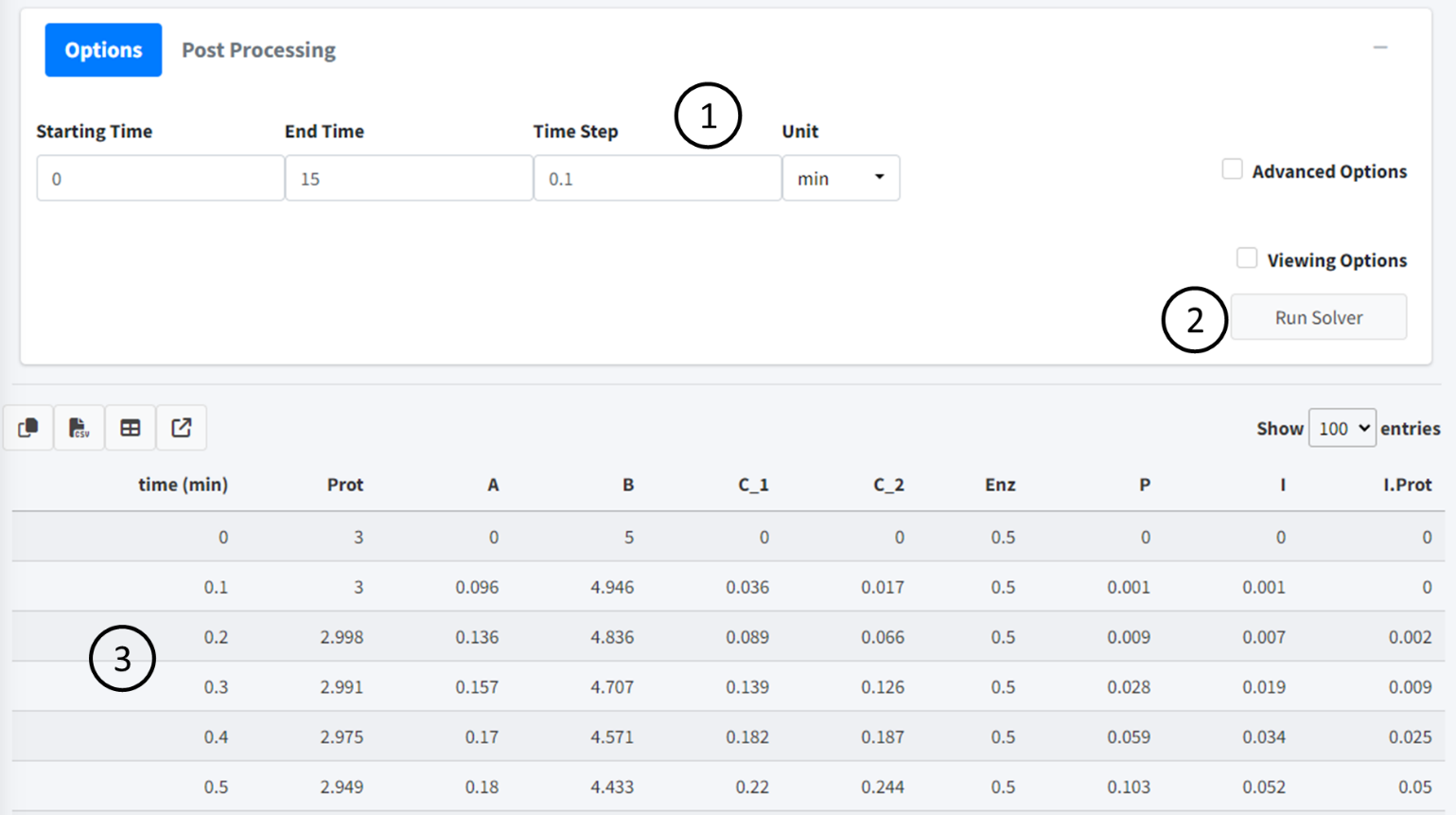


## Visualize Model

This section will cover how to visualize our created model.

Navigate to the “Visualization” tab on the lefthand tabbar.

The plotting tab has four modes, we will cover two of them. In this section we will cover “Normal Plot” which is the default when you enter the tab. The tab auto loads the plot when the model is solved, plotting all species of the model. These are the main features of the plot:

1. Variables - Change what species are plotting in the model. All species in the model are plotted by default.
2. Download - Dropdown containing options to download plot.
3. Options - Dropdown containing the following:
   - Plot mode - Changes the plotting types and features. Here we are plotting “Normal Plot”.
   - Plot Renderer – type of plot used. “plotly” is an interactive plot while “ggplot2” is the standard R plotting. The interactive is the default as it allows you to interact with the data using features such as zoom, pan, and hovering over data to view its value.
4. Plot - Plot area with adjustable sizing on bottom right corner.
5. Plot Options – Four dropdowns that allow the addition of labels, change of axis sizing, and control of line color and size.
6. Boxes containing menus to change model variables and import datasets to plot.


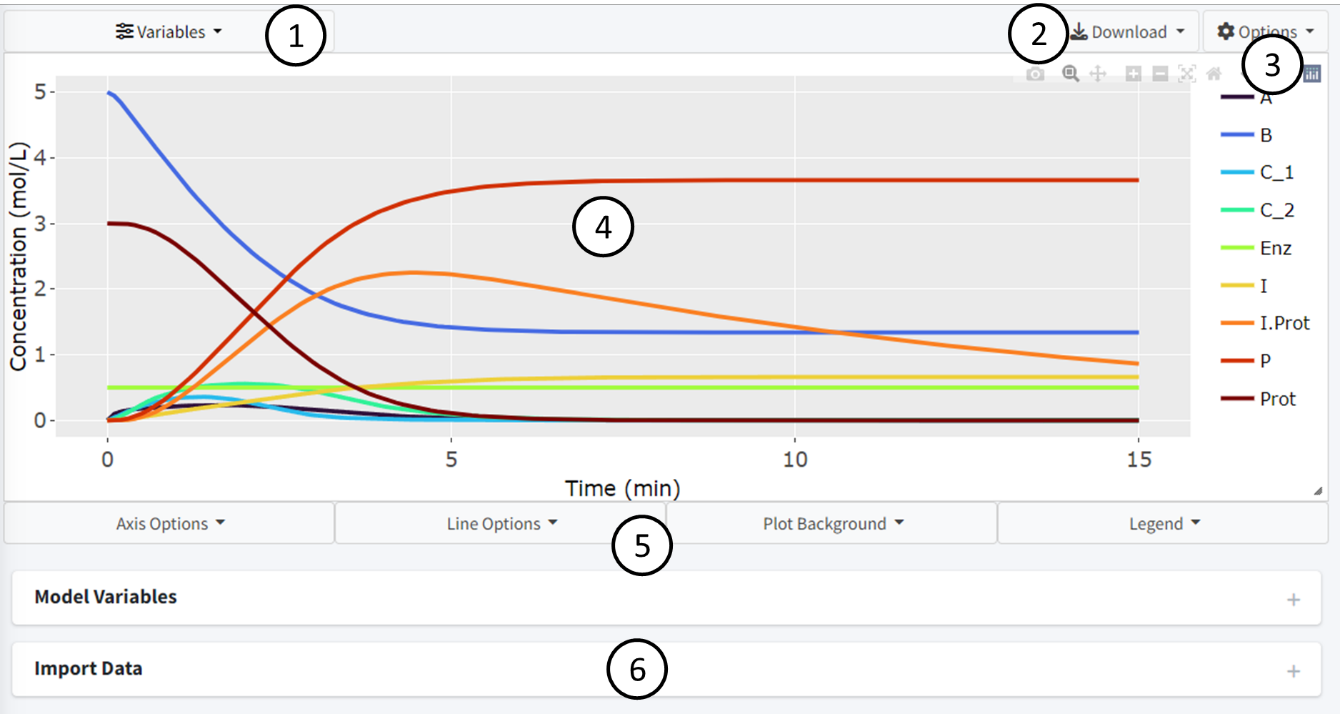


By default, the page should look like above when this tutorial model is solved. All variables are displayed using an evenly spaced rainbow pattern coloring.

Press the “Variables” dropdown to change the species plotted in the graph. Press the “x” on the species to remove them. The “Select All” button reselects all species while the “Reset” button removes all species.


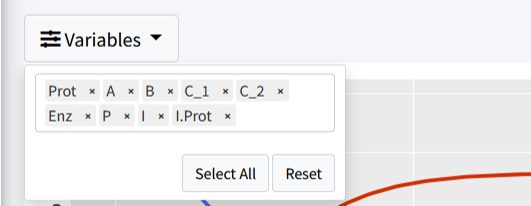


Let’s remove the following species to clean the plot up:

- C_1
- C_2
- Enz

We now have a less cluttered plot:


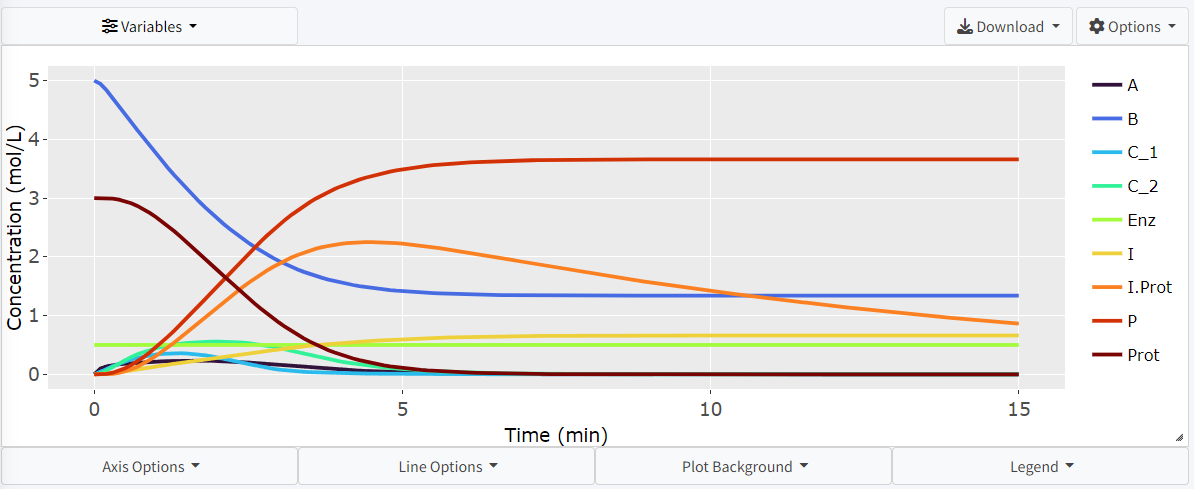


The interactive plot uses Plotly (<https://plotly.com/r/plotly-fundamentals/>) as a render and includes many of its base features including:

1. By clicking on a variable in the legend, they can be removed from the plot.
2. Double clicking a legend variable will remove all species but that one from the plot.
3. The top right bar includes options to pan, hover, and zoom the plot.
4. Press, hold, and drag the mouse on the plot to select specific features.
5. By hovering over lines on the plot, a popup will show with the values at that point.

There are many features included to let the user make the plot there own. These are all located in the dropdowns below the plot. Here, almost any feature of the plot is customizable. Feel free to play around and make the plot your own.

## Export

BioModME allows the built model to be exported into 4 main categories:

1. Prebuild, ready to run code, for either MATLAB or R.
2. An “.rds” file (type of R data file) that can be reloaded into the program. This is the way to save and load a model into the program currently.
3. A prebuild LaTeXdocument all main model information.
4. Downloading tables for parameters, equations, and initial conditions in csv, txt, or pdf form.


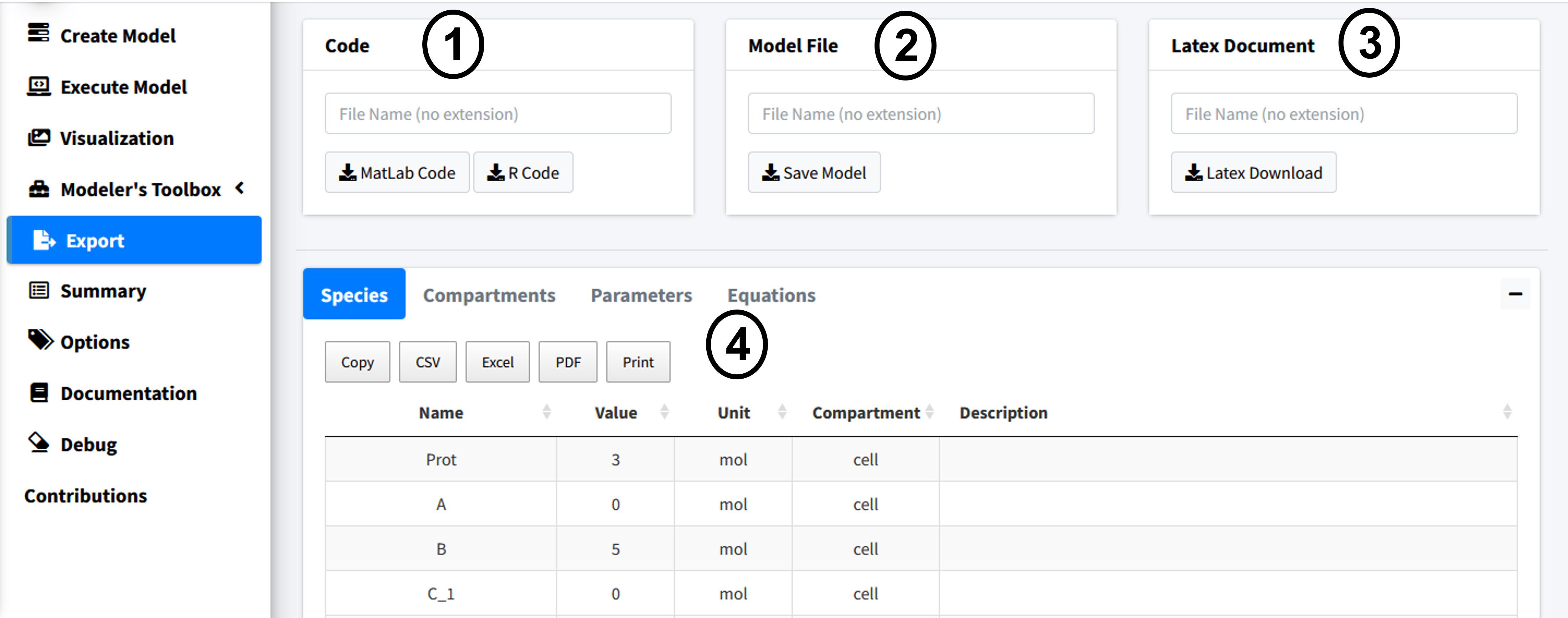


Feel free to try the code export if you have an interest in solving your model in those programming languages. Make sure to give any downloaded file a name.

LaTeXWhen downloading the LaTeXdocument a popup will appear, showing further customization options for that document.


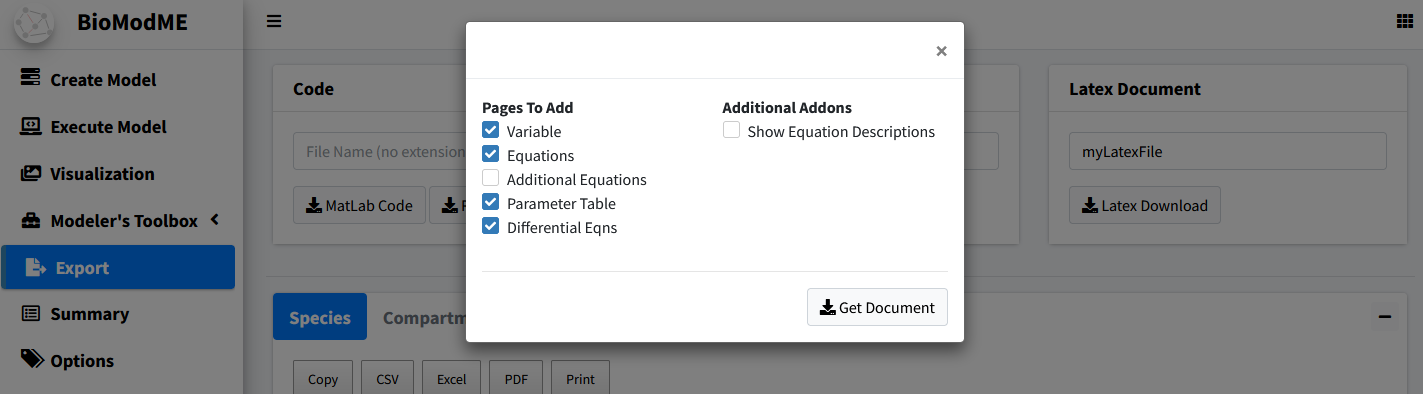


In this example, we will remove the “Additional equations” page as we did not create any non-standard equations. The generated document is created as a text file that can be copied to our LaTeXeditor of choice and run. I recommend using overleaf (www.overleaf.com) but any TeX editor will work. Below is a figure of the overleaf output:


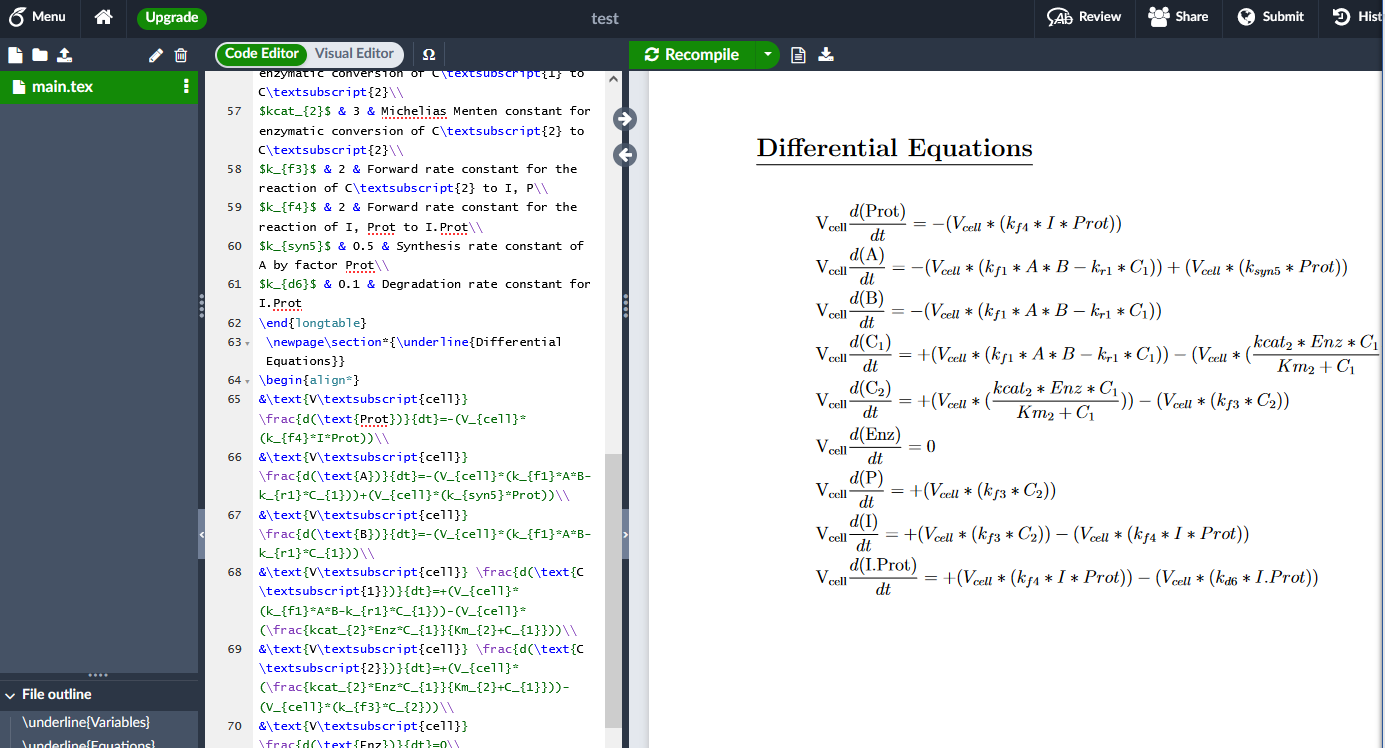


# Multi-Compartmental Model Tutorial

A bolus of a neutral drug “A” was administrated intravenously (i.v.) at time t = 0. Within the liver, “A” can diffuse (unidirectional) from liver blood region tissue liver tissue region via facilitated diffusion with Vmax (nmoles/min) and Km (Molar). Within the liver cells, “A” is converted to “B” via a chemical reaction that follows the law of mass action with a first order rate constant k1 (1/min). The product “B” can also diffuse (simple diffusion) between liver tissue region and blood region with a permeability-surface area product PS (ml/min). Within the kidneys, “B” is excreted from the blood region via a linear process with a rate constant ke (1/min). Here, the variable “V” is the compartmental volume of the respective compartment, numbered to differentiate between them. The variable, F, is our rate of flow leaving or entering a compartment.


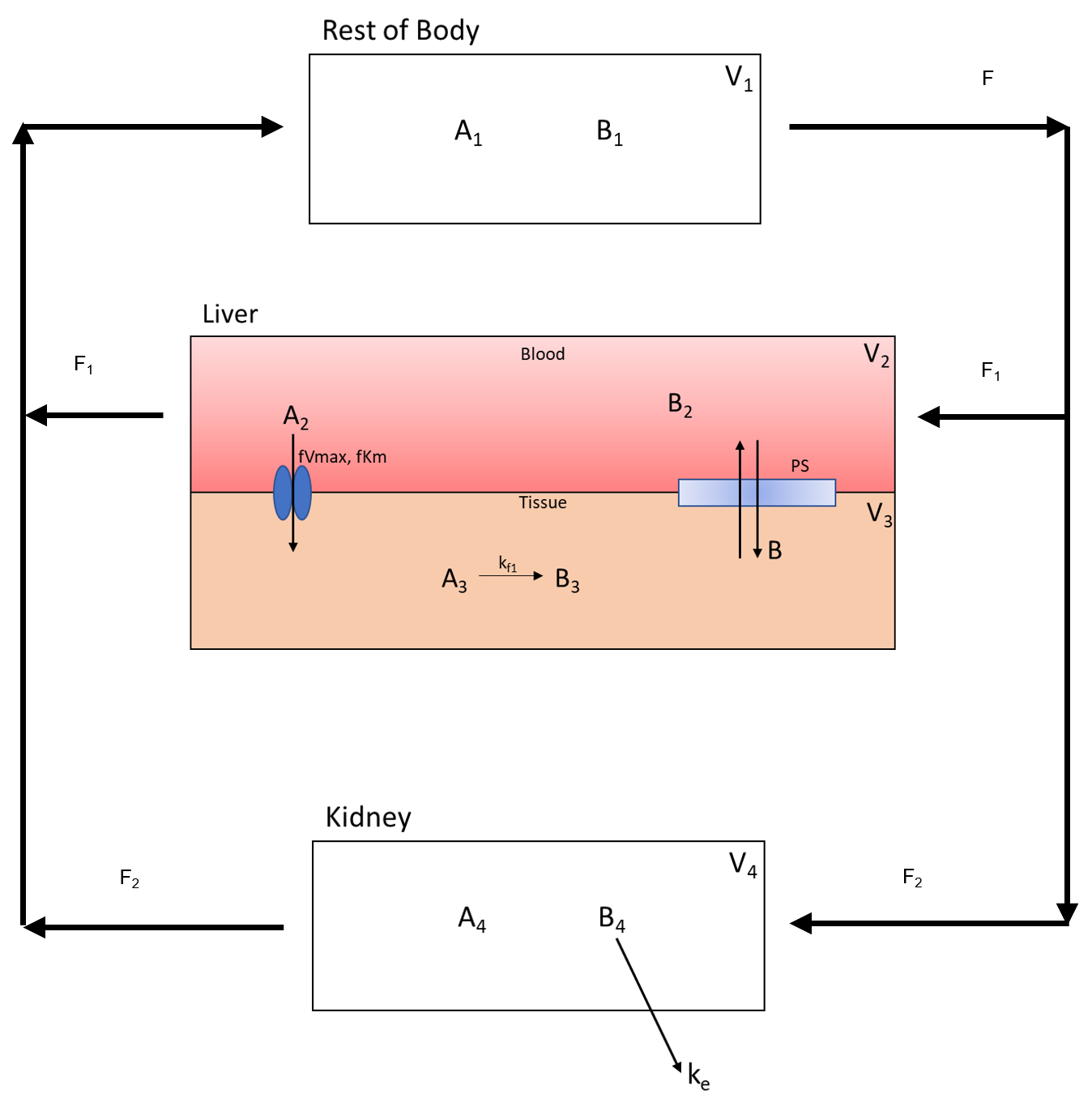


The following are the parameter and concentration tables for the model:


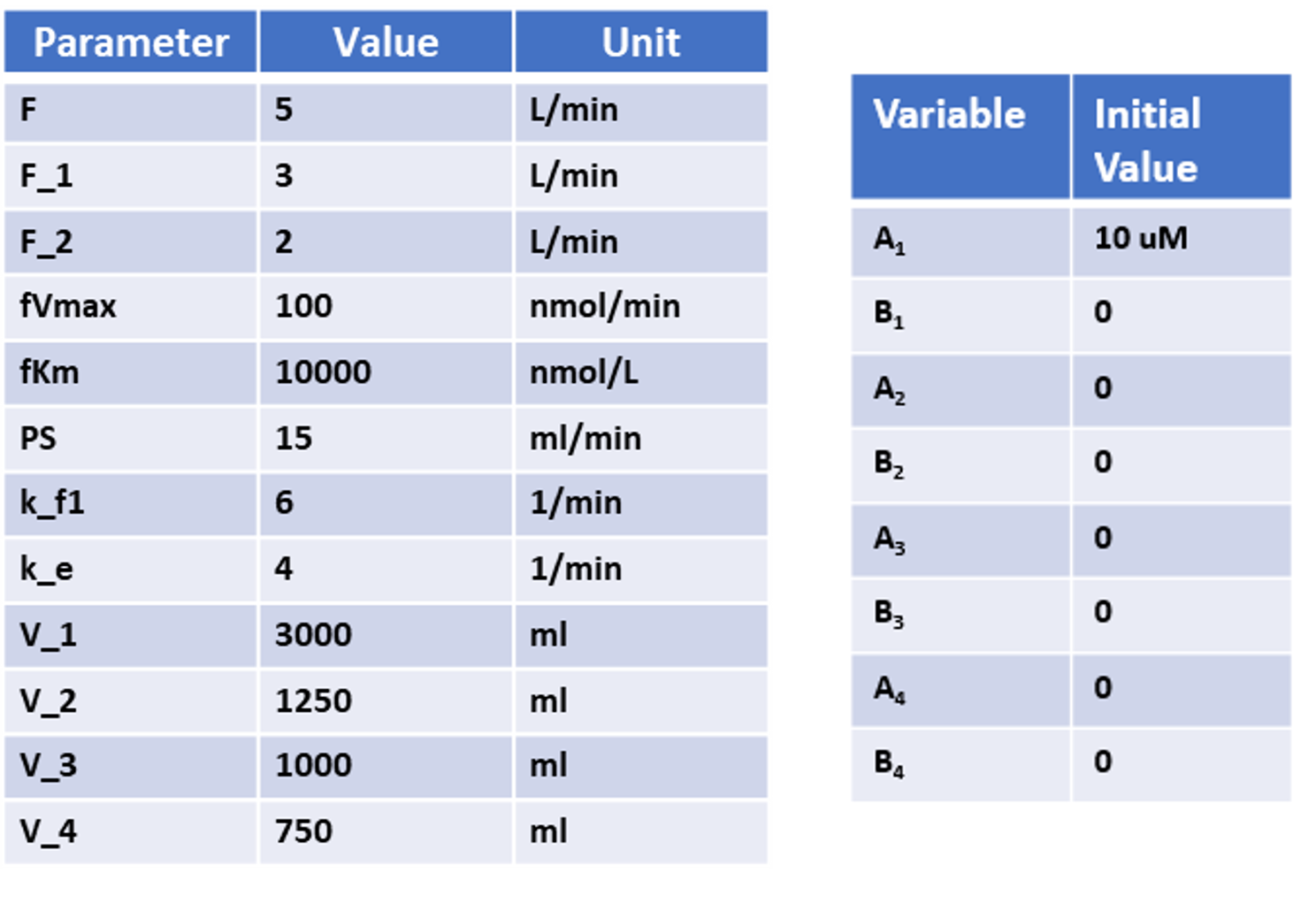


In the following sections of this tutorial, we will design the above multi compartment method.

The contents focus on:

1. Adding multiple compartments.
2. Adding variables between compartments.
3. Properly assigning flows between compartments
4. Using transporters to pass species between compartments.

To use the online version of this application visit <https://biomodme.ctsi.mcw.edu/>. Your current model can be downloaded in the “Export” tab as an .RDS file and can be loaded in by opening the right sidebar of the application. The application can also be directly downloaded at <https://github.com/MCWComputationalBiologyLab/BioModME> and opened using the R programming language using standard Shiny app protocol.

## Constructing Model

This section will cover how to build and construct a multi compartment model with a focus on input and outputs from different compartments.

### Create Compartments

We will start by setting up our four compartments in this model: Rest_Of_Body, Liver_Blood, Liver_Tissue, Kidney.

Steps:

1. Go to the **Compartments** box in the **Create Model** tab.
2. Press the “**+**” button three times to add three compartments.
3. Change the Names of the compartments in the table to **Rest_Of_Body**, **Liver_Blood**, **Liver_Tissue**, and **Kidney**.
4. Volume names are autogenerated with numerical numbering.
5. Change the volume value and units to match parameter table (these values can also be changed in the parameter table for the model).

The result should be the following table:


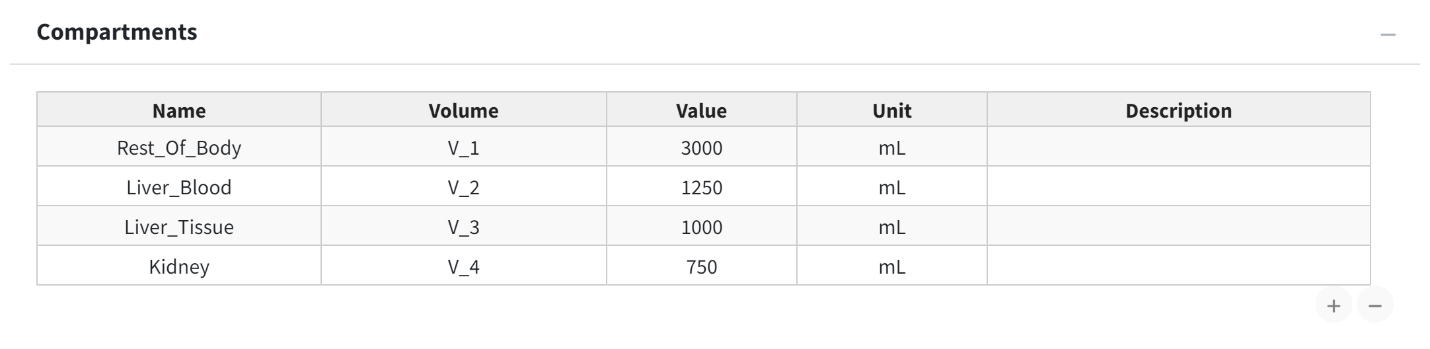


### Define Variables

There are two variables (**A** & **B**) to add to all four compartments. We will make use of the “Add To All Compartments” option to quickly add the same species to all compartments.

Steps:

1. Select the “Add To All Compartments” checkbox.

-
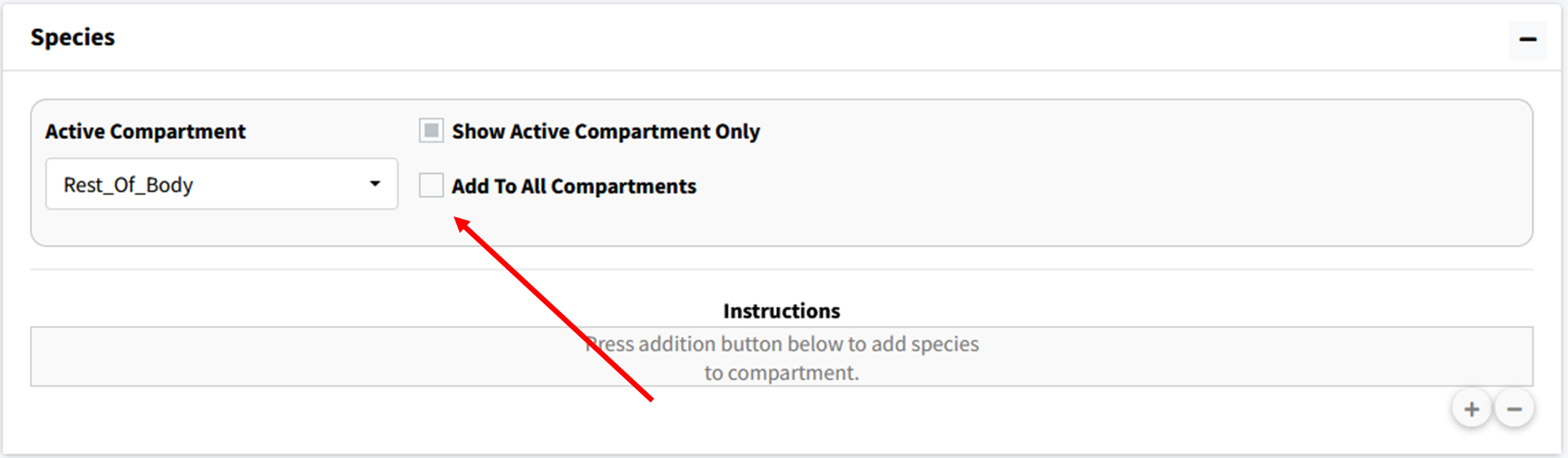


1. Press the “**+**” button to generate a popup.
2. In Popup type “A B” separated by a space. Check “Numerical” instead of “Compartment Name” and press the add button. This will add the variables **A_1**, **A_2**, **A_3**, **A_4**, **B_1**, **B_2**, **B_3**, and **B_4** to the model.

-
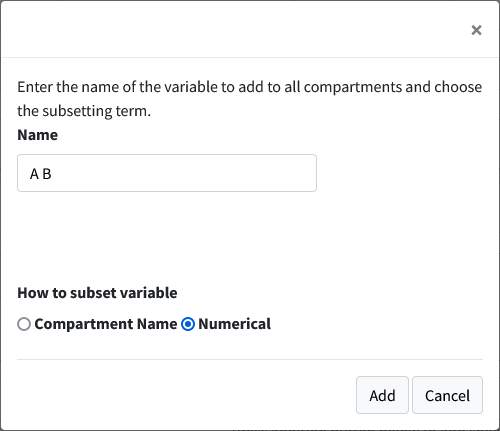


1. Unselect the “Show Active Compartment Only Checkmark” to see all the species in the model.

- Res
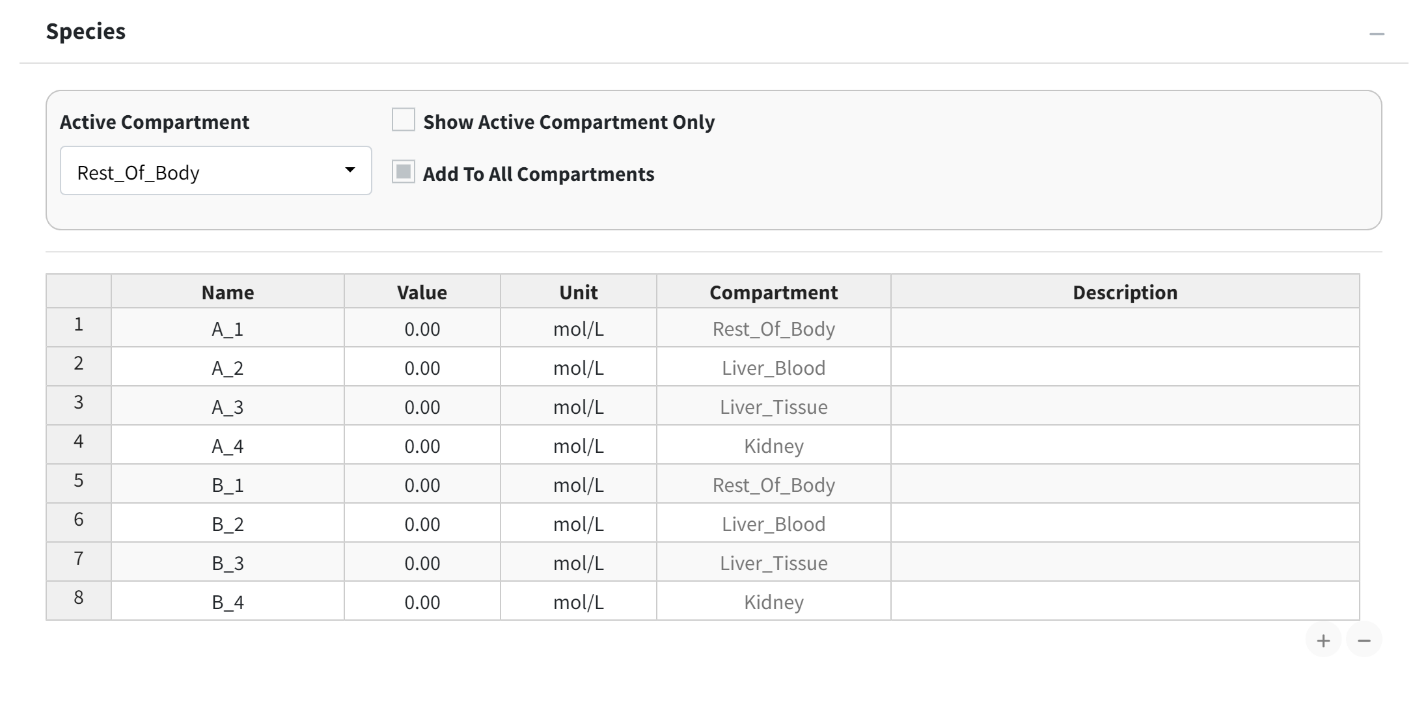


1. Change the concentration of **A_1** to match the initial condition table.

-
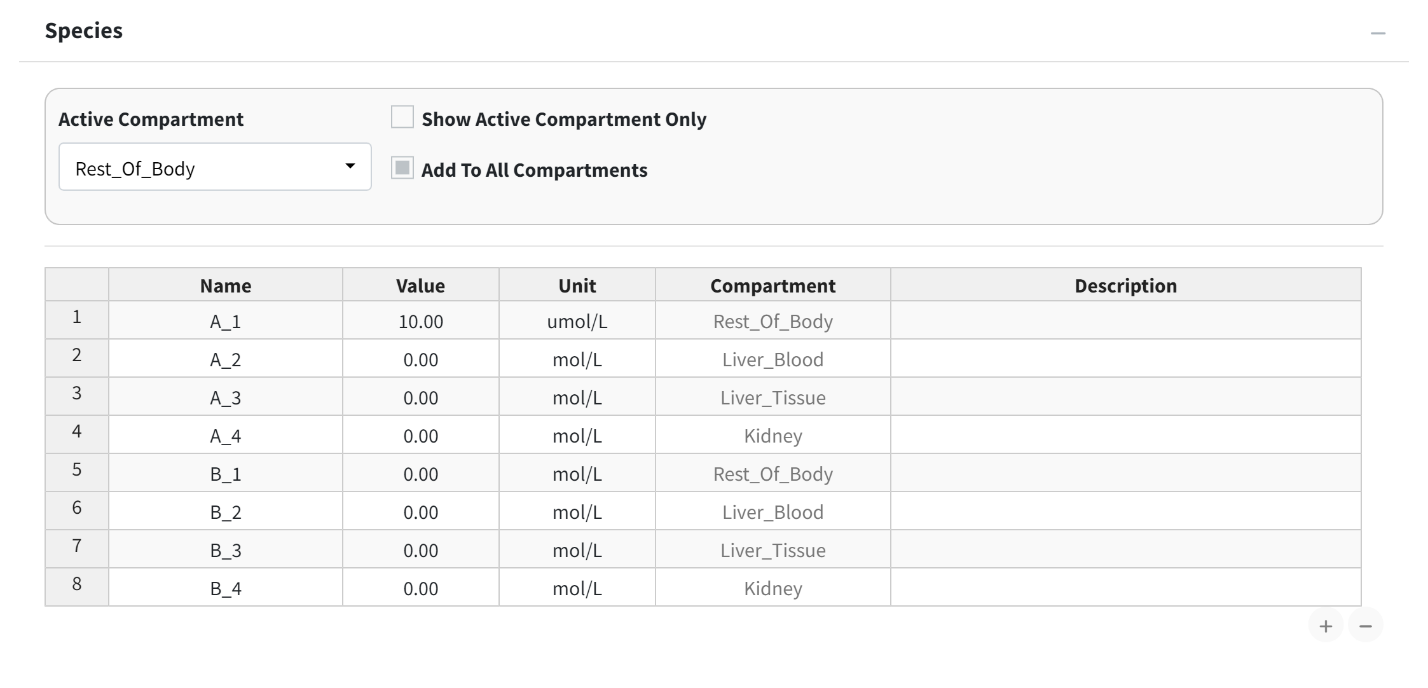


### Add Equations

This tutorial model has only one reaction: the conversion of A to B (**A3** & **B3**) in the liver tissue.


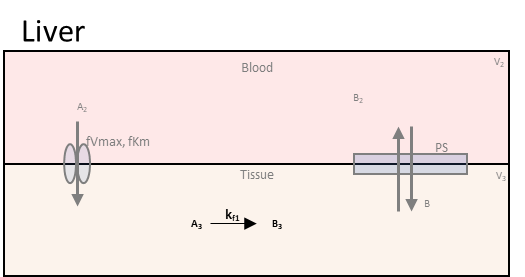


Steps:

1. Scroll down to the **Reactions** box in the **Create Model** tab. Press the addition button below the equation table.
2. Change the **Active Compartment** to “Liver_Tissue”.
3. Make sure **Mass Action** is selected as the **Law** for this reaction. Change the **Reaction Direction** to “Forward” for this is a one way reaction.
4. This reaction has one reactant and one product. Make sure **Reactant 1** is **A_3** and **Product 1** is **B_3**. Make the **Forward Rate Constant** to **k_f1**, if not already. You can set the parameter value here or later on the parameter table. For this tutorial most values will be set on the parameter table.
5. Press the **Add Equation** button.


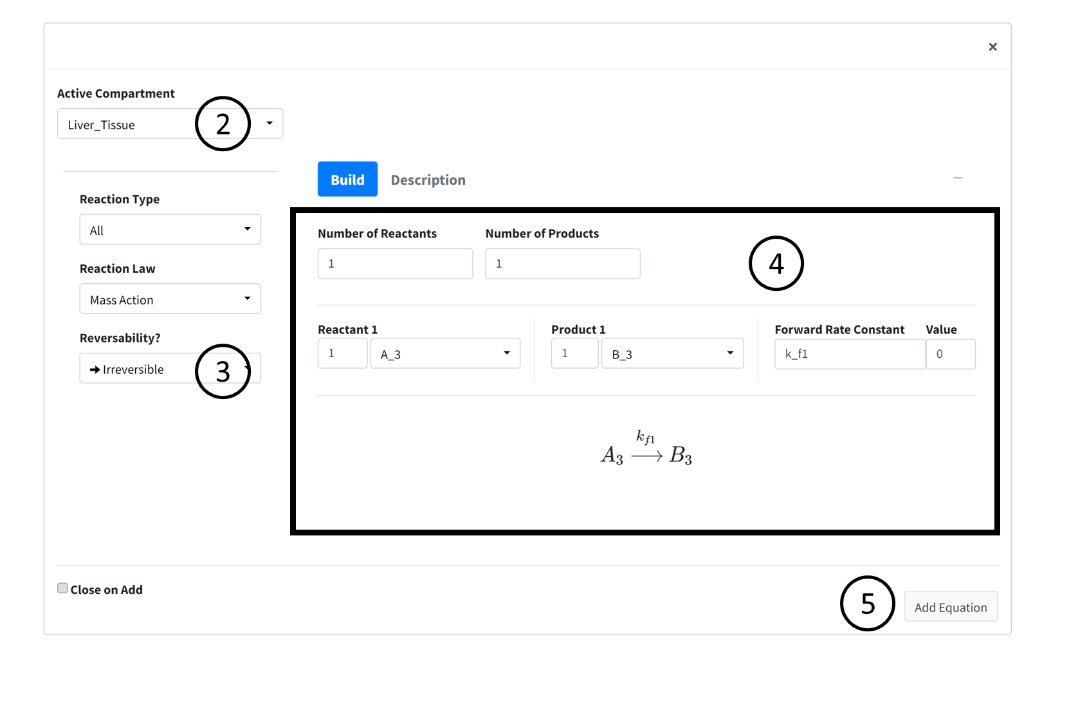


### Add Compartment Inputs/Outputs

In this section of the tutorial, we will cover how to add flows, inputs, and outputs of compartments to the model. Begin by scrolling to the **Input/Output** box on the **Create Model** tab. Press the addition button to begin adding the below flows between species information. Again, here is the overall flow model:


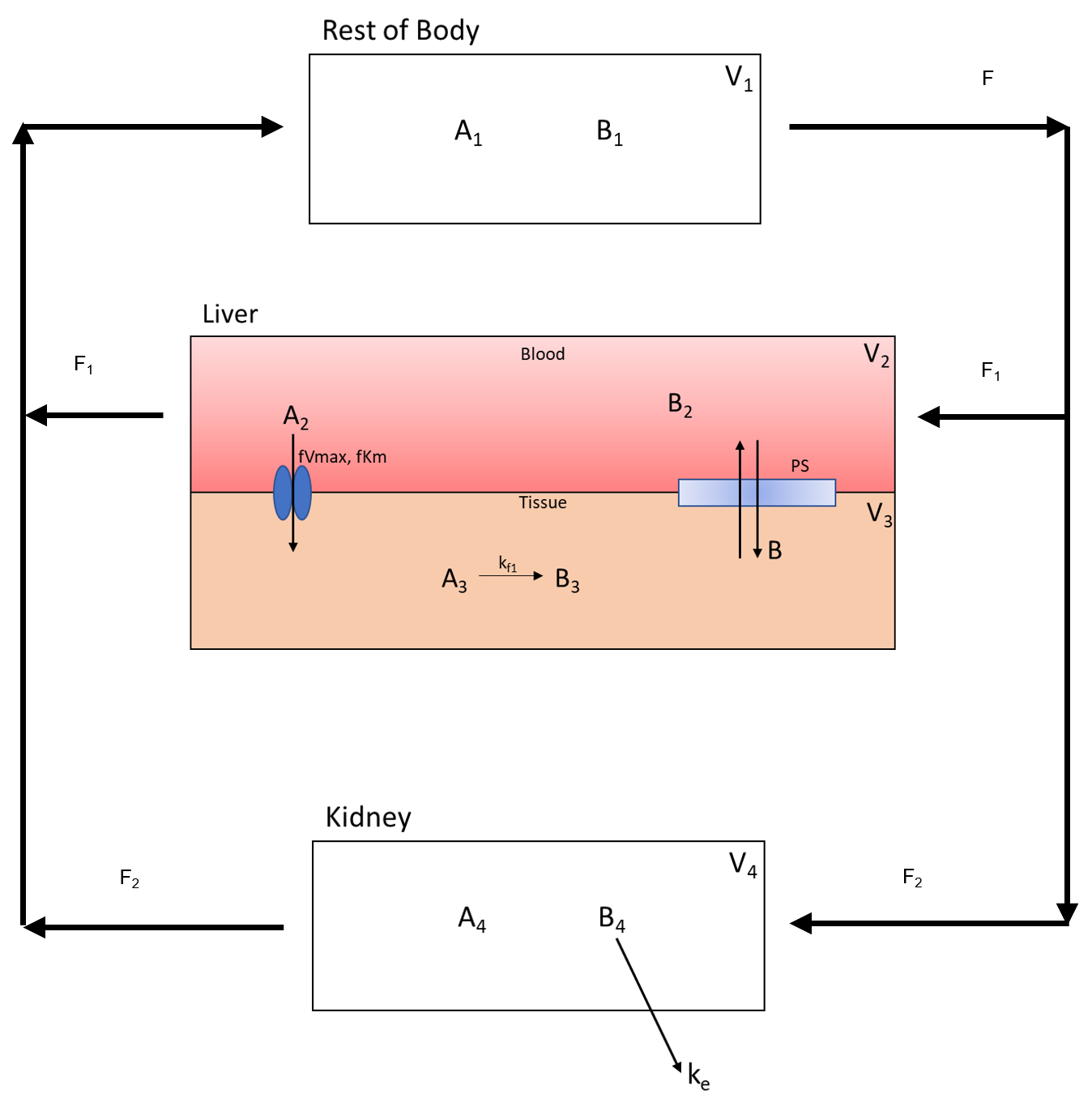


In each subsequent section we break down the flows into individual components.

#### 1. Flow Out of Body


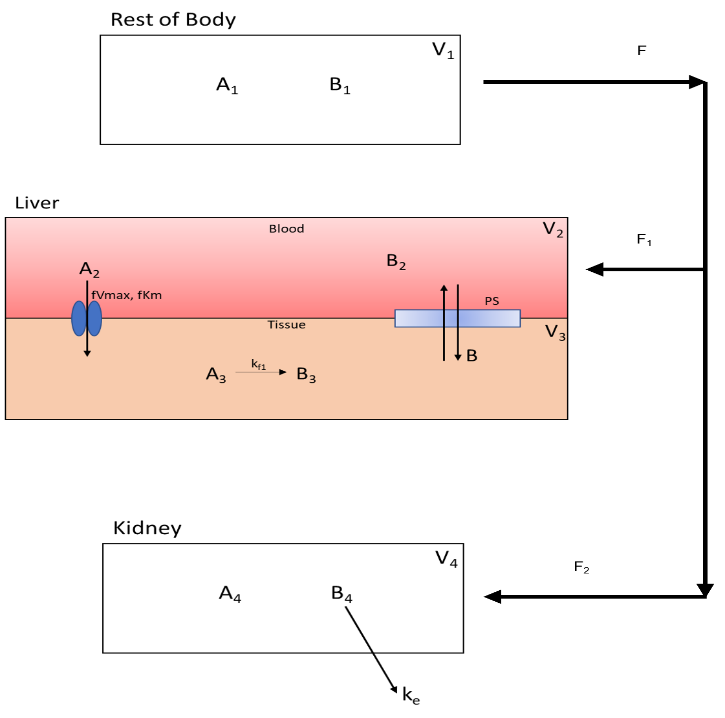
The flow out of the **Rest_Of_Body** is split into two flow rates as it moves species **A** and **B** to the **Liver_Blood** and **Kidney**. Below is the part of the flow diagram we will be entering:

1. In **Options** select **Flow Between Compartments**. This is used to account for flows leaving one compartment and entering one or more compartments.
2. Select the **Split Flow** checkbox. This option is selected when one outflow is being split into multiple flows. Keep **Number of Splits** to **2** as we are splitting the flow to two compartments.
3. Set the information for the compartment the flow is leaving from. It has the following specifications:
   - **Flow_Out_of**: Rest_Of_Body
   - **Species Out**: A_1
   - **Flow Variable**: F
   - **Flow Value**: 5

- Current specifications need to have species in flow entered one at a time. Meaning a new flow will be added for species **B**. With all parameters, values can be changed later in the parameter section.

1. Set the information from the first split flow into **Liver_Blood**. Enter the following:
   - **Flow_Out_of**: Liver_Blood
   - **Species Out**: A_2
   - **Flow Variable**: F_1
   - **Flow Value**: 3
2. Set the information from the second split flow into **Kidney**. Enter the following:
   - **Flow_Out_of**: Kidney
   - **Species Out**: A_4
   - **Flow Variable**: F_2
   - **Flow Value**: 2
3. Press the **Add** button to add this flow. You can select the **Keep Active** checkbox on the bottom of the page to prevent this page from closing on pressing the add button.

-
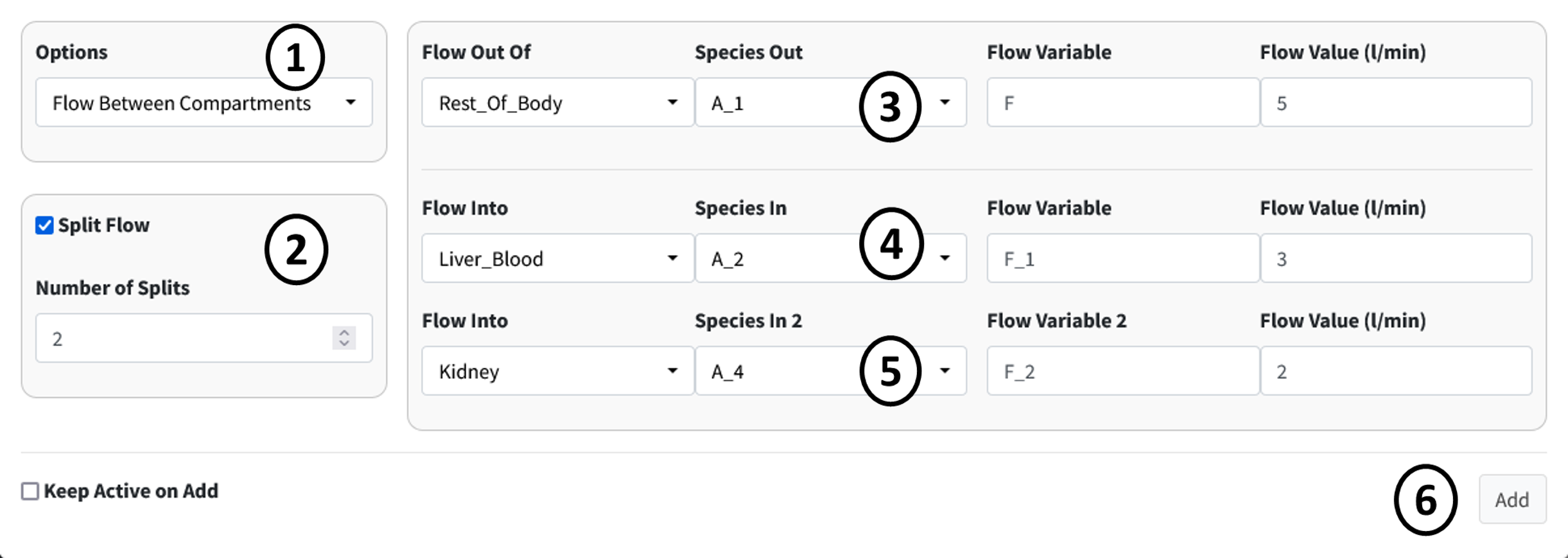


1. Repeat the previous steps for species **B**. This should involve only changing the **Species** dropdowns.

-
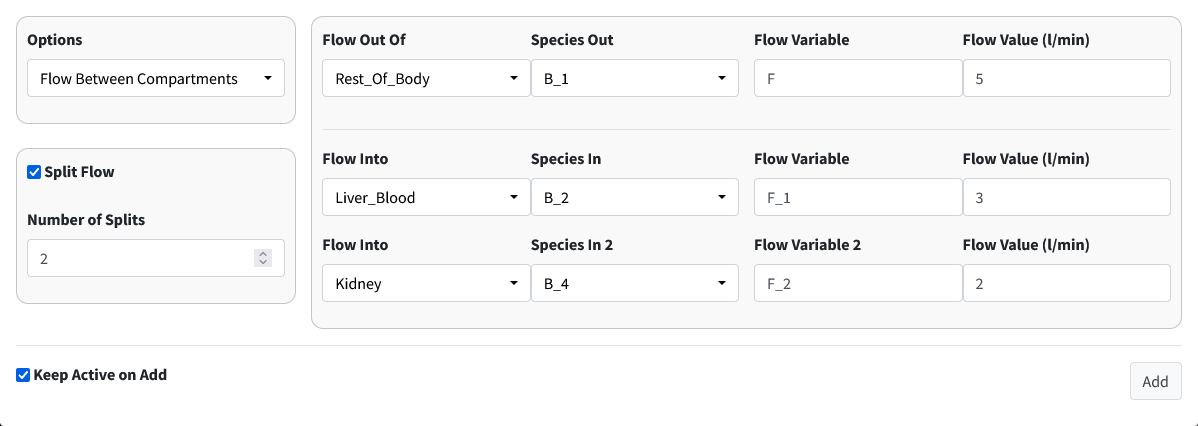


Note

When adding **B**, we are reusing the flow variables **F**, **F_1**, and **F_2**. BioModME allows multiple saving of parameters but will check that the parameters have the same base unit. If not, the parameter will be rejected. The value for the parameter will overwrite on each new overwrite of the parameter.

#### 2. Flow Back: Liver to Body

The flow back from the liver to the rest of the body looks like:


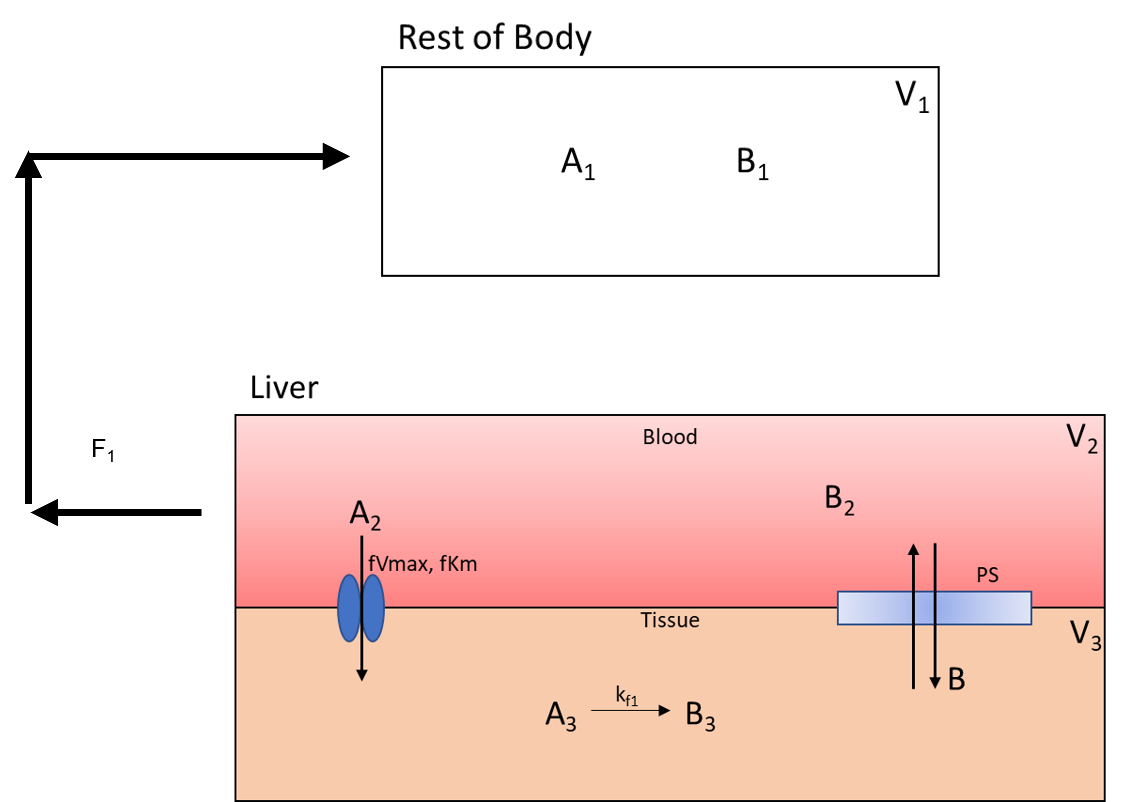


This is like the previous step but with the compartments switched around.

Steps:

1. Make sure **Options** is still on **Flow Between Compartments**.
2. Turn off **Split Flow**.
3. Set the information for the compartment the flow is leaving from. It has the following specifications:
   - **Flow Out Of**: Liver_Blood
   - **Species Out**: A_2
   - **Flow Variable**: F_1
   - **Flow Value**: 3
4. Set the information for the compartment the flow is entering. It has the following specifications:
   - **Flow Into**: Rest_Of_Body
   - **Species In**: A_1
5. Press the **Add** button to add this flow.

-
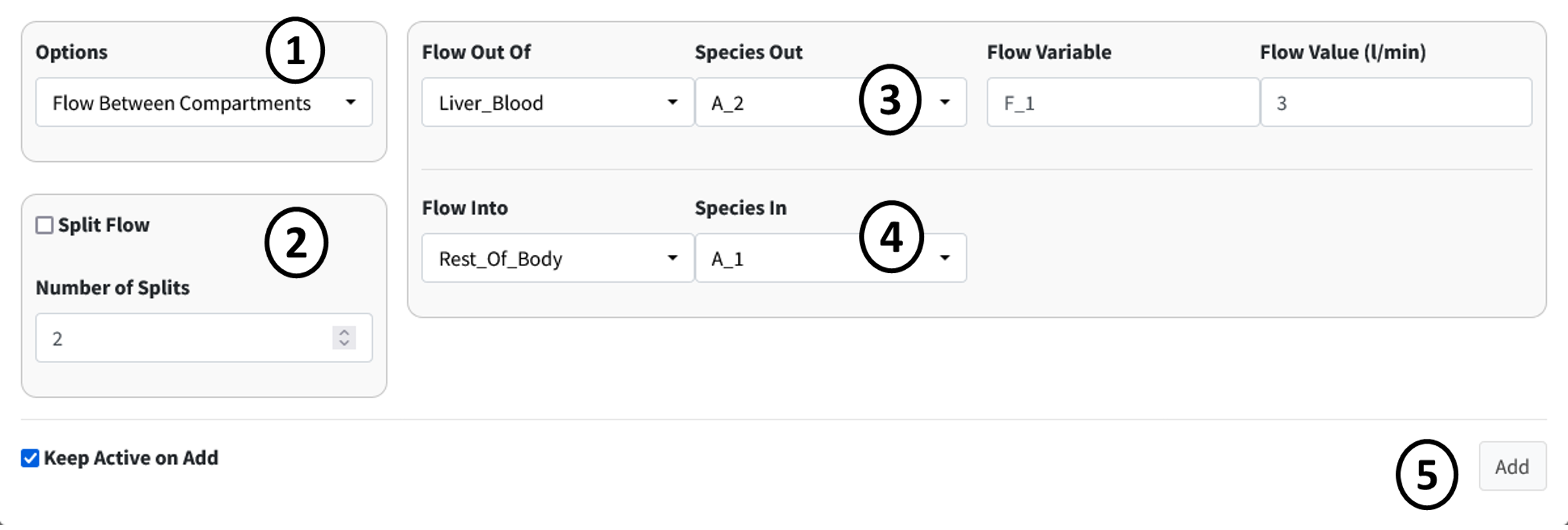


1. Repeat the previous steps for species **B**. This should involve only changing the **Species** dropdowns.

-
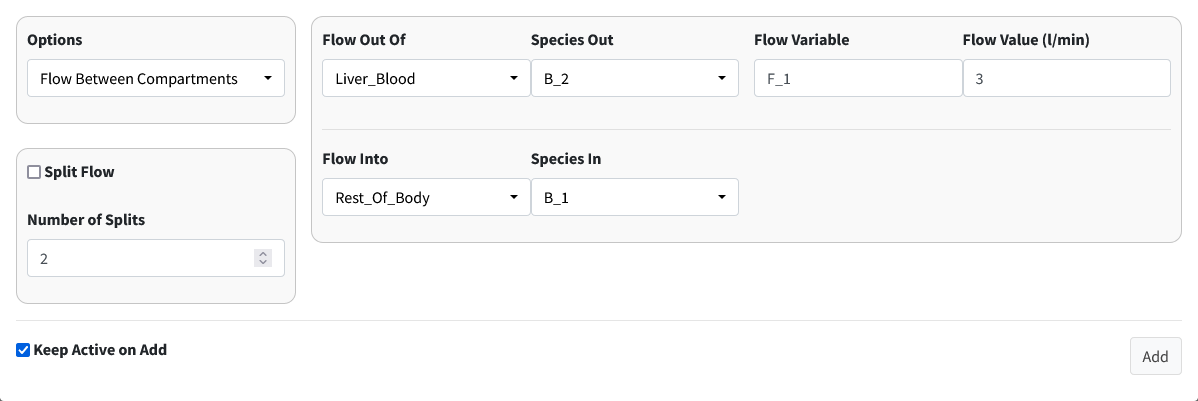


#### 3. Flow Back: Kidney to Body

The flow back from the kidney to the rest of the body is:


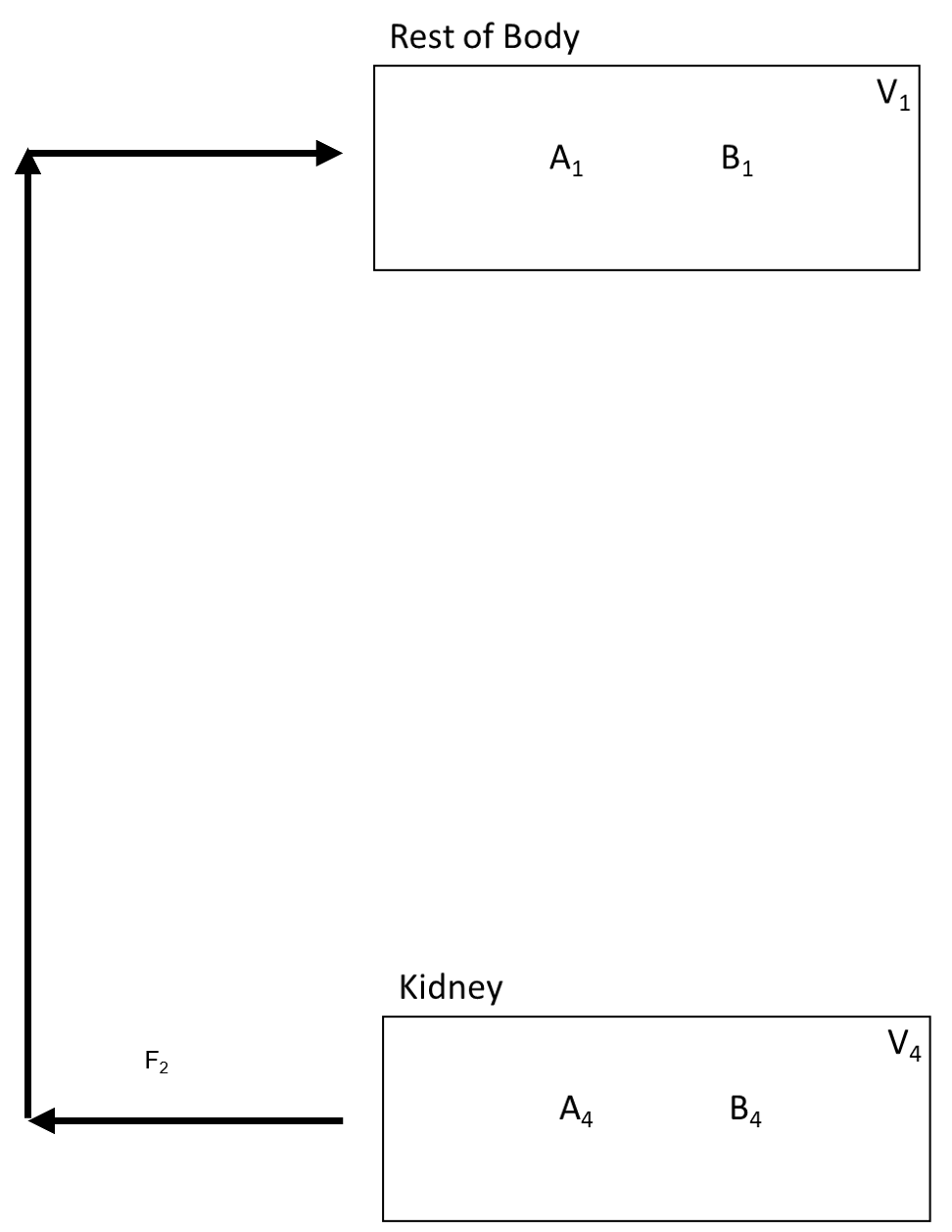


Steps:

1. Make sure **Options** is still on **Flow Between Compartments**.
2. Check that **Split Flow** is off.
3. Set the information for the compartment the flow is leaving from. It has the following specifications:
   - **Flow Out Of**: Kidney
   - **Species Out**: A_4
   - **Flow Variable**: F_2
   - **Flow Value**: 2
4. Set the information for the compartment the flow is entering. It has the following specifications:
   - **Flow Into**: Rest_Of_Body
   - **Species In**: A_1
5. Press the **Add** button to add this flow.

-
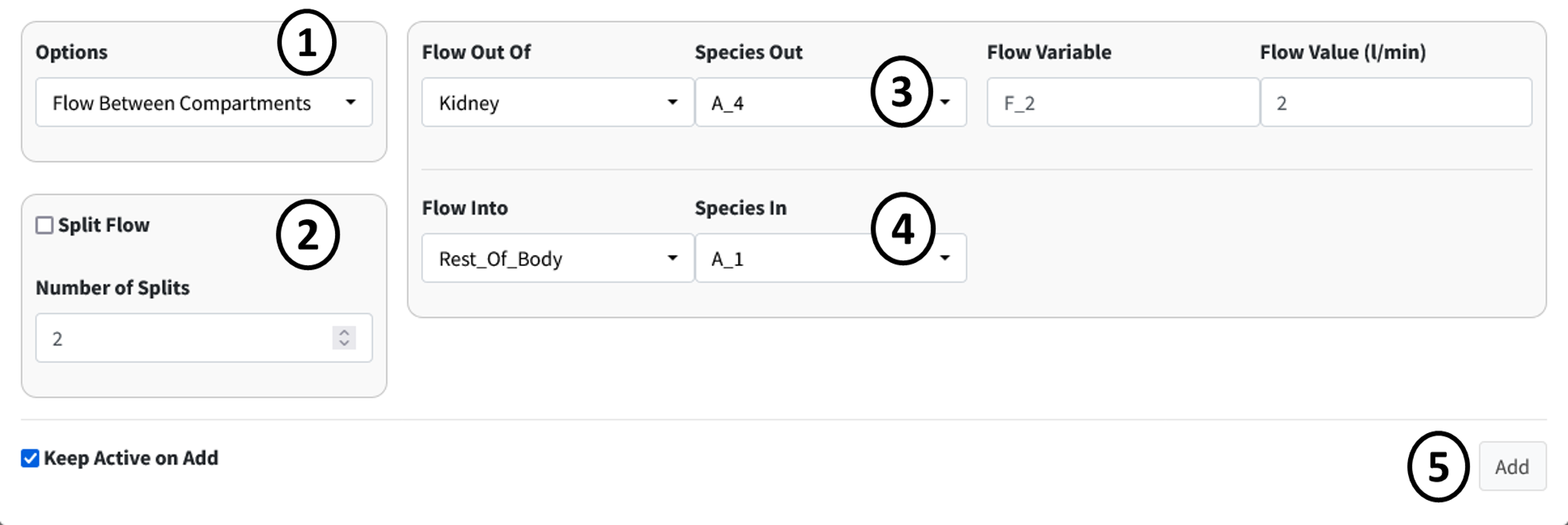


1. Repeat the previous steps for species **B**. This should involve only changing the **Species** dropdowns.

-
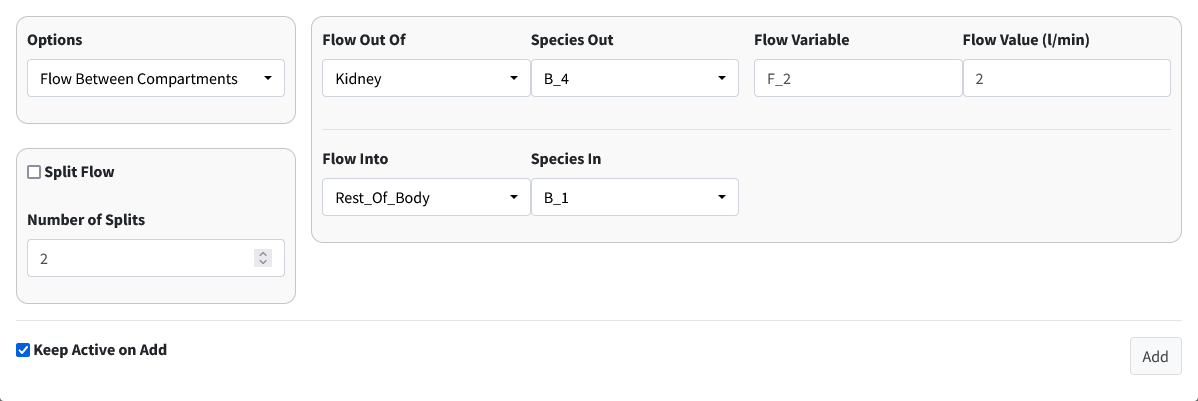


#### 4. Clearance of A From Kidney

Drug A is excreted from the kidney at a constant rate. The isolated process is shown below:


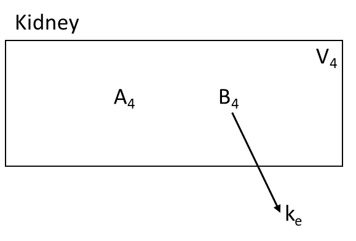


Steps:

1. Select **Clearance** in the **Options** dropdown.
2. Enter the following information in the main box:
   - **Compartment**: Kidney
   - **Species**: B_4
   - **Rate**: k_e
3. Press the **Add** button to add the clearance of B from the kidney.


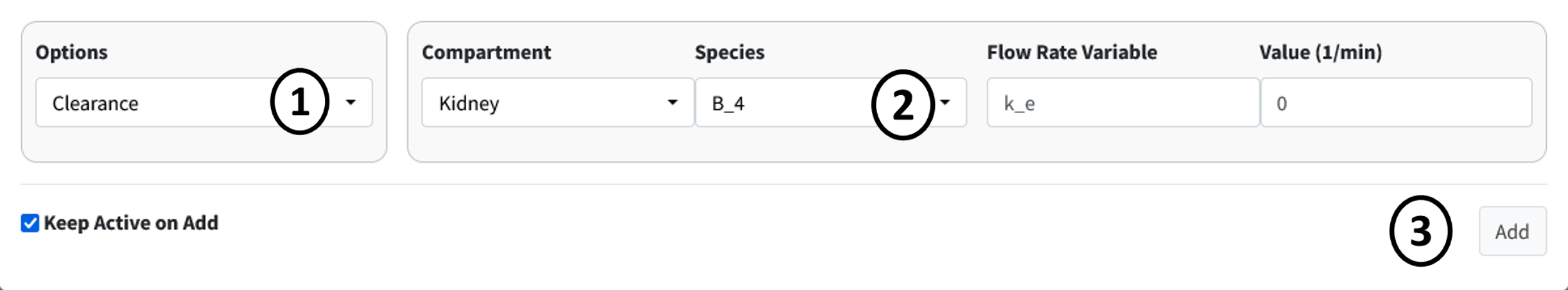


#### 5. Facilitated Diffusion of A

The next two seconds will look at diffusion processes from the liver blood to the liver tissue and back. Below is the facilitated diffusion of molecule **A** from the liver blood to the liver tissue.


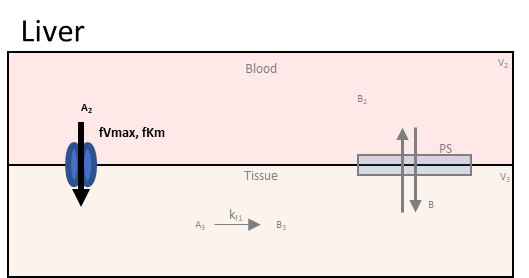


Steps:

1. Select **Facilitated Diffusion** in the **Options** dropdown.
2. Enter the following information in the first row of the main box:
   - **From Compartment**: Liver_Blood
   - **From Species**: A_2
   - **Vmax**: fVmax_1
   - **Km**: fKm_1
3. Enter the following information in the second row of the main box:
   - **To Compartment**: Liver_Tissue
   - **From Species**: A_3
4. Press the **Add** button to add this facilitated diffusion flow to the model.


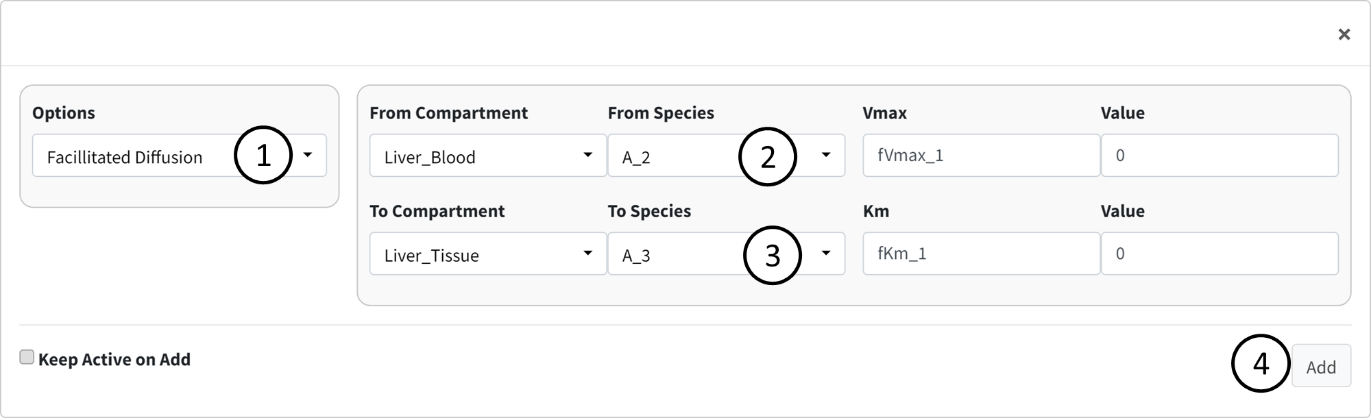


#### 6. Simple Diffusion of B


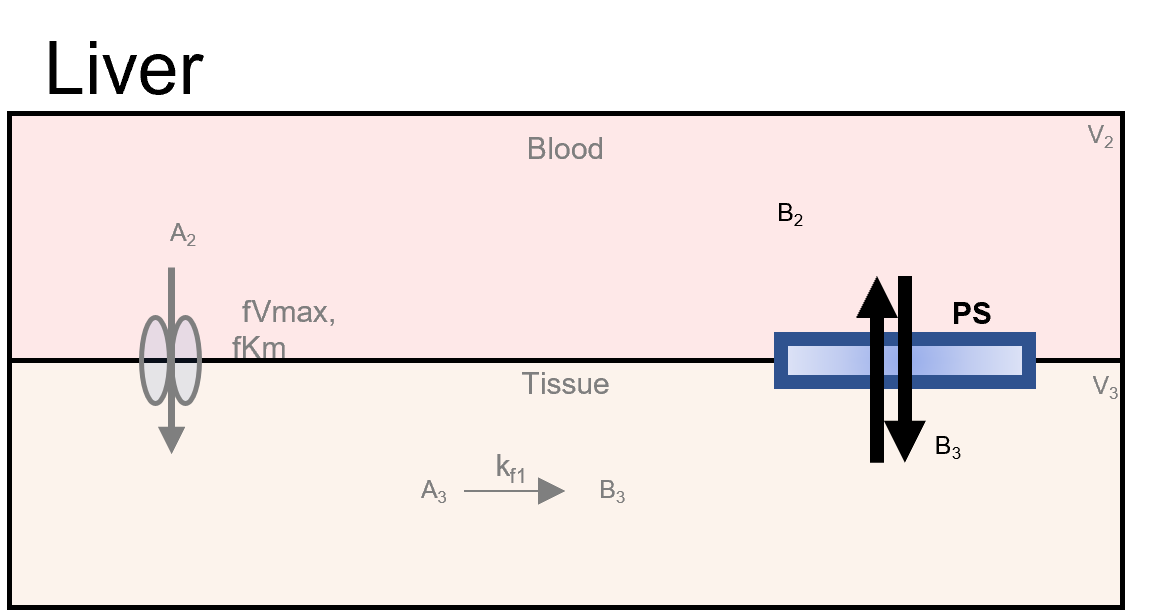


Steps:

1. Select **Simple Diffusion** in the **Options** dropdown.
2. Enter the following information in the first row of the main box:
   - **Compartment**: Liver_Blood
   - **Species**: B_2
   - **Permeability surface area product**: PS
3. Enter the following information in the second row of the main box:
   - **Compartment**: Liver_Tissue
   - **Species**: B_3
4.
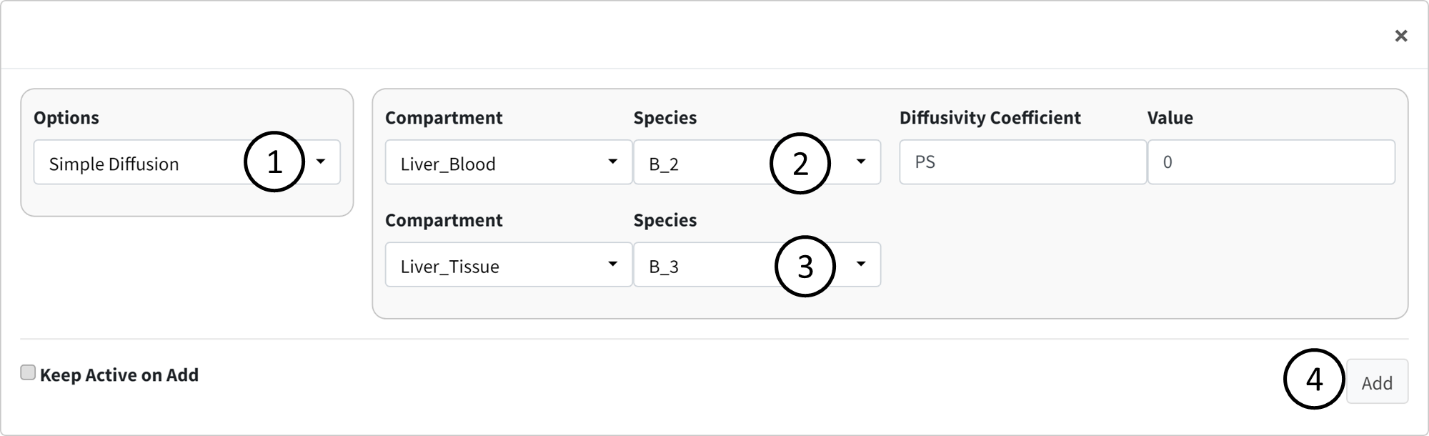
Press the **Add** button to add this simple diffusion flow to the model.

Note

The order of entered compartments and species does not matter for simple diffusion.

This should be the last term entered in the Input/Output box. There should be nine terms in the results table.

### Parameters

Navigate to the next box: **Parameters**.

Below is the generated parameter table that has been created so far by the above entered information. All descriptions are pregenerated based on where the parameter originated. Like other tables, all values are editable.


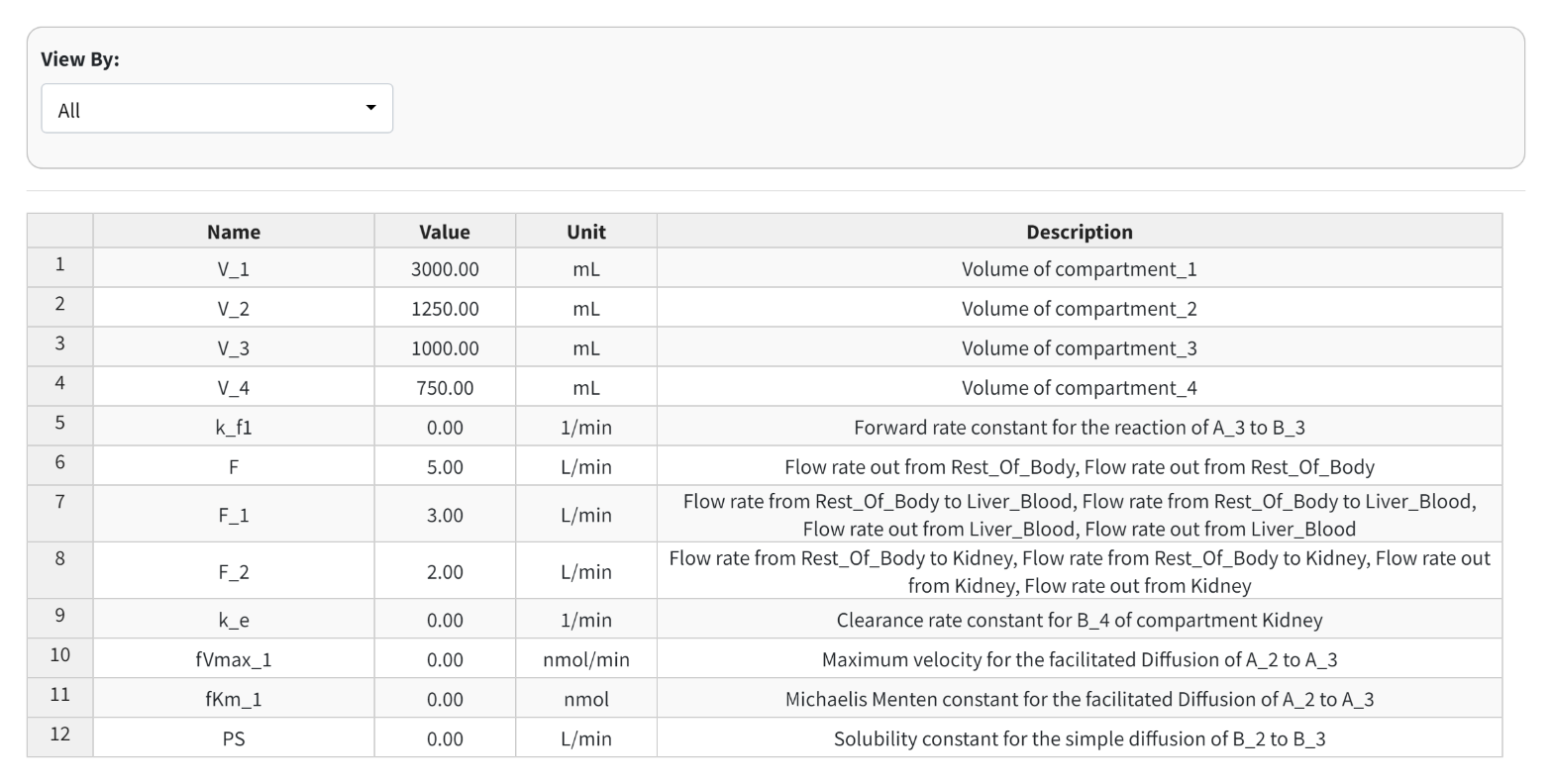


The parameter values for the model are as follows:


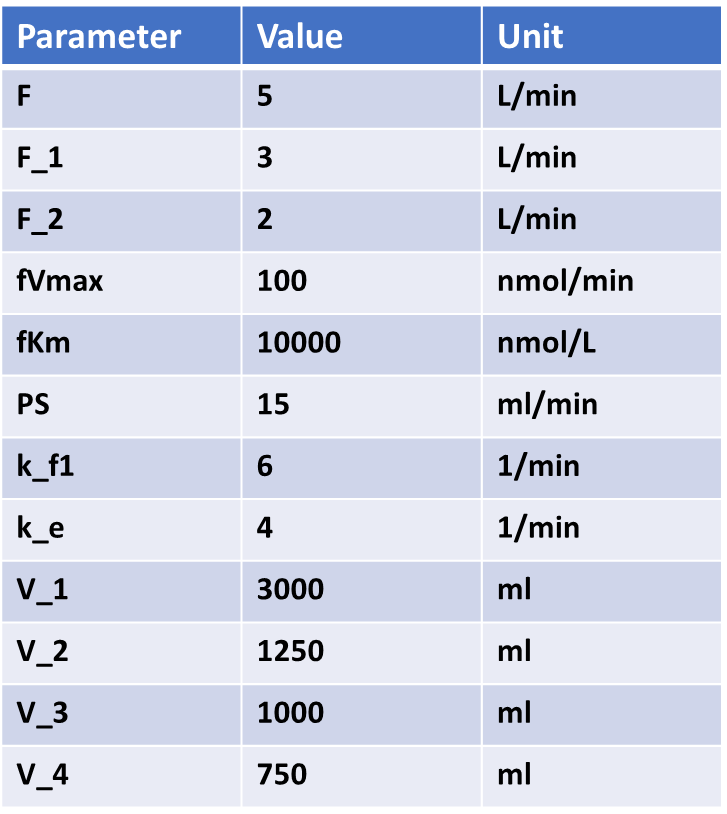


The below figure shows the parameter table with the values entered.


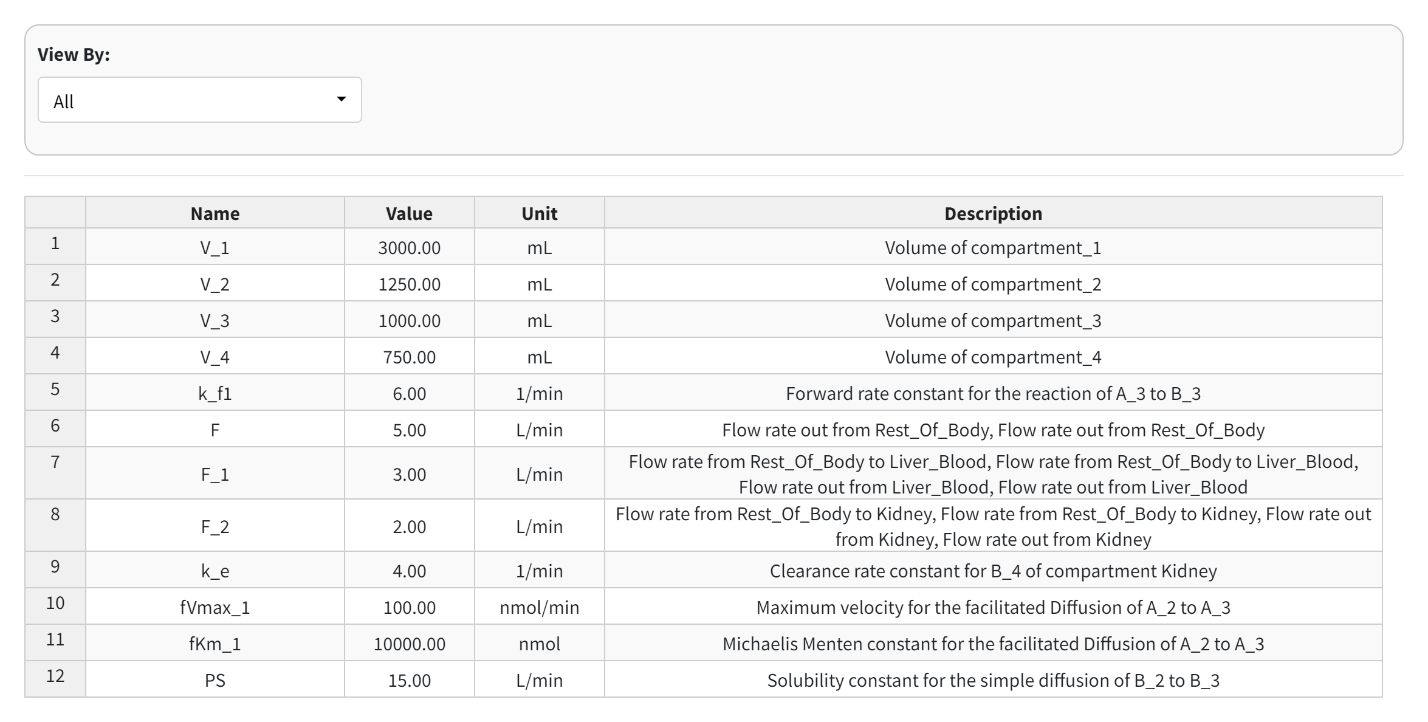


Note

In the Figure above, we did not keep the units of our parameters consistent. In some cases, ml was used and in other liters was used. This is okay. The application converts all units to a “base” unit in the background and performs all calculations at that base level.

### Differential Equations

Navigate to the final box **“Differential Equations”**.

This tab will begin with all species being equal to “0” and gets auto-generated based on reactions added. The "Generate" button will refresh the equations, if for any reason they need to be refreshed.


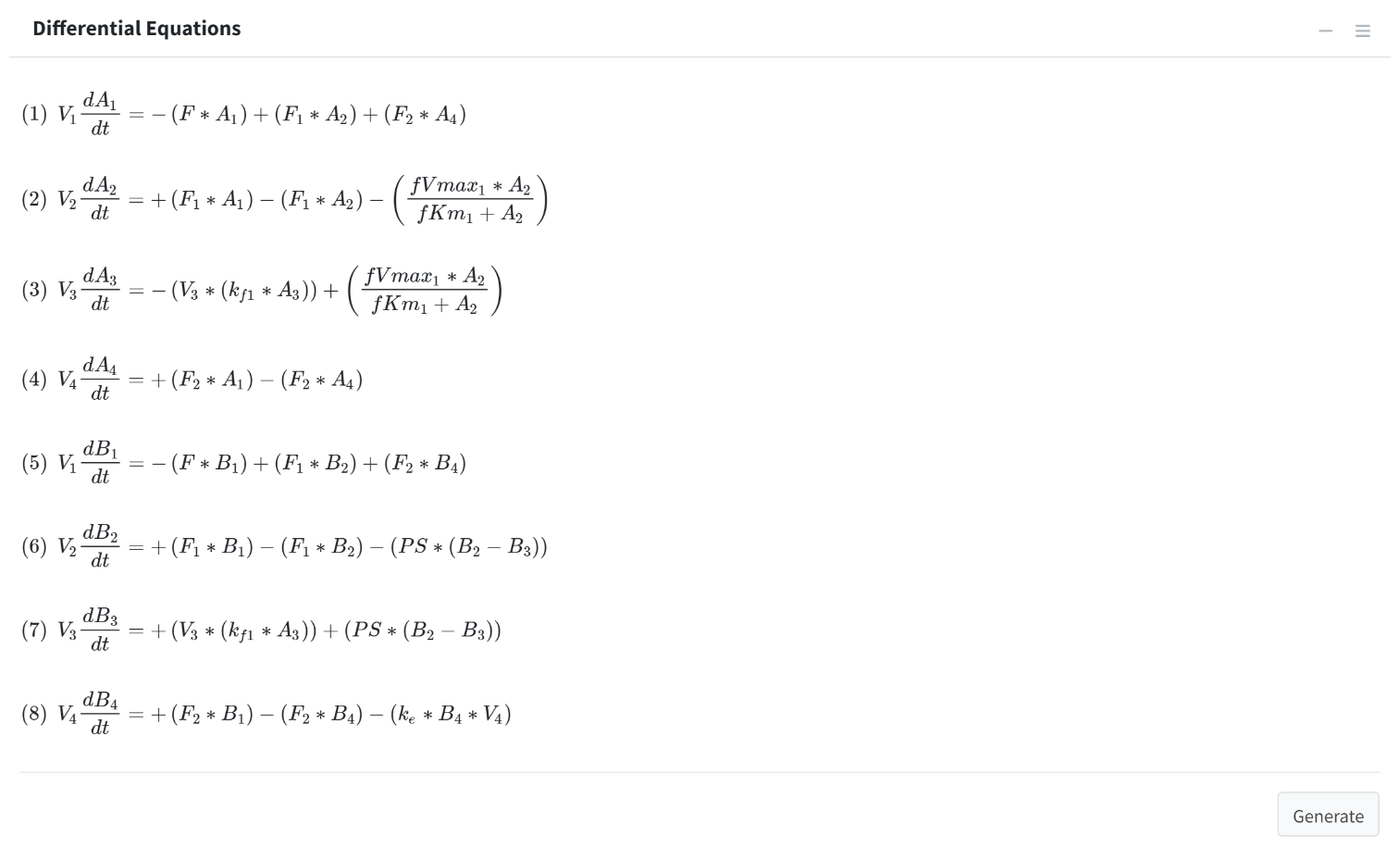


Note

The right sidebar of the “System of Differential Equations” box has an option for viewing the differential equations including turning off the math rendering and showing each derivation line as a newline for each differential equation

## Results

In this section, we will cover executing the model and its different outputs.

### Solve Model

Navigate to the “Execute Model” tab on the lefthand tabbar. This tab solves our model and provides us with the options to customize the solver. For this model, we will just solve using the standard options:

1. Enter the following times: Starting = 0, End = 5, Step = 0.001, Unit = hr. This generates the model to be solved and integrated at time points 0, 0.001, 0.002…..5.998, 5.999, 6.
2. Press the “Run Solver” button.
3. An output table should be generated with the concentration of each species along each time step of the solved model.

You can download this solved model data to a csv file in the download tab on this page. Post processing is not used in this tutorial.


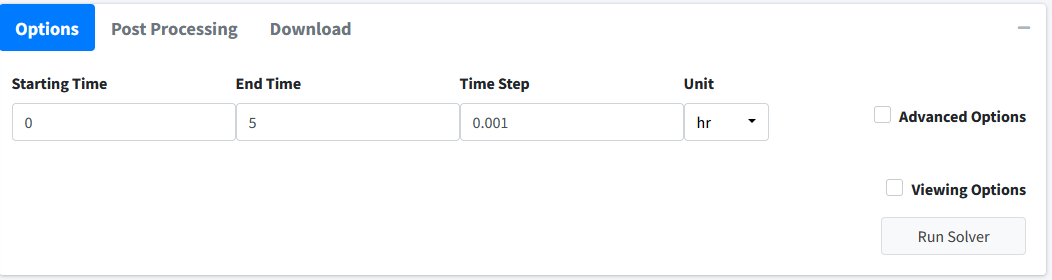


Note

BioModME is set by default to show the results table rounded to 3 digits. Most terms in these results at the beginning will round to 0. To change this, check the **Viewing Options** box and turn off the **Round** option or turn on the **Scientific Notation** option.

Below is how the table for this model should look after the initial solve:


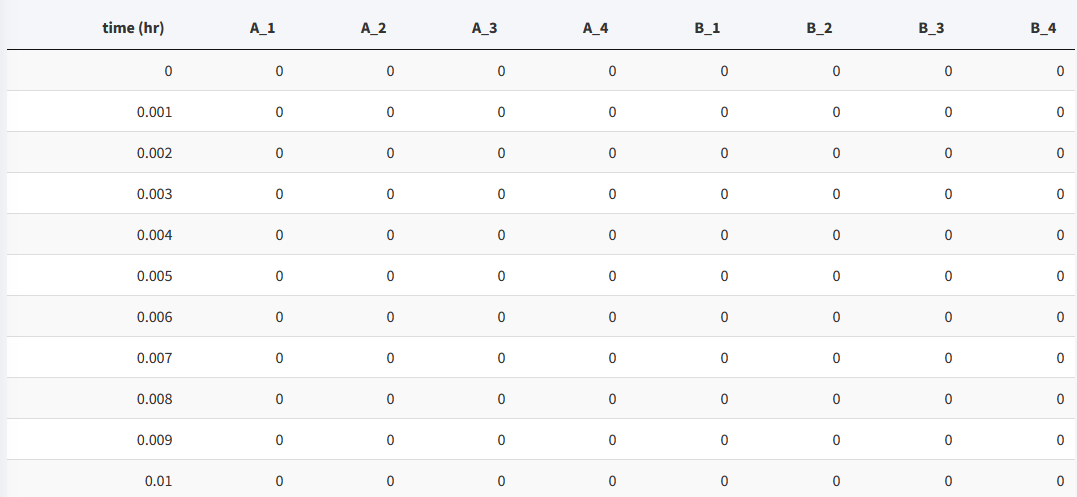


To see the values for the model in a good form, we will change the viewing options:

1. Click the **Viewing Options** Checkbox.
2. Change Results Units to **umol**.


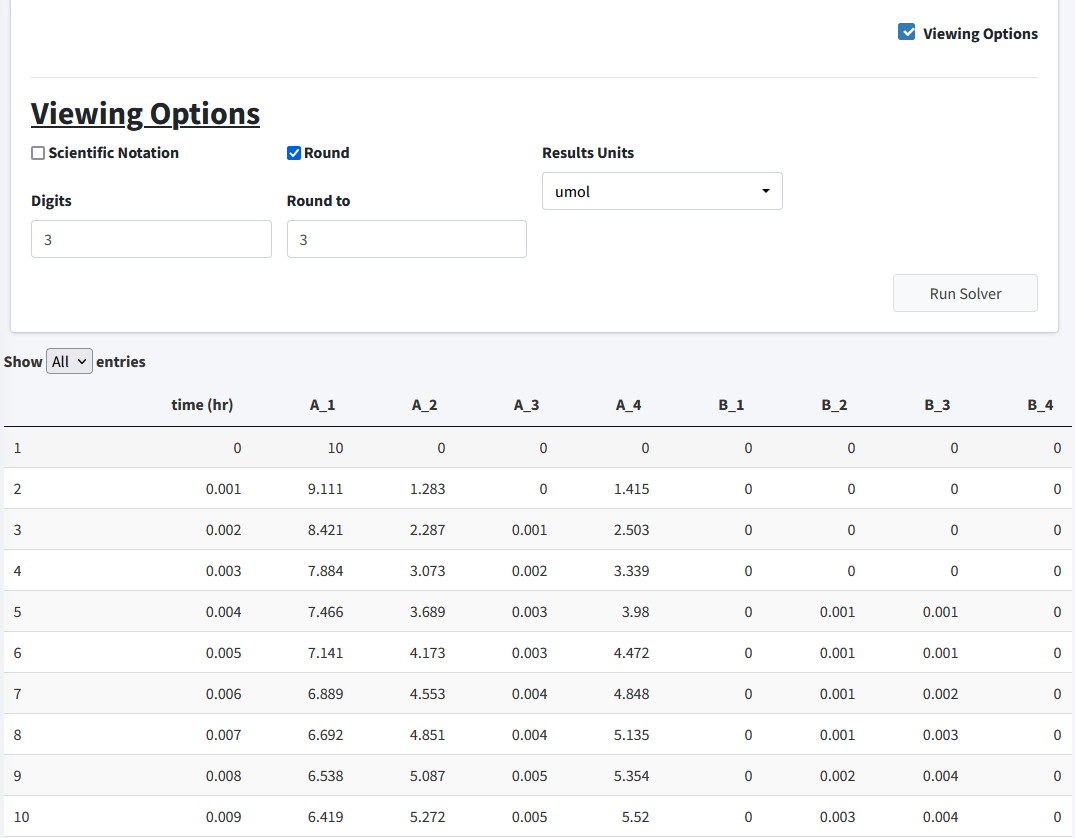


In this section, we will examine the plotting of this model.

## Visualization

Move to the **Visualization** tab. The starting plot from the model should look like the following:

For this problem we want to examine how the concentration of **A** varies throughout the bloodstream (**A_1**, **A_2**, **A_4**). Click the **Variable** dropdown and remove all necessary variables:

The resulting plot should look like:

# Parameter Estimation Tutorial

This is a basic parameter estimation tutorial. To download the example data used in this tutorial go to <https://github.com/Jwomack7512-bio/BobTheBuilder/blob/main/shared_files/Nicotine_cigarette.csv>.

We assume you have basic knowledge of creating a model and will skip showing every step of that process. Please see the application documentation for details on the solver and options available to use with the parameter estimation solver.

We will construct the following simple three compartment model:

In the above diagram, we model the concentration of a drug, Nicotine (**C**), as it moves from the alveoli of the lungs, through the plasma of the body, and a compartment summarizing various tissues of the body. We assume Nicotine flows out of the alveoli with flow rate (**F_1**), into the plasma of the body. From there, Nicotine diffuses to various tissues of the body through simple diffusion (**PS**) and is also removed from the plasma at a flow rate of **F_2**. In this model, we are unsure of the value of **PS** and will use parameter estimation to estimate the value from experimental data that found the concentration of nicotine in the plasma at various timepoints. The starting concentration of **C** in the alveoli is 1.2 mol/L.

## Build Model

To being building the model, we will add our three compartments with their corresponding volumes:

- Compartment: Alveoli, Volume = 1 L
- Compartment: Plasma, Volume = 7.8 L
- Compartment: Various_Tissues, Volume = 9.5 L

Next, we will add a concentration (**C**) variable for nicotine to each compartment and give an initial concentration of 1.2 mol/L to **C_1**.

Next, we need to add the compartment input/outputs. We have three to add:

1. Flow between Alveoli and Plasma


1. Flow out of Plasma


1. Simple diffusion between Plasma and Various_Tissues


With all our model components constructed, we need to assign parameter values. We do not know the value of PS but we will give the model a reasonable starting estimate (0.5 L/min).

Note:

The parameter table rounds to two decimal places. This is why F_2 shows as 0.08 in the table. If you double-click the value, it will show as the appropriate 0.078.

For reference, here are the solved differential equations:

Move to the **Execute Model** tab and enter the time for this model:

- Start Time = 0
- End Time = 70
- Time Step = 1
- Unit = min

The beginning of the results table should look like this:

## Visualize Model

Move to the **Visualization** tab. A starting plot should be generated that looks like:

We want to examine the concentration of Nicotine in plasma (**C_2**). Go into the **Variables** dropdown and remove variables **C_1** and **C_3**.

We should import our data for Nicotine in the plasma to overlay with the plot. Scroll below the plot to the box header that is titled **Import Data**. Browse your desktop for the nicotine dataset (which can be downloaded from our GitHub). Be sure to click the **Apply Overlay** checkbox to show the imported data. For importing data, the first row of the data is the headers. The first column should be time while the remaining columns should be your concentrations at those times. The header names have to match the model names for the data to be correctly plotted.

The resulting plot should look like the figure below:

In this overlayed plot, we can see our model has the general shape of our data but doesn’t quite mimic it well. We will run parameter estimation for **PS** to see if we can find a better fit.

## Perform Parameter Estimation

Go to the **Modeler’s Toolbox** Tab, subtab **Parameter Estimation**. Here, we will start by importing our data into this module.

Move to the next box on the page **Select Parameters**. Use the dropdown to select **PS**.

There are five columns in the generated table. They are as follows:

- Parameters - the selected values to estimate.
- Initial Guess - the starting value to begin parameter estimation at.
- Lower Bound - the lowest acceptable value this parameter can be.
- Upper Bound - the highest acceptable value this parameter can be.
- Calculated Value - the found value from parameter estimation after calculations.

Values for Lower and Upper bound can be left blank if no bounds want to be used. Here we use the following:

- Initial Guess - 0.50
- Lower Bound - 0
- Upper Bound - 1

Press the **Run** button and the program should output a value of approximately 0.10.

The next box **Estimation Results** will contain the model fit of the variable with its corresponding data along with the results of the iterations of the parameter estimation algorithm.

Press the **Store** button to overwrite your current parameters in the model with the estimated values.

**Note**:

You can estimate as many parameters as you want with this setup. Just note that the more uncertainty you introduce into your model the longer the algorithm can take to find a solution. It can also affect the probability of finding a solution.
